# Closed-Loop Chemical Recycling of a Biobased Poly(oxanorbornene-fused γ-butyrolactone)

**DOI:** 10.1021/jacs.4c12678

**Published:** 2024-12-03

**Authors:** Eva Harsevoort, Răzvan C. Cioc, Martin Lutz, Arnaud Thevenon, Pieter C. A. Bruijnincx

**Affiliations:** † Organic Chemistry and Catalysis, Institute for Sustainable and Circular Chemistry, Faculty of Science, Utrecht University, Universiteitsweg 99, 3584 CG Utrecht, The Netherlands; ‡ Structural Biochemistry, Bijvoet Centre for Biomolecular Research, Faculty of Science, Utrecht University, Universiteitsweg 99, 3584 CG Utrecht, The Netherlands

## Abstract

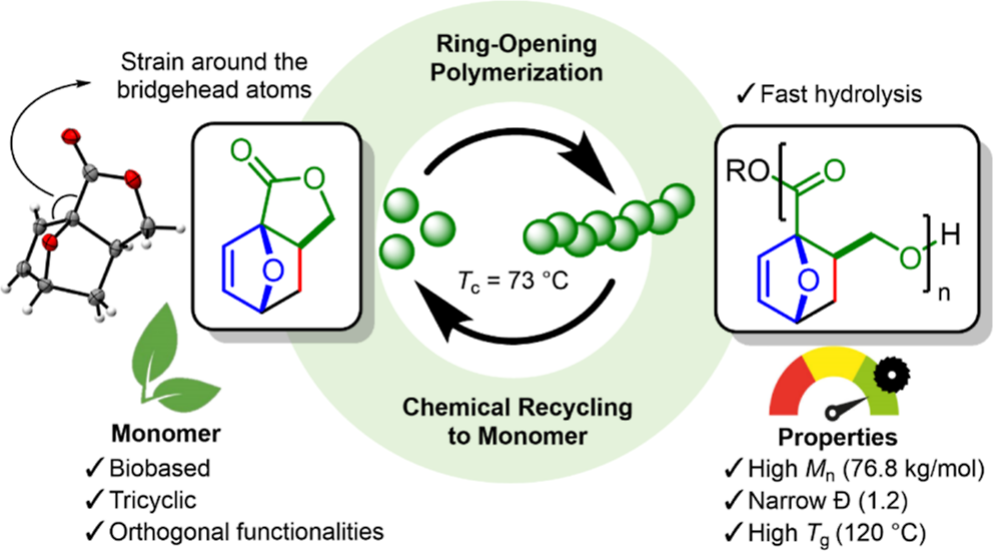

New polymers, properly designed for end-of-life and efficiently formed from renewable carbon, are key to the transition to a more sustainable circular plastics economy. Ring-opening polymerization (ROP) of bicyclic lactones is a promising method for the production of intrinsically recyclable polyesters, but most lactone monomers lack an efficient synthesis route from biobased starting materials, even though this is essential to sustainably account for material loss during the life cycle. Herein, we present the exceptionally rapid and controlled polymerization of a fully biobased tricyclic oxanorbornene-fused γ-butyrolactone monomer (**M1**). Polyester P(**M1**) was formed in low dispersity (D̵ = 1.2–1.3) and controllable molecular weight up to *M*_n_ = 76.8 kg mol^–1^ and exhibits a high glass transition temperature (*T*_g_ = 120 °C). The orthogonal olefin and lactone functionalities offer access to a wide range of promising materials, as showcased by postpolymerization modification by hydrogenation of the olefin, which increased polymer thermal stability by over 100 °C. Next to rapid hydrolytic degradation and solvolysis, the poly(oxanorbornene-fused γ-butyrolactone) could be cleanly chemically recycled back to the monomer (CRM), in line with its favorable ceiling temperature (*T*_c_) of 73 °C. The density functional theory (DFT)-computed Δ*H*° of ring-opening with methanol of γ-butyrolactone-based monomers provided a model to predict *T*_c_, and the DFT-computed and X-ray crystal structure-derived structural parameters of **M1**, hydrogenated analogue **M1-H**_**2**_, and regioisomer **M2** offered insights into the structural descriptors that cause the high polymerizability of **M1**, which is key to establishing structure–property relations.

## Introduction

The current linear plastics economy must undergo a major transformation to deal with the escalating plastic pollution^1^ and its contribution to rising greenhouse gas emissions,^2,3^ as well as to sustainably meet the anticipated growth in plastics demand.^4^ Although recycling the waste of existing plastics is of great importance,^5^ it is equally important to develop the next generation of plastics. These new plastics should be designed with their end-of-life in mind, making them readily (chemically) recyclable and/or biodegradable.^6^ Moreover, they have to be synthesized from renewable carbon sources with building blocks readily accessible from biomass or CO_2_ in atom and redox-efficient ways. In a circular plastics economy, this ensures a sustainable intake of renewable virgin carbon to make up for the inevitable material losses.

Chemical recycling to monomer (CRM)—the clean depolymerization of a polymer to its monomer building blocks—is a promising approach to move toward a circular plastics economy. It allows materials to retain their value and quality upon recycling in an energy- and resource-efficient manner.^7^ As the current slate of in particular polyolefins is not suited for such a strategy, alternatives need to be sought. Here, ring-opening polymerization (ROP) of lactones presents a particularly attractive approach to the design of polyesters suitable for CRM.^7−10^ Energy-efficient ROP and CRM require the design of structures with a suitable ceiling temperature (*T*_c_). A major challenge is then the trade-off between polymerization and depolymerization; i.e., lactones that polymerize very well (high *T*_c_) are often difficult to ring-close for CRM and vice versa. For example, γ-butyrolactone (γBL, low *T*_c_ of −136 °C) requires industrially challenging polymerization conditions of −40 °C,^11^ while poly ε-caprolactone (εCL, high *T*_c_ of >2000 °C) can only undergo CRM at high temperatures and under vacuum to shift the unfavorable thermodynamics toward the monomer side.^12^

**Scheme 1:**
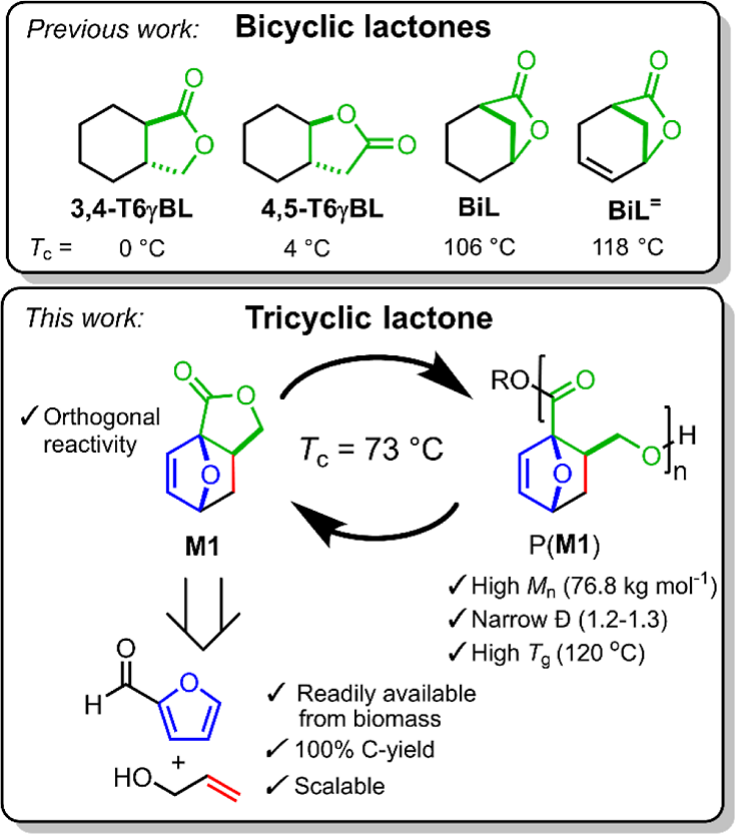
Multicyclic Lactone Monomers for ROP

A promising strategy to tailor *T*_c_ is to design fused or bridged bicyclic monomers, especially including a five-membered lactone.^10,13−17^ This introduces strain into the monomer, of which the γBL core is otherwise thermodynamically difficult or even impossible to polymerize.^11^ A notable example of such a bicyclic system is a bridged monomer that could be regarded as a structural combination of γBL and εCL (a low and high *T*_c_ monomer, respectively), forming the bicyclic lactone BiL (see Scheme 1), as reported by Chen et al.^14^ This bicyclic lactone shows a suitable *T*_c_ and, accordingly, can be polymerized and recycled to the monomer at moderate temperatures. Generally, these bicyclic systems have *T*_c_’s suitable for efficient polymerization in the range of 0–118 °C (see Scheme 1), affording polyesters with decent physical properties that can also efficiently undergo CRM.^10^

While developments on the promising methodology of fused lactone ROP/CRM are highly exciting, the number of examples of (poly)lactones with appropriate properties is still very limited.^18^ Further progress is hampered, for one, by the lack of a fundamental understanding about the structure–property relationships that govern (de)polymerization. Indeed, insight into how molecular structure influences the capacity of these five-membered lactone monomers to undergo ROP/CRM is required for the rational design of novel monomers and to more fully deliver on the promise of lactone ROP. Besides thermodynamic and kinetic polymerization considerations, ready access from renewable resources is equally imperative for the design of a new monomer. While (some of) the bicyclic monomers in Scheme 1 are accessible in high yield, their syntheses suffer from a poor atom economy, and most importantly, they lack an efficient route from biobased starting materials.

**Scheme 2:**
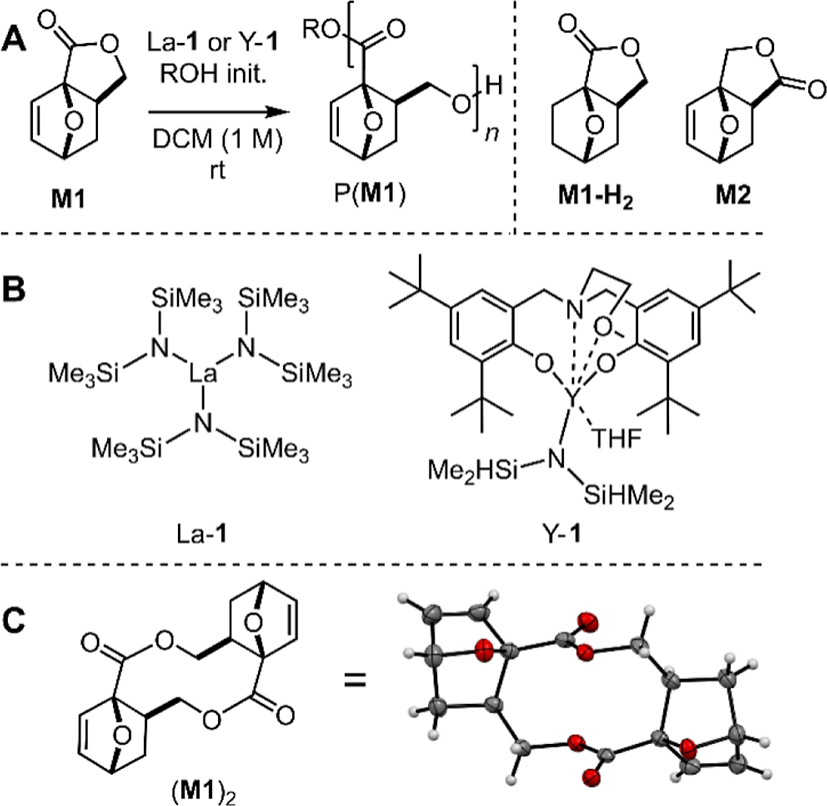
(A) Reaction Scheme of ROP of Monomer **M1** to Form Polyester P(**M1**) and the Structure of Regioisomers **M2**(21) and **M1-H**_**2**_,^19^ (B) Chemical Structures of the Catalysts Used, and (C) Chemical Structure and Single-Crystal Structure of the Cyclic Dimer (**M1**)_2_ (Ellipsoids Are Drawn at 50% Probability)

Recently, we have reported the scalable synthesis of the biobased, oxanorbornene-fused γBL monomer **M1** (see Scheme 1).^19^ This tricyclic monomer is formed from cheap, renewable building blocks in high atom economy (100% carbon yield) using catalytic steps. Despite the stereochemical structural complexity of **M1**, the monomer is formed as the racemic mixture of a single *ortho*-*exo* diastereomer due to the regio- and stereoselectivity of the intramolecular Diels–Alder (DA) reaction. The structure of **M1** allows for orthogonal reactivity: next to a lactone for ROP to form polyesters (described here), this monomer contains an olefin for ring-opening metathesis polymerization to form polyenes,^20^ much like the orthogonal reactivity reported for BiL^=^.^13^**M1** is the prototypical example of a broader family of furan-derived, substituted tricyclic DA adducts, one which also includes the hydrogenated analogue **M1-H**_**2**_ and the previously reported regioisomeric lactone monomer **M2** (see Scheme 2A),^21^ which differs from **M1** in the position of the carbonyl group.

Herein, we report the first example of an γBL-fused tricyclic monomer, demonstrating both exceptionally high polymerization rates and chemical depolymerization back to the monomer. Controlled polymerization of **M1** yields amorphous, high *T*_g_ biobased polyesters with high molecular weight while maintaining low dispersity. Furthermore, we show that postpolymerization modification of the oxanorbornene unit allows the polymer properties to be tuned. Notably, the system exhibits a *T*_c_ of 73 °C, allowing for efficient CRM, thereby achieving circularity. Besides CRM, various other recycling routes are demonstrated for P(**M1**), including hydrolytic degradation. Finally, from density functional theory (DFT)-computed thermodynamic parameters of the ring-opening of **M1** and similar monomers, we propose a model to predict the *T*_c_ values of γBL-based monomers. Furthermore, a comparison of single-crystal and DFT-computed structures of **M1** and related monomers revealed important structural descriptors for polymerizability.

## Results and Discussion

### Polymer Formation

ROP of **M1** (Scheme 2A) was screened with La(HMDS)_3_ (La-**1**), Y-**1**, and triazabicyclodecene (TBD) (Scheme 2B), of which the first two resulted in formation of P(**M1**) at [**M1**]_0_ = 1 M. First, commercially available La-**1**, previously reported to be an effective polymerization catalyst for various five-membered mono-^11,22^ and multicyclic lactones,^14−16^ was tested. Excitingly, **M1** was efficiently and cleanly polymerized at room temperature with La-**1** (1 mol %) in the presence of various alkoxide initiators (2 mol %) (Table 1). ROP of **M1** quickly reached equilibrium conversion of *ca*. 80% in only 15 min, independently of the initiator used [e.g., isopropanol (^*i*^PrOH), benzyl alcohol (BnOH), and diphenylmethanol (Ph_2_CHOH)] (Tables 1 and S1). A higher initial monomer concentration ([**M1**]_0_ = 2 M, entry 4) only slightly improved the monomer conversion while broadening the dispersity (D̵ = 1.46). Moderate conversion (47%) was achieved with the bulky initiator triphenylmethanol (Ph_3_COH) (Table S1, entry S5), which is known to have a lower or no activity with certain monomers, such as γBL.^11^

**Table 1:** Selected ROP Results of **M1**^a^

entry	Cat.	Init.	ratio [M]/[Cat.]/[I]	**M1** Conv.^b^ (%)	isolated yield (%)	*M*_n,calc_ (kg mol^–1^)^c^	*M*_n,GPC_ (kg mol^–1^)^d^	D̵^e^
1	La-**1**	^*i*^PrOH	100/1/2	80	51	6.1	24.8	1.42
2^f^	La-**1**	BnOH	100/1/2	80	61	6.0	22.3	1.36
3	La-**1**	BnOH	100/1/3	83	55	4.2	19.3	1.31
4^g^	La-**1**	BnOH	100/1/2	84	60	6.5	24.9	1.46
5	La-**1**	BnOH	200/1/2	78	63	11.9	30.4	1.32
6	La-**1**	BnOH	400/1/2	80	63	24.4	47.3	1.34
7	La-**1**	BnOH	600/1/2	79	68	36.2	55.6	1.25
8	La-**1**	BnOH	1000/1/2	76	70	57.9	76.8	1.19
9	Y-**1**	^*i*^PrOH	100/1/1	83	58	12.7	29.8	1.25
10^h^	Y-**1**	^*i*^PrOH	400/1/1	82	69	50.0	47.5	1.36
11	Y-**1**	BnOH	100/1/1	81	64	12.4	29.4	1.27
12^i^	TBD	BnOH	100/1/2	0				

a Conditions: **M1** (152 mg, 1.0 mmol), initiator (I): isopropanol (^*i*^PrOH), benzyl alcohol (BnOH), [**M1**]_0_ = 1 M in DCM, rt, 15 min, stirring speed: 50 rpm. Selectivity for P(**M1**) was >99% for all entries.

b Monomer conversion determined by ^1^H NMR analysis.

c *M*_n,calc_ = conversion × [**M1**]/[Init.] × MW of monomer + MW of initiator.

d *M*_n,GPC_ determined by gel permeation chromatography at 65 °C in *N*,*N*-dimethylformamide, calibrated with polystyrene standards.

e D̵ = *M*_w_/*M*_n_.

f Average result of five experiments; see Table S2 for standard deviations.

g Performed with [**M1**]_0_ = 2 M in DCM.

h Conversion after 15 min was 8%; the polymerization was quenched after 7 h when the equilibrium was reached.

i [**M1**]_0_ = 0.8 M in DCM, 2 h.

Analysis of the resulting polymers by GPC showed that ROP in the presence of ^*i*^PrOH or BnOH is controlled, forming polymers with a molecular weight of ca. *M*_n,GPC_ = 23 kg mol^–1^ and moderately narrow dispersity (D̵ = 1.3–1.4) (Table 1, entries 1 and 2). Promisingly, high molecular weight polymers (76.8 kg mol^–1^) were also afforded at a catalyst loading as low as 0.1 mol % with narrower dispersity (D̵ = 1.19) (entry 8).

Increased equivalents of alcohol initiator typically result in lower dispersity and slightly lower molecular weight,^11,16^ and as expected, 3 equiv of BnOH (Table 1, entry 3) decreased the molecular weight as well as the dispersity, compared to 2 equiv of BnOH (entry 2). Similar results were obtained with ^*i*^PrOH when comparing 2 equiv (entry 1) to 1 equiv of initiator (Table S1, entry S1). Note that *M*_n,calc_ is based on all equivalents of ROH initiating polymerization.

Second, the ROP of **M1** was tested in the presence of yttrium catalyst Y-**1**, made from a tetradentate amino-bisphenolate ligand. Previously reported by Carpentier et al.,^23^ this type of catalyst has been extensively studied for lactone ROP^24^ (e.g., with lactide,^25^ βBL,^26^ γBL,^11^ 3,4-T6γBL,^15^ and BiL^=13^).

The polymerization of **M1** with Y-**1** in the presence of ^*i*^PrOH or BnOH as the initiator resulted in the formation of P(**M1**), with a narrower dispersity (D̵ = 1.25) compared to La-**1**. Similarly to La-**1**, equilibrium conversion was reached within 15 min at 25 °C. However, the polymerization is slower at lower catalyst loading, where, for instance, 8% monomer conversion was observed after 15 min at 0.25 mol % of Y-**1**, and equilibrium was reached only after 7 h (entry 10). Polymerization with triazabicyclodecene (TBD), a common bifunctional organocatalyst for lactone ROP (e.g., with γ-lactones,^11^ substituted δ-lactones,^27,28^ and substituted ε-lactones^29^) did not result in the formation of P(**M1**) (entry 12). ROP experiments of both enantiopure (*R*,*R*,*R*)-**M1** and (*S*,*S*,*S*)-**M1** are part of ongoing studies aimed at providing more insight into, among others, the tacticity in P(**M1**).

### Selectivity of the Polymerization

Unexpectedly, a side product slowly appeared during the polymerization of **M1** at higher stirring rates and prolonged reaction times. For instance, it formed with a spectroscopic yield as high as 14% when the polymerization reaction mixture was stirred at 1000 rpm for 2 h (Table S3, entry S6) in the presence of La-**1**. Crystals of the side product, suitable for single-crystal analysis, revealed that it is a ten-membered-ring compound [(**M1**)_2_, Scheme 2C] composed of two ring-opened monomers of the same enantiomer of **M1**. To shed more light on how (**M1**)_2_ is formed, a polymerization of **M1**, performed at 50 rpm in the presence of La-**1** to give P(**M1**) with *M*_n_ = 23.8 kg mol^–1^, was then left to stir at 1000 rpm for up to five additional hours (Table S4). Interestingly, the *M*_*n*_ decreased to 19.0 kg mol^–1^ after 2 h and to 15.9 kg mol^–1^ after 5 h, while the amount of (**M1**)_2_ continuously increased. The same experiment with Y-**1** also formed (**M1**)_2_, albeit in lower amounts (5% after 24 h, Table S5, entry 3), indicating that its formation is metal-catalyzed. This data suggest that the cyclic dimer is formed from the polymer by backbiting and is therefore the thermodynamic product of the ring-opening reaction, the formation of P(**M1**) being the kinetic product. This was further confirmed by the Gibbs free energy calculated with DFT for the formation of (**M1**)_2_ by dimerization of **M1**, which is more exergonic (−2.7 kcal mol^–1^**M1**) than the simple ring-opening of **M1** with, e.g., methanol (−1.7 kcal mol^–1^**M1**).

As expected from the lack of ring strain (see below), the cyclic dimer (**M1**)_2_ did not polymerize under the conditions used for the ROP of **M1**. Macrolactones may undergo entropically driven polymerization, regulated by a floor temperature (*T*_f_) instead of a ceiling temperature (*T*_c_),^30^ and recently, ROP of an 18-membered macro-dilactone monomer derived from biomass was reported.^31^ We are currently investigating the potential of the dimer (**M1**)_2_ and hydrogenated dimer (**M1-H**_**2**_)_2_ for entropy-driven ROP in our laboratory. Note that under optimized conditions as listed in Table 1, formation of (**M1**)_2_ can be fully avoided, and polymerization occurs with excellent selectivity (>99%, only polymer visible by ^1^H NMR analysis).

### Fast and Living Polymerization

**Figure 1:**
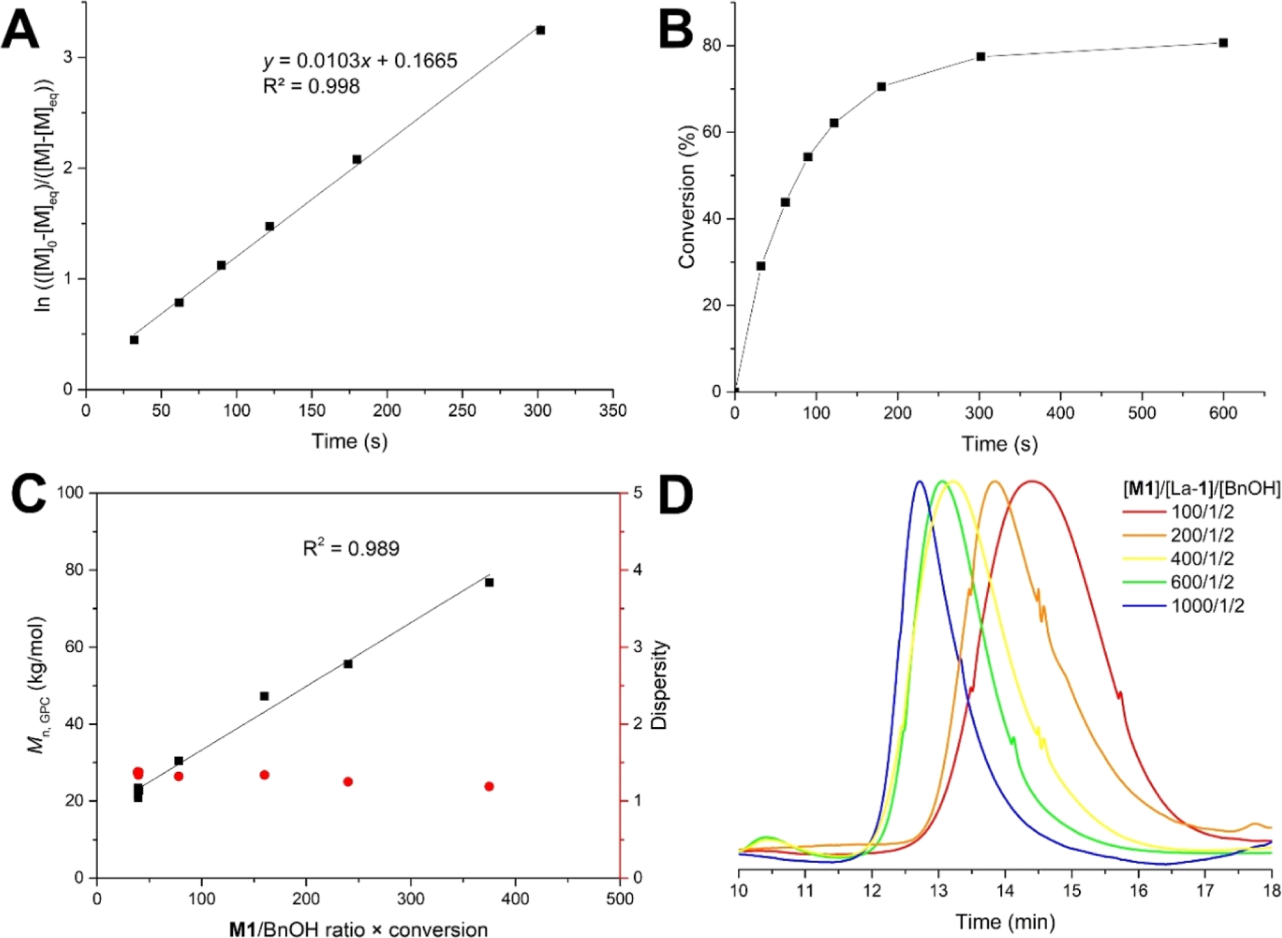
Fast and controlled polymerization. (A) Semilogarithmic kinetic plot indicating first-order in **M1** and (B) monomer conversion over time for **M1**/La-**1**/BnOH = 100/1/2 (0 °C, 0.5 M). (C) Plot of *M*_n,GPC_ (black) and *D̵* (red) of P(**M1**) as a function of the monomer loading (**M1**/BnOH ratio × conversion) with the BnOH/La-**1** ratio fixed at 2/1 (Table 1, entries 2 and 5–8) and (D) overlay of the GPC traces of the polymerizations with different **M1**/La-**1**/BnOH ratios.

Kinetic studies show that the polymerization of **M1** with La-**1** follows first-order kinetics in monomer concentration, as typically observed with other lactones.^32^ The observed rate, extracted from the linear correlation of the semilogarithmic plot vs polymerization time (Figure 1A), gave *k*_obs_ = 1.03 × 10^–2^ s^–1^ for [**M1**]_0_ = 0.5 M. Already after 10 min, an equilibrium conversion of 81% is achieved (Figure 1B), and stirring for an additional 50 min does not increase the dispersity, indicating that no significant transesterification or backbiting takes place under these conditions (Table S6). Furthermore, *M*_n_ linearly increased with conversion (Figure S2A) as well as with the decreasing catalyst loading (Figure 1C), while the dispersity remained low (Figure 1D). All these observations indicate that the ROP of **M1** with La-**1**/ROH is rapid, yet controlled, and has a living character.

### Thermal Properties

**Figure 2:**
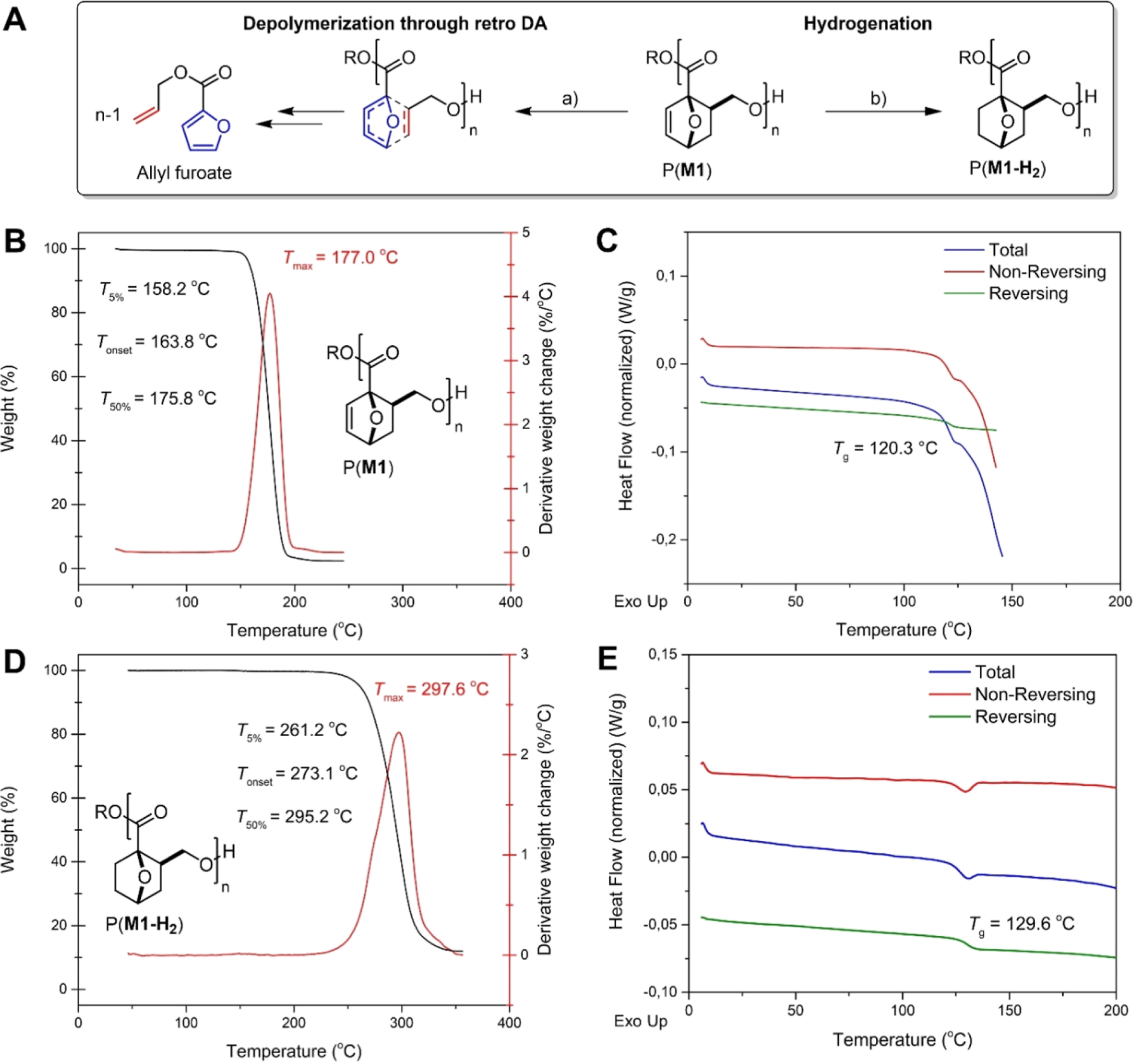
Thermal properties of P(**M1**) and P(**M1-H**_2_) and decomposition products. (A) Depolymerization pathway of P(**M1**) through the retro-Diels–Alder (rDA) reaction and blocking of rDA by postpolymerization hydrogenation of P(**M1**); (a) DMSO-*d*_6_, 150 °C, 1 h; (b) Pd/C, H_2_, DCM, rt, 6 h. (B) TGA (black) and DTG (red) curves for P(**M1**) obtained with **M1**/La-**1**/BnOH = 100/1/2 (Table 1, entry 2) and (C) MDSC thermogram of P(**M1**) (second heating scan). (D) TGA (black) and DTG (red) curves for P(**M1-H**_**2**_) obtained by postpolymerization hydrogenation of P(**M1**) obtained with **M1**/Y-**1**/^*i*^PrOH = 100/1/1 (Table 1, entry 9) and (E) MDSC thermogram of P(**M1-H**_**2**_) (second heating scan).

Next, the thermal properties of P(**M1**) were analyzed by modulated differential scanning calorimetry (MDSC) and thermal gravimetric analysis (TGA). The MDSC trace shows only a glass transition temperature (*T*_g_) at 120.3 °C (Figure 2C) and no melting event, suggesting that P(**M1**) is completely amorphous. Notably, the *T*_g_ is significantly higher than that of other *trans*-fused bicyclic five-membered lactones, such as 3,4-T6γBL^15^ and 4,5-T6γBL^16^ (*T*_g_ = 49 and 75 °C, respectively), and closer to bridged five-membered lactones like BiL^14^ and BiL^=13^ (*T*_g_ = 135 and 110 °C, respectively). The rigidity imposed by the oxa-bridge is suggested to contribute to the observed high *T*_g_.

The intrinsic thermolability of the oxanorbornene moiety in P(**M1**) can be leveraged to obtain full and clean thermal depolymerization into allyl furoate through the retro-Diels–Alder (rDA) reaction (Figure 2A), as also described below (Figure 6E). The TGA curve of P(**M1**) and its derivative (DTG) showed that weight loss takes place in one, well-defined step, assumedly caused by allyl furoate evaporation (Figure 2B). Accordingly, the *T*_5%_ (158 °C) is lower than those of polymers from multicyclic lactone ROP that do not contain such a thermolabile design feature (*T*_5%_ = 287, 318, 340, and 304 °C for BiL^=^, BiL, 3,4-T6γBL, and 4,5-T6γBL, respectively). As TGA detects weight loss and not depolymerization, solid- and solution-state thermal stabilities were further assessed. ^1^H NMR analysis of P(**M1**) after heating in the solid state for MDSC to 130 °C (well below the *T*_onset_ measured by TGA) showed 5% allyl furoate formation. In DMSO-*d*_6_ solution, heating P(**M1**) (with *M*_n,GPC_ = 21.7 kg mol^–1^ and D̵ = 1.31) to 100 °C for 1 h resulted in 2% allyl furoate formation; chain scission caused by the rDA pathway decreased the molecular weight and increased the dispersity of the polymer (*M*_n,GPC_ 12.5 kg mol^–1^; D̵ = 1.37).

### Formation of P(**M1-H**_**2**_)

The built-in and exploitable thermolability of P(**M1**) can be readily removed by postpolymerization modification, blocking the rDA depolymerization pathway. To form polymers with improved thermostability, the olefinic bond in P(**M1**) was hydrogenated over Pd/C under 1 bar of H_2_ at rt. Analysis of the resulting polymer P(**M1-H**_**2**_) indicated that >99% of the olefins were successfully hydrogenated while not affecting *M*_n_ or dispersity (see Table S10). Thermal analysis of P(**M1-H**_**2**_) showed that *T*_g_ is relatively unaffected by the postpolymerization hydrogenation with only a small increase of ca. 10 °C (Figure 2E). In sharp contrast, the thermal stability increased by more than 100 °C, reaching a value very similar to that of closely related polyesters (*T*_5%_ = 261 °C) for P(**M1-H**_**2**_).

Alternatively, we investigated whether P(**M1-H**_**2**_) could directly be formed from the ROP of the hydrogenated monomer **M1-H**_**2**_. Interestingly, however, **M1-H**_**2**_, though structurally highly similar (see below), was found to be much less reactive in ROP than **M1**, and no polymerization was observed at 0 °C with [**M1-H**_**2**_]_0_ = 1.0 M. At subzero temperatures, P(**M1-H**_**2**_) could be obtained, and, although similar monomer conversion as for **M1** can be obtained at −40 °C (75%), reaction times over 24 h were required. Furthermore, the polymerization of **M1-H**_**2**_ is not well controlled; unexpected high molecular weight (*M*_n,GPC_ = 48.7 kg/mol and *M*_n,NMR_ = 47.3 kg/mol compared to *M*_n,calc_ = 5.8 kg/mol) and broad dispersity (D̵ = 2.71) (see Table S8) were obtained from isolated P(**M1-H**_**2**_) formed at −40 °C. The requirement of a lower temperature indicates that the ring strain imposed on the system by the olefinic bond is essential for the high reactivity, which was further confirmed by determination of the *T*_c_ and DFT calculations (see below).

### Relationship between *T*_c_ and Ring Strain

**Figure 3:**
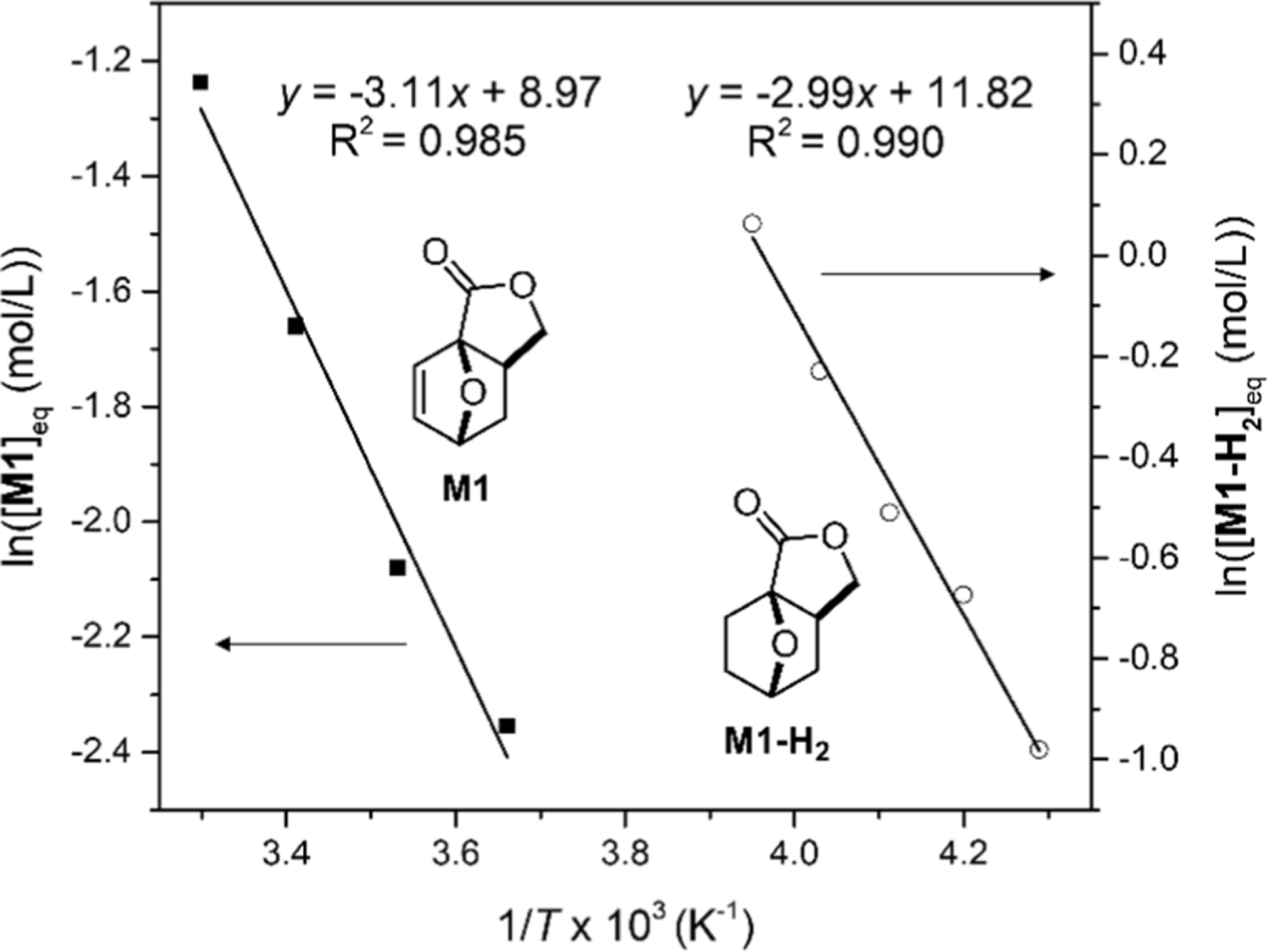
Van ‘t Hoff plot for polymerization of **M1** and **M1-H**_**2**_.

The ceiling temperature (*T*_c_) of the bridged, tricyclic **M1** and **M1-H**_**2**_ was extracted from a Van ‘t Hoff plot (Figure 3).^32^ The standard-state thermodynamic parameters obtained for **M1** are Δ*H*_p_° = −25.8 kJ mol^–1^ and Δ*S*_p_° = −74.6 J mol^–1^ K^–1^, giving a *T*_c_ = 73 °C at [**M1**]_0_ = 1 M. This is higher than the *T*_c_ of other fused five-membered lactone monomers reported in the literature, such as 3,4-T6γBL (*T*_c_ = 0 °C) and 4,5-T6γBL (*T*_c_ = 4 °C), and closer to bridged bicyclic monomers BiL and BiL^=^ (*T*_c_ = 106 and 118 °C, respectively). For **M1-H**_**2**_, the parameters are Δ*H*_p_° = −24.8 kJ mol^–1^ and Δ*S*_p_° = −98.3 J mol^–1^ K^–1^, resulting in a *T*_c_ of −21 °C at [**M1-H**_**2**_]_0_ = 1 M. The hydrogenation of the endocyclic C = C bond drastically lowers the *T*_c_ (Δ*T*_c_ = 94 °C), a much larger difference than that observed for BiL^=^ and BiL (Δ*T*_c_ = 12 °C).

As it reflects the equilibrium of polymerization and depolymerization, a finely tuned *T*_c_ is an essential parameter to control for both energy-efficient polymer formation (avoiding industrially unattractive low temperatures due to a low *T*_c_) and CRM (avoiding high energy costs for depolymerization due to a high *T*_c_). A simple model that enables the prediction of the *T*_c_ would be highly beneficial for circular polymer development.^33,34^ While it remains challenging to accurately compute thermodynamic parameters of ROP, calculations on simple model reactions can already give qualitative insights into trends in *T*_c_.^33,34^ With our novel results on **M1** and **M1-H**_**2**_, combined with other examples from the literature, we investigated whether the Δ*H*° of a simple model reaction could be a good descriptor to predict *T*_c_ since these polymerizations are typically enthalpically driven.

**Figure 4:**
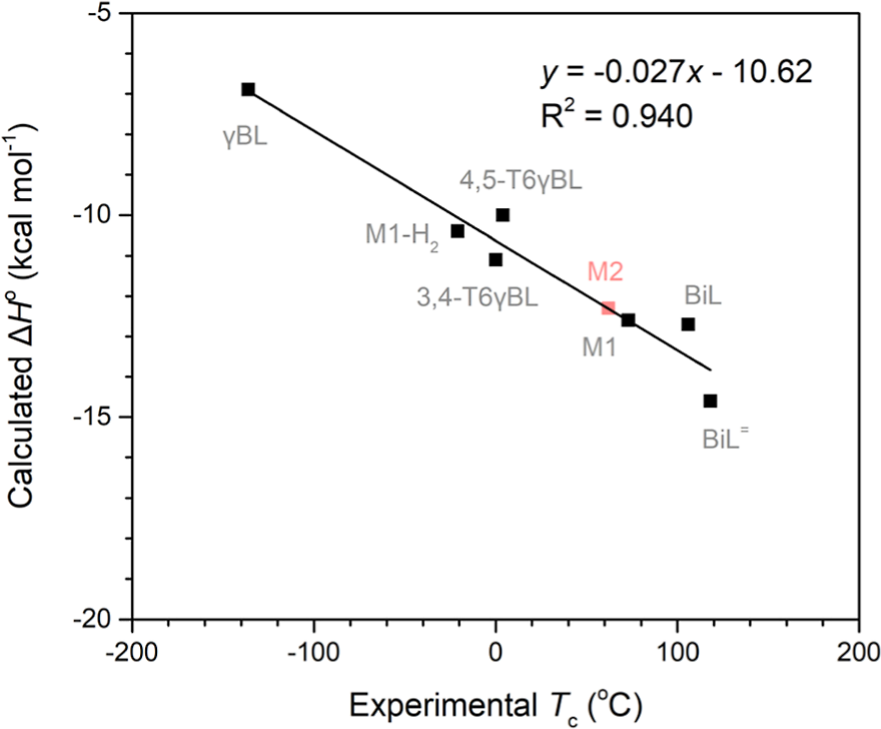
Linear correlation of DFT-calculated Δ*H*° and experimental (black) and predicted (red) *T*_c_ of the γBL-based monomers.

Δ*H*° and Δ*G*° were computed for the ring-opening of the γBL-based monomers with methanol with density functional theory (DFT) (Table 2). The results show that Δ*H*° linearly correlates with the reported experimentally determined *T*_c_ (Figure 4). Although the margins are narrow and close to the chemical accuracy (ca. 1 kcal mol^–1^) of the calculations, the scale derived from this simple model nevertheless accurately reflects the differences in *T*_c_ between the unstrained γBL,^11^ moderately strained monomers 3,4-T6γBL^15^ and 4,5-T6γBL,^17^ the herein reported monomer **M1**, and even more strained bridged monomers BiL^14^ and BiL^=^.^13^*Cis*-cyclohexane-fused γBL (3,4-C6γBL) and phthalide are known not to undergo polymerization, and indeed, calculations of the ring-opening of these monomers with methanol resulted in high positive Δ*G*° values. Rewardingly, the calculations also support the experimental observation that hydrogenation of the endocyclic C=C bond lowers the polymerization tendency, as exemplified for both **M1-H**_**2**_ compared to **M1** (ΔΔ*H*° = 2.2 kcal mol^–1^) and BiL and BiL^=^ (ΔΔ*H*° = 1.9 kcal mol^–1^).

**Table 2:**
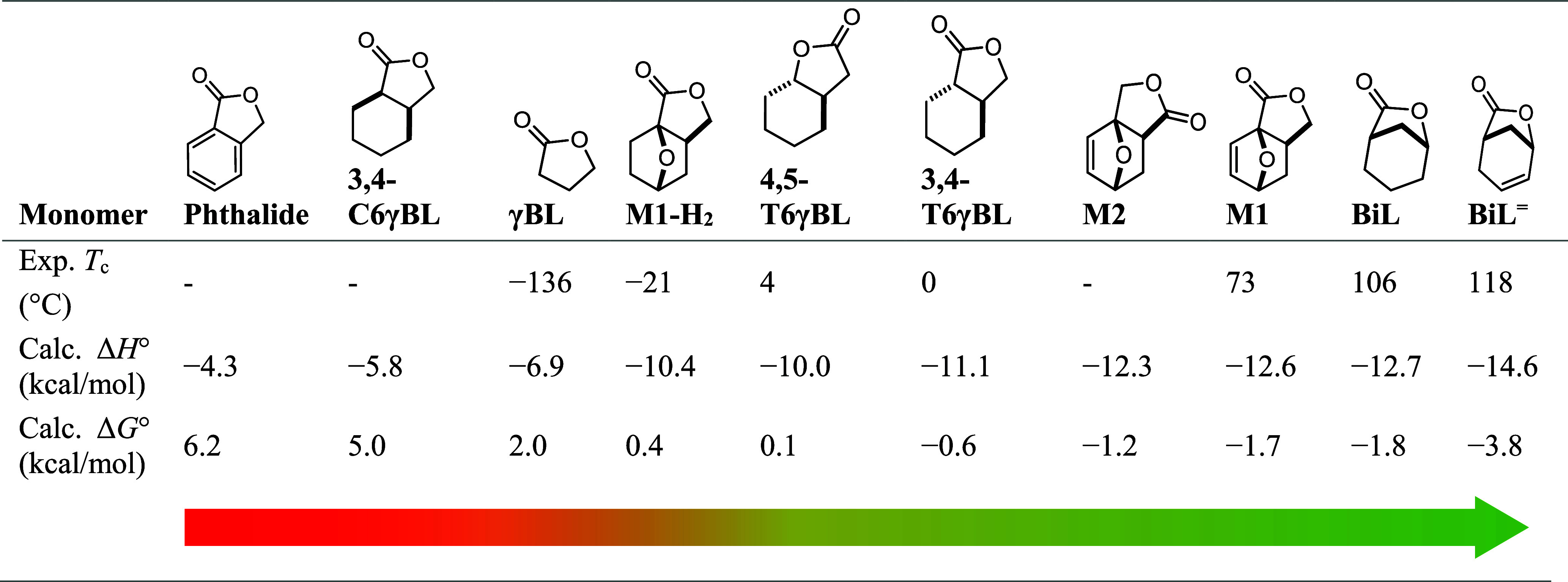
Computational Results^a^

a Experimentally obtained ceiling temperature (*T*_c_) as reported in literature (γBL,^11^ 4,5-T6γBL,^17^ 3,4-T6γBL,^15^ BiL,^14^ and BiL^=13^), and the enthalpies (Δ*H*°) and free energies (Δ*G*°) of the ring-opening with MeOH as a model reaction, calculated with DFT (b3lyp/6-311 + g(d,p) empirical dispersion = gd3bj). The arrow indicates the propensity for ring opening as calculated by DFT (Δ*G*°).

Interestingly, the calculated thermodynamic parameters of lactone **M2** are highly similar to those of **M1**, indicating its potential for ring-opening. From Figure 4, a *T*_c_ of roughly 62 °C would be expected for **M2** (indicated in red). However, surprisingly, our attempts to polymerize **M2** were unsuccessful with either La-**1** or Y-**1**. This indicates that other factors besides the thermodynamic driving force of ring strain release play a role in the polymerizability of γBL-based monomers, as discussed further below.

### Understanding the Differences in Reactivity between **M1**, **M1-H**_**2**_, and **M2**

**Figure 5:**
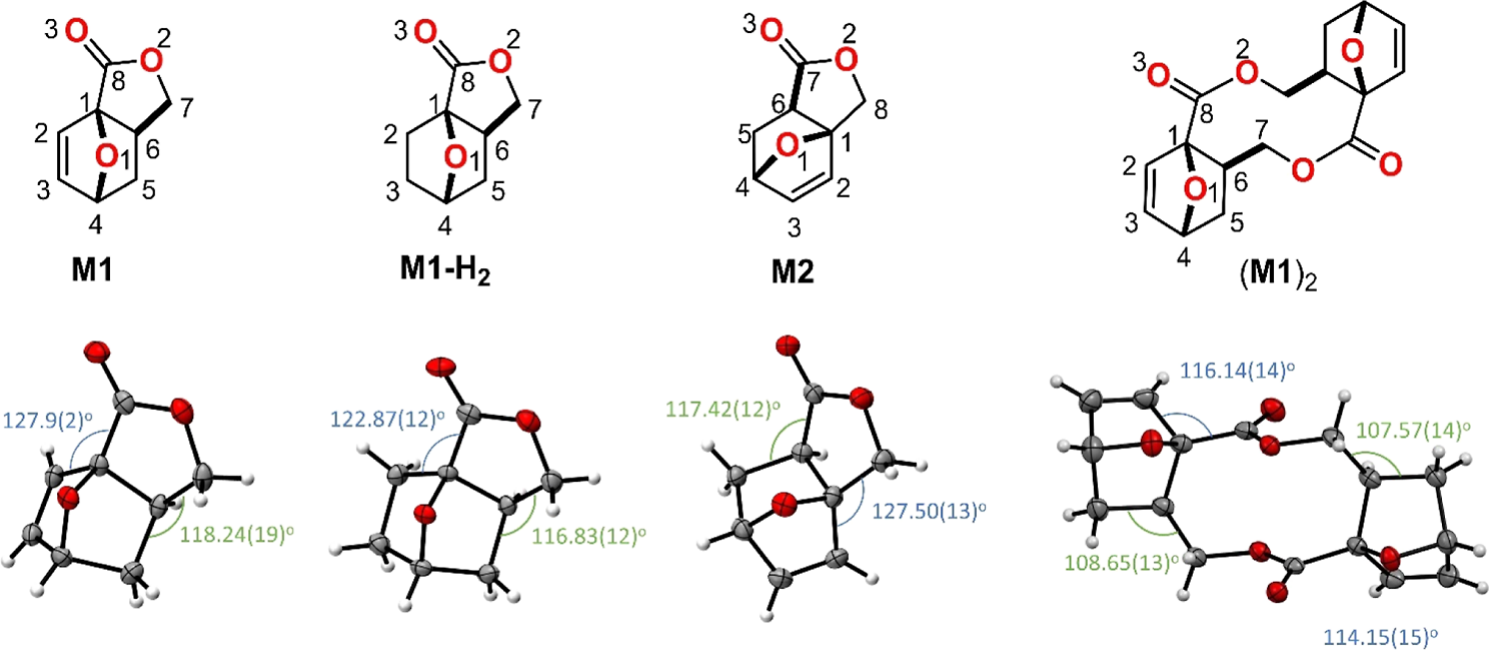
Solid-state structures of the monomers. Ellipsoids are drawn at 50% probability, and relevant angles are highlighted in green and blue, indicating the angular strain in **M1**, **M1-H**_**2**_, **M2**,^21^ and (**M1**)_2_.

Despite their structural similarity, the monomers show very different polymerization behavior: **M1** polymerized very well, **M1-H**_**2**_ moderately so, and **M2** not at all. This is therefore an interesting case study to gain insight into the underlying structure–property relations to better understand the factors that govern the differences in ring-opening propensity of **M1**, **M1-H**_**2**_, and **M2**, besides ring strain. The geometric parameters obtained from the X-ray crystal structures of **M1**, **M1-H**_**2**_, and **M2**(21) (Figure 5) show that the lactone rings of **M1**, **M1-H**_**2**_, and **M2** are in fact geometrically quite similar. For all three monomers, the five-membered lactone core adopts an envelope conformation, with the β-carbon (C6 for **M1**/**M1-H**_**2**_ and C1 for **M2**) in the *endo* position (out of plane) (Figure S6), and the bond lengths, bond angles, and torsions are similar with maximum differences of 0.03 Å, 2.0°, and 3.6°, respectively (Table S14), except for the internal angle around C1/C6. The largest deviations from the ideal angle were found around bridgehead atoms C1 and C6 (Table S16). The single-crystal-derived angles of **M1** are unusual for an sp^3^ configuration, with an angle of 127.9(2)° for < C2–C1–C8 (in blue) and 118.24(19)° for < C5–C6–C7 (in green, Figure 5). Similar values are observed in the structure of **M2**, while **M1-H**_**2**_ has somewhat more relaxed angles around the bridgehead carbons, in line with the lower calculated Δ*H*° in Table 2 and the observed lower conversion of **M1-H**_**2**_. Expectedly, the same angles for (**M1**)_2_ are even more relaxed. These angles may thus be regarded as good indicators to qualitatively estimate the ring strain in these systems.

DFT-calculated structures of the **M1**, **M1-H**_**2**_, and **M2** were compared with the “unstrained” parent γBL and oxanorbornene fragments. There is good agreement between the parameters of the crystal structures and the structures calculated with DFT (Tables S14–S16). The structural deviation of the lactone cores **M1**, **M1-H**_**2**_, and **M2** from the “unstrained” parent γBL is small, especially for **M1** and **M1-H**_**2**_. The internal bond angles of the lactone cycles deviate by a maximum of 2° from γBL (Table S14). Also, the oxanorbornene fragment in **M1** and **M2** is structurally very similar to the unsubstituted oxanorbornene (Table S15).

These results are in line with the structural parameters reported for 4,5-T6γBL and its *cis*-fused stereoisomer (4,5-C6γBL), for which it was suggested that torsion differences in the cyclohexane ring control the polymerizability rather than the torsion of the lactone ring.^17^ The strain from the twist boat conformation of the cyclohexane in the *trans*-fused lactones is reflected in their higher polymerizability, whereas the *cis*-fused isomer exists in a pseudochair and does not polymerize.^17^ For **M1** and **M1-H**_**2**_, however, the propensity for ring-opening stems from the fusion of the γBL ring to the oxanorbornene since both the lactone and oxanorbornene fragments themselves are not (additionally) strained.

From the DFT-calculated monomers and the MeOH ring-opened structures, it is observed that the angles around the bridgeheads of **M1** indeed relax toward more natural angles upon ring-opening of the lactone (Table S13). As expected, the same is true to a somewhat lesser extent for **M1-H**_**2**_. **M2** seems to be as strained as **M1** from the extent of angle relaxation, which is in line with the calculated thermodynamic parameters. The lack of ROP reactivity of **M2** is then not of thermodynamic origin but most likely of kinetic origin. In contrast to **M2**, the relative positioning of the carbonyl with respect to the electron-withdrawing oxa-bridge promotes nucleophilic attack on the carbonyl of **M1**, which is also experimentally observed from the rapid methanolysis, as shown below. All in all, the comparison of these monomers showcases that successful ROP indeed depends very subtly on the monomer structure.

### Chemical Recyclability of P(**M1**) and P(**M1-H**_**2**_)

**Figure 6:**
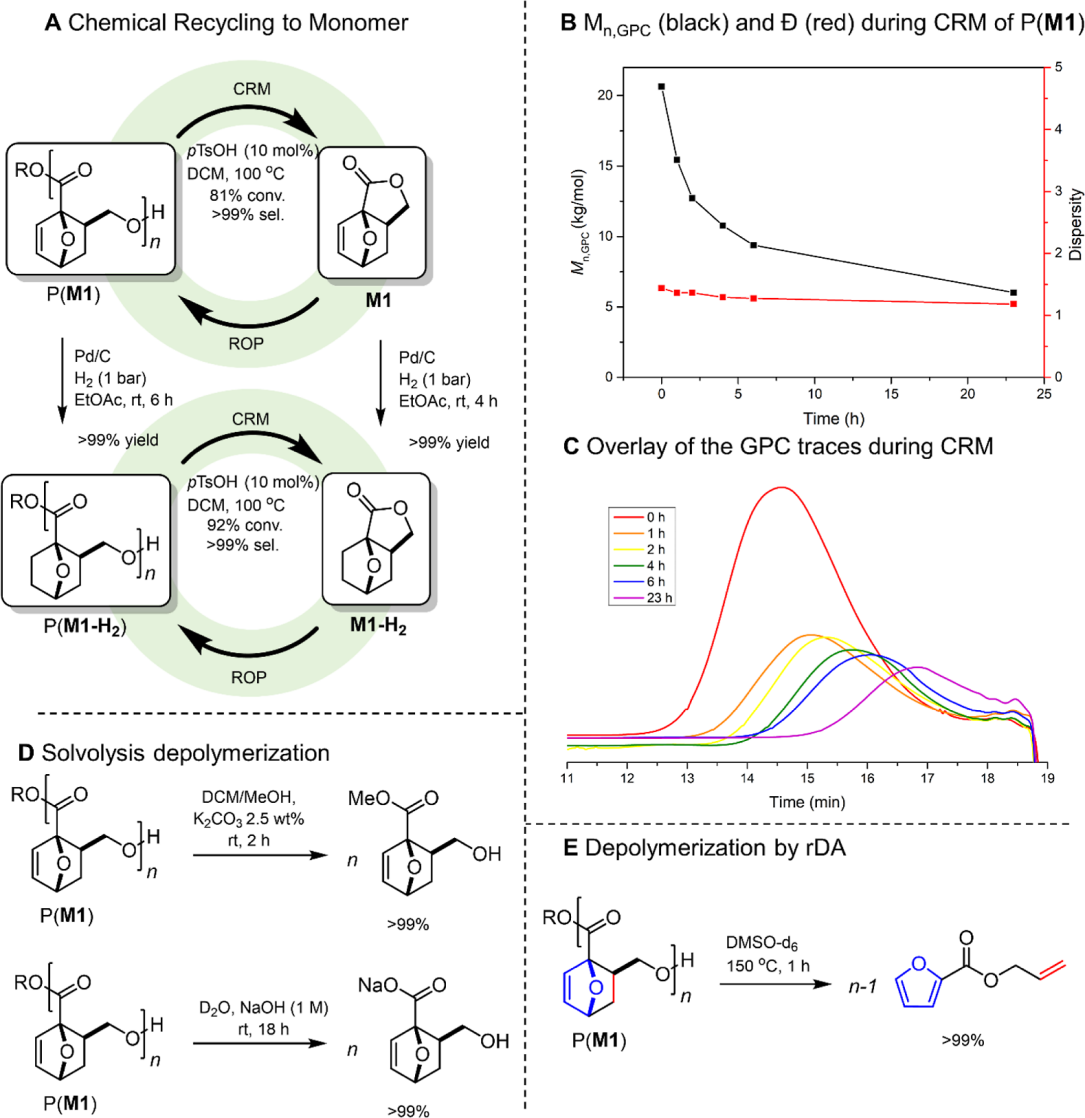
Depolymerization and chemical recycling to monomers of P(**M1**) and P(**M1-H**_**2**_).

Chemical recycling to monomer (CRM) of P(**M1**) was achieved selectively (>99%) with *p*TsOH as a catalyst at 100 °C. The P(**M1**) (formed with Y-**1**) conversion of 78% after 1 h increased only slightly to 81% after 6 h. Longer reaction times or an increase of catalyst loading led to a decrease in selectivity due to competition with the rDA reaction at elevated temperatures (Figure S3). The gradual decrease in *M*_n_ over time with a constant dispersity indicates that the depolymerization follows an unzipping pathway, and little to no random chain scission takes place (Figure 6B,C). The *M*_n_ continuously decreased with the extent of depolymerization (Figure S4), which suggests a controlled depolymerization having rapid, reversible chain transfer and a relatively short zip length (the average number of monomer units removed between an initiation and termination reaction during depolymerization).^35,36^

Using the same conditions, chemical recycling of P(**M1-H**_**2**_) resulted in 92% conversion to the hydrogenated monomer **M1-H**_**2**_ in excellent selectivity (>99%). Besides repolymerization to P(**M1-H**_**2**_), the monomer **M1-H**_**2**_ also has other outlets for valorization, e.g., by conversion to oxanorbornane phthalate analogues with the potential application as plasticizers.^19^

Next to CRM, polymer P(**M1**) could also be straightforwardly depolymerized to other monomeric building blocks. The polymer undergoes hydrolytic degradation rapidly into the repeat unit under basic aqueous conditions (rt, 18 h; Figure 6D). This is much faster than, e.g., P(γBL)^37^ (uncapped, ca. 4 d) and P(α-methylene-γBL) (120 d)^20^ under the same conditions and P(εCL) (88 d) at 2.5 M at 27 °C.^38^ This is promising for the ability of P(**M1**) and related structures to biodegrade, which will be the topic of further studies. The polymer could also be methanolized remarkably fast under mild catalytic conditions (Figure 6D). These results again confirm the versatile nature of the monomer/polymer family and, in particular, the susceptibility of **M1**/P(**M1**) for nucleophilic attack on the carbonyl. Furthermore, at 150 °C, the polymer was fully depolymerized to form the monomeric species allyl furoate through the rDA pathway. While allyl furoate cannot be used directly to regenerate the scaffold via DA cycloaddition, its constituents can be recovered and reused for that purpose.

## Conclusions

To conclude, the biobased, tricyclic γBL monomer **M1** proved to be a highly versatile scaffold that could be efficiently and cleanly converted to a renewable and intrinsically recyclable polymer by ROP. **M1** polymerization is rapid and controlled, yielding polyester P(**M1**) with >99% selectivity and with low dispersity (D̵ ∼ 1.2–1.3) and controllable molecular weight, up to 76.8 kg mol^–1^. The *T*_g_’s of P(**M1**) and P(**M1-H**_**2**_) are, at 120 and 129 °C, ca. 50–60 °C higher than other reported fused γ-lactones, broadening the scope of potential applications for this class of polymers. The oxanorbornene-fused scaffold further offers rich options for further chemical functionalization. Here, we exploited the orthogonality of the olefin and lactone functionalities through postpolymerization modification by olefin hydrogenation, forming P(**M1-H**_**2**_), which increased the moderate thermal stability of P(**M1**) by over 100 °C. **M1** thus presents the parent member of a larger family of monomers, with many options for functionalization or derivatization (post- or prepolymerization), some of which are currently under investigation. P(**M1**) was found to have excellent end-of-life properties, with a *T*_c_ of 73 °C and various options for depolymerization, including clean CRM. Alternative ways of recycling P(**M1**) through hydrolytic degradation and solvolysis (both >99% spectroscopic yield) are promising for the biodegradability potential of these polymers.

The correlation of the *T*_c_ of **M1**, **M1-H**_**2**_, and similar reported monomers against DFT-computed thermodynamic parameters showed that the Δ*H*° of simple ring opening by methanol correlates quite well with *T*_c_. Moreover, the propensity to polymerize relies on subtle differences in monomer structure, and a detailed geometric comparison of single-crystal and computed structures revealed that strain introduced by *fusion* of the lactone and oxanorbornene rings is key rather than in the individual rings themselves, offering a complementary strategy to design monomers for CRM. Establishing such structure–property relations of the monomers and their *T*_c_ is key for designing polymers for circularity and will be further developed in subsequent studies.

## Data Availability

Additional spectroscopic, thermoanalytic, chromatographic, and computational data files that support the findings of this study are openly available in the Yoda data repository at 10.24416/UU01-NUW22T.

## Supporting Information Available

Experimental details, characterizations, and additional optimization tables (PDF)

## Abbreviations

ROP: ring-opening polymerization
CRM: chemical recycling to monomer
DA: Diels–Alder
rDA: retro-Diels–Alder
GPC: gel permeation chromatography
DFT: density functional theory
MDSC: modulated differential scanning calorimetry
TGA: thermal gravimetric analysis
DTG: derivative thermogravimetry
