# Supplementary material for: Closed-Loop Chemical Recycling of a Biobased Poly(oxanorbornene-fused γ-butyrolactone)
Source: J Am Chem Soc. 2024 Dec 3;146(50):34628–37. doi: 10.1021/jacs.4c12678 (PMC11664497; doi:10.1021/jacs.4c12678)
Supplement: Supplementary file 1 — ja4c12678_si_001.pdf [file ja4c12678_si_001.pdf]

Electronic Supporting Information for

Closed-loop chemical recycling of a biobased poly(oxanorbornene-fused γ-butyrolactone)

Eva Harsevoort,[a,‡] Răzvan C. Cioc,[a,‡] Martin Lutz,[b] Arnaud Thevenon,[a,*] Pieter C. A. Bruijnincx[a,*]

a. Organic Chemistry and Catalysis, Institute for Sustainable and Circular Chemistry, Faculty of Science, Utrecht University, Universiteitsweg 99, 3584 CG Utrecht, The Netherlands.

b. Structural Biochemistry, Bijvoet Centre for Biomolecular Research, Faculty of Science, Utrecht University, Universiteitsweg 99, 3584 CG Utrecht, The Netherlands.

‡ Equal first authorship contribution.

* Corresponding Authors. Email: a.a.thevenon-kozub@uu.nl; p.c.a.bruijnincx@uu.nl.

General considerations 2
- Materials, reagents, and methods 2
- Spectroscopic characterizations 2
- Molecular weight measurements 2
- Thermal analysis 3

Monomer synthesis 3

Polymerization of M1 4
- General polymerization procedure 4
- Polymerization results 4
- Molecular weight analysis 5
- Effect of stirring speed and time on formation of cyclic dimer $(M1)_2$ 6
- P(M1) molecular weight monitoring during cyclic dimer formation 7
- Polymerization profile of M1 7
- Ceiling temperature determination of M1 8

Polymerization of $M1\text{-}H_2$ 9
- General polymerization procedure 9
- Ceiling temperature determination of $M1\text{-}H_2$ 9

Post-polymerization hydrogenation 10

Synthesis of cyclic dimer $(M1)_2$ 10
- Thermal stability of P(M1) in solid and solution state 11

Depolymerization experiments 11
- Reaction profile CRM of P(M1) 12

X-ray crystal structure determinations 13

Computational details 15

Structural property comparison of the monomers (from crystal structure and calculations) 16

Characterizations and spectra 19

References 50

Associated content

CCDC 2368124-2368126 contains the supplementary crystallographic data for this paper. Additional spectroscopic, thermoanalytic, chromatographic, and computational data files that support the findings of this study are openly available in the Yoda data repository at https://doi.org/10.24416/UU01-NUW22T.

General considerations

Materials, reagents, and methods

Unless stated otherwise, all solvents and commercially available reagents were used as purchased. Molecular sieves (3 or 4 Å) were activated thermally prior use (250 °C, several days). Dichloromethane (DCM) was collected from an M. Braun MB-SPS 800 solvent purification system, degassed by purging with N_2, and stored over 4 Å molecular sieves. Benzyl alcohol was distilled over CaH_2. Diphenylmethanol and 2,2-diphenylethanol were dissolved in toluene over CaH_2, stirred for *ca.* 12 h, filtered, and dried *in vacuo*. Monomers were recrystallized from DCM and dried at high vacuum overnight. The Y-**1** catalyst was synthesized according to literature.[1]

Spectroscopic characterizations

Nuclear magnetic resonance (NMR) measurements were recorded on a Varian MRF 40, a Varian VNMR-S-400, or a Jeol JNM-ECZL G 400 MHz spectrometer, at 25 °C. Chemicals shifts (δ) in ^{1}H and ^{13}C{^{1}H} NMR are reported relative to TMS with the residual solvent signal,[2] expressed in ppm, and coupling constants (J) are quoted in hertz (Hz). Peak multiplicity is described as s (singlet), d (doublet), t (triplet), q (quartet), br (broad singlet) and m (multiplet) or combinations thereof. Monomer conversion is determined from the ratio of the integrals of well resolved resonances in NMR for the polymer, monomer, and when applicable, cyclic dimer. More specifically, the conversion of **M1** is determined using the relative integrals of the signals at 6.54 ppm (corresponding to the olefinic hydrogens of the monomer) and the signals at 6.39 ppm (corresponding to the olefinic hydrogens of the repeat unit in the polymer). The conversion of **M1-H$_2$** was determined from the relative integrals at 4.88 ppm (bridgehead hydrogen of the monomer) and 4.62 ppm (bridgehead hydrogen of the polymer).

Attenuated Total Reflection Fourier Transform Infrared spectroscopy (ATR-FTIR) spectra were recorded on a PerkinElmer Frontier FTIR spectrometer. Prior to each measurement a background spectrum consisting of 8 scans was collected and subsequently subtracted from the sample spectrum. Intensities are reported with w (weak), m (middle), s (strong), b (broad) and combinations thereof. The sample was placed onto the sample plate, ensuring total coverage of the ATR crystal, and was pressed by the toner arm.

Molecular weight measurements

Gel Permeation Chromatography (GPC) measurements were carried out on a variable setup by Shimadzu (SCI-40 controller module, DGU-203 degassing unit, SIL-40 autosampler, CTO-40C column oven, RID-20A refractive index detector) equipped with an Agilent Technologies *PLgel 5µm Guard 50 x 7.5 mm* guard column and two Agilent Technologies *PLgel 5µm MIXED-D 300 x 7.7 mm* columns. *N*,*N*-dimethylformamide (peptide synthesis grade) was used as the eluent. The measurements were run at a flow rate of 1 mL/min at 65 °C at pressures between 85-90 bar and analyzed with Shimadzu *LabSolutions GPC* software. For calibration, a combined calibration set of polystyrene was used consisting of peak molecular weight (M_p) defined standards of *ca.* 4k, 7k, 13k, 20k, 50k, 100k, and 300k g mol^{-1}, which was measured with each sample batch. For isolated polymers, the samples were dissolved (1 mg/mL) in the eluent and filtered through a 0.45 µm syringe filter into the GPC vials. For crude aliquots, the complete sample was dissolved in 1 mL solvent and filtered.

Thermal analysis

Thermogravimetric analysis (TGA) measurements were recorded on a TAInstruments TGA Q50 Thermographic Analyzer under nitrogen on a platinum pan and analyzed using TA instruments *Universal Analysis 2000*. The thermal degradation was investigated with approximately 5 mg sample loaded onto the pan at a heating rate of 10 °C/min from 20 °C to 250 °C [for P(**M1**)] or 20 °C to 350 °C [for P(**M2-H$_2$**)]. The onset decomposition temperature (T_{onset}) was determined from the intersection of baseline and the tangent at max. gradient.

Modulated Differential Scanning Calorimetry (MDSC) measurements were recorded on a TA instruments Discovery DSC and analyzed within the TA instruments *Trios* software (version 5.1.1). Tzero low mass aluminum pans fitted with a Tzero aluminum lid were loaded with approximately 5 mg of sample. The modulation was set to 1 °C/min. For P(**M1**) the sample is heated with a first heating rate of 10 °C/min to 80 °C and kept at an isothermal for 5 min. Then, after cooling (3 °C/min) to 0 °C and an isothermal of 5 min, the glass transition temperatures (T_g) were determined from the second heating scan (3 °C/min) to max. 150 °C. For P(**M1-H$_2$**), the sample is heated with a first heating rate of 10 °C/min to 170 °C and kept at an isothermal for 2 min. Then, after cooling (3 °C/min) to 0 °C and an isothermal of 5 min, the glass transition temperatures (T_g) were determined from the second heating scan (3 °C/min) to max. 210 °C.

Monomer synthesis

Monomer **M1**: The hemiacetal intermediate **H1** was synthesized according to the literature procedure (Scheme S1).[3] Oxidation of the **H1** to monomer **M1** was performed either with the procedure reported previously, or with oxidation procedure reported below. **M1** was recrystallized from DCM prior to polymerization. *Oxidation procedure:* Hemiacetal **H1** (3.08 g, 20 mmol, 1 equiv.) was dissolved in DCM (30 mL). TEMPO (312 mg, 2 mmol, 10 mol%) was added and the solution was placed on a cold-water bath prior to the addition of bis(acetoxy)iodobenzene (BAIB) (6.45 g, 20 mmol, 1 equiv.). The resulting suspension was stirred at ambient temperature overnight; the solids dissolved within an hour. The mixture was concentrated *in vacuo* and the lactone was purified by silicagel flash chromatography (100 v/v% petroleum ether to 66 v/v% petroleum ether in ethyl acetate to 100 v/v% ethyl acetate) to afford the product as an off-white solid (2.5 g, 16.4 mmol, 82%).

Monomer **M1-H$_2$** has been reported previously, synthesized by oxidation of the saturated hemiacetal **H1-H$_2$** with Shvo's catalyst (Route 1, Scheme S1). In addition to the reported route, **M1-H$_2$** could also be obtained by hydrogenation of **M1** with Pd/C in 98% isolated yield (Route 2, Scheme S1). *Procedure:* **M1** (325 mg, 2.13 mmol, 1 equiv.) was dissolved in ethyl acetate (7 mL) and the solution was degassed with N_2 for 10 min. Pd/C (10 wt.%) was added (124 mg, 0.117 mmol Pd, 5.5 mol%), a hydrogen balloon was attached, and the reaction mixture was stirred vigorously at rt for 4 h. Then, the reaction mixture was filtered over celite, concentrated *in vacuo*, yielding a white solid (325 mg, 2.11 mmol, 98% yield). ^{1}H NMR ($CDCl_3$) analysis is consistent with literature.[3] **^{1}H NMR** (400 MHz, $CDCl_3$) δ 4.88 (td, J = 4.6, 1.6 Hz, 1H), 4.44 (t, J = 8.7 Hz, 1H), 3.93 (t, J = 9.0 Hz, 1H), 2.83 – 2.71 (m, 1H), 2.13 – 1.94 (m, 2H), 1.94 – 1.84 (m, 2H), 1.84 – 1.72 (m, 1H), 1.72 – 1.58 (m, 1H).

Monomer **M2** was synthesized according to literature.[4]

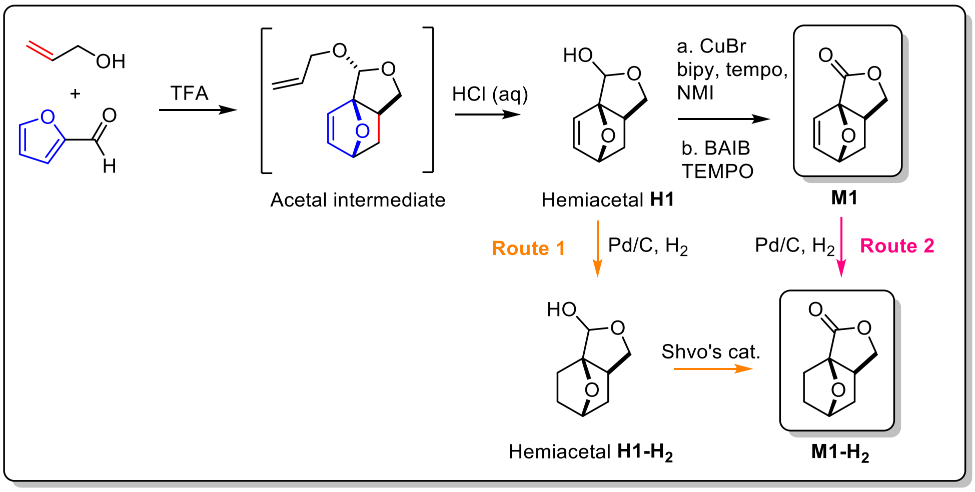

Scheme S1: Previously reported synthesis of monomer **M1** and **M1-H$_2$**, with the exception of route 2 and oxidation procedure b.

Polymerization of M1

General polymerization procedure

Polymerizations were performed in a N_2 glovebox unless mentioned otherwise. A catalyst stock solution was prepared by weighing *ca.* 10-20 mg of catalyst and dissolving it in DCM (concentration 0.025 M). Similarly, a solution of the alcohol initiator was prepared (concentration 0.2 M). To a vial, 0.4 mL of the catalyst solution (containing 0.01 mmol catalyst, 1 mol%) and 0.1 mL of the alcohol solution (0.02 mmol, 2 mol%) were added and this mixture was stirred for *ca.* 5-10 min. For ratios different than **M**/cat/I = 100/1/2, the catalyst/alcohol mixture was diluted to the amounts indicated in the polymerization tables. The monomer (152.2 mg, 1 mmol, 1 equiv.) was dissolved in 0.3 mL DCM and rapidly added to the catalyst/alcohol solution, under stirring at the indicated stirring speed, at ambient temperature. The monomer vial was rinsed with DCM (0.2 mL) and the rinse was added to the bulk reaction mixture ([**M1**]$_0$ = 1 M). The reaction mixture typically is a light-yellow solution. After the indicated time, the mixture was removed from the glovebox and quenched by adding *ca*. five drops of AcOH. An aliquot was taken for NMR analysis in $CDCl_3$ to determine the monomer conversion. The quenched mixture was concentrated, redissolved in a minimal amount of DCM, and precipitated from methanol (10 mL) while stirring, leading to the formation of a white suspension. The suspension was stirred for 30-60 min and then filtered, or decanted. The polymer was washed with methanol (2x 2 mL) and dried under vacuum, producing a white powder. The M_n was determined by GPC and end-group analysis from the ^{1}H NMR spectrum of the isolated polymer in $CDCl_3$, DMSO-d$_6$ or CD_2Cl_2, avoiding overlap of residual NMR solvent with the respective end-group signals.

Polymerization results

Table S1: Results of ROP of **M1**[a]. Entry 1-11 were reported in Table 1, here supplemented by entry S1-S5

Entry	Cat.	Init.	[M]/[Cat.]/[I]	Time (h)	M1 conv. (%)[b]	Isol. yield (%)	$M_{n,calc}$ (kg/mol) [c]	$M_{n,NMR}$ (kg/mol) [d]	$M_{n,GPC}$ (kg/mol) [e]	Đ[f]	T_{onset} (°C) [g]	T_g (°C) [h]
**S1**	La-**1**	^{i}PrOH	100/1/1	0.25	82	61	12.5	17.0	31.7	1.47	165.0	123.5
**1**	La-**1**	^{i}PrOH	100/1/2	0.25	80	51	6.1	12.6	24.8	1.42	165.4	122.8
**S2**	La-**1**	^{i}PrOH	400/1/2	0.25	82	45	25.0	28.7	53.4	1.35	169.4	124.3
**2**[i]	La-**1**	BnOH	100/1/2	0.25	80	61	6.0	10.1	22.3	1.36	166.3	120.3
**3**	La-**1**	BnOH	100/1/3	0.25	83	55	4.2	8.6	19.3	1.31	166.7	120.3
**4**[j]	La-**1**	BnOH	100/1/2	0.25	84	60	6.5	12.4	24.9	1.46	166.8	122.9
**S3**[k]	La-**1**	BnOH	100/1/2	1	83	63	6.4	12.6	24.0	1.47	165.9	122.6
**5**	La-**1**	BnOH	200/1/2	0.25	78	63	11.9	16.0	30.4	1.32	166.2	119.7
**6**	La-**1**	BnOH	400/1/2	0.25	80	68	24.4	34.1	47.3	1.34	168.8	122.3

**7**	La-**1**	BnOH	600/1/2	0.25	79	70	36.2	43.8	55.6	1.25	166.9	122.3
**8**	La-**1**	BnOH	1000/1/2	0.25	76	58	57.9	59.0	76.8	1.19	166.9	122.5
**S4**	La-**1**	Ph_2CHOH	100/1/2	0.25	81	58	6.3	16.7	22.4	1.65[l]	166.1	122.7
**S5**[m]	La-**1**	Ph_3COH	100/1/2	2	47	38	3.7	46.0	-	-	-	-
**9**	Y-**1**	^{i}PrOH	100/1/1	0.25	83	74	12.7	15.4	29.8	1.25	167.2	121.1
**10**	Y-**1**	^{i}PrOH	400/1/1	0.25 (7)	8 (82)[n]	69	50.0	34.6	47.5	1.36	165.4	123.8
**11**	Y-**1**	BnOH	100/1/1	0.25	81	64	12.4	17.4	29.4	1.27	164.9	123.1

[a] Conditions: **M1** (152 mg 1.0 mmol), Initiator (I): isopropanol (^{i}PrOH), benzyl alcohol (BnOH), diphenylmethanol (Ph_2CHOH) or triphenylmethanol (Ph_3COH), rt, stirring at 50 rpm. [b] Monomer conversion determined by ^{1}H NMR analysis. [c] $M_{n,calc}$ = conversion × [**M1**]/[Init] × MW of monomer + MW of initiator. For the polymerizations in this table, selectivity to P(**M1**) was >99%. [d] $M_{n,NMR}$ = chain length n based on end-group analysis × MW monomer + MW initiator. [e] $M_{n,GPC}$ and $M_{w,GPC}$ determined by gel permeation chromatography at 65 °C in *N,N*-dimethylformamide, calibrated with polystyrene standards. [f] Đ = M_w/M_n. [g] Onset decomposition temperature (T_{onset}) measured by thermal gravimetric analysis (TGA), determined from the intersection of baseline and the tangent at max. gradient. [h] Glass transition temperature (T_g) measured by modulated differential scanning calorimetry (MDSC) from the second heating scan with a heating rate of 3 °C/min. [i] Average result of five experiments, see **Error! Reference source not found.** for standard deviations. [j] Performed with $[\mathbf{M1}]_0$ = 2 M in DCM. [k] Performed at 2.3 g monomer scale. [l] Bimodal GPC trace. [m] Stirring speed 1000 rpm. [n] Conversion after 7 h.

Table S2: Reproducibility of ROP of **M1** (Table 1, entry 2[a]).

**Entry**	**M1 conv. (%)**	**Sel. to P(M1) (%)**	**Isol. P(M1) yield (%)** [b]	**$M_{n,calc}$ (kg/mol)** [c]	**$M_{n,NMR}$ (kg/mol)** [d]	**$M_{n,GPC}$ (kg/mol)** [e]	**Đ**[f]	**T_{onset} (°C)** [g]	**T_g (°C)** [h]
**2-a**	81	99	64	6.140	10.237	22.734	1.377	166.7	121.5
**2-b**	78	100	66	5.988	10.889	23.426	1.361	-	-
**2-c**	81	98	66	6.064	8.908	20.777	1.338	163.8	120.5
**2-d**	78	100	53	5.988	10.439	21.625	1.380	167.7	120.3
**2-e**	79	99	56	5.988	10.063	22.987	1.362	167.0	119.3
**Avg.**	79	99	61	6.034	10.107	22.310	1.363	166.3	120.4
**St. Dev.**	1.5	1.0	5.4	0.061	0.660	0.970	0.015	1.5	0.8

[a] Conditions: **M1** (152 mg 1.0 mmol), **M1**/La(HMDS)$_3$/BnOH = 100/1/2, $[\mathbf{M1}]_0$ = 1 M in DCM, rt, 15 min, stirring at 50 rpm. [b] Monomer conversion determined by ^{1}H NMR analysis. [c] $M_{n,calc}$ = conversion × [**M1**]/[Init] × MW of monomer + MW of initiator. [d] $M_{n,NMR}$ = chain length n based on end-group analysis × MW monomer + MW initiator. [e] $M_{n,GPC}$ and $M_{w,GPC}$ determined by gel permeation chromatography at 65 °C in N,N-dimethylformamide, calibrated with polystyrene standards. [f] Đ = M_w/M_n. [g] Onset decomposition temperature (T_{onset}) measured by thermal gravimetric analysis (TGA), determined from the intersection of baseline and the tangent at max. gradient. [h] Glass transition temperature (T_g) measured by modulated differential scanning calorimetry (MDSC) from the second heating scan with a heating rate of 3 °C/min.

Molecular weight analysis

$M_{n,calc}$ was calculated with the assumption of all equivalents of ROH initiating polymerization. The discrepancy between $M_{n,calc}$ arises from the limitations associated with the GPC measurements and calibration against polystyrene. $M_{n,calc}$ and $M_{n,GPC}$ correlate well, particularly within catalyst/initiator system (see Figure S1, left, for La-**1** catalyzed polymerizations). Furthermore, $M_{n,GPC}$ and $M_{n,NMR}$ correlate well, see Figure S1, right.

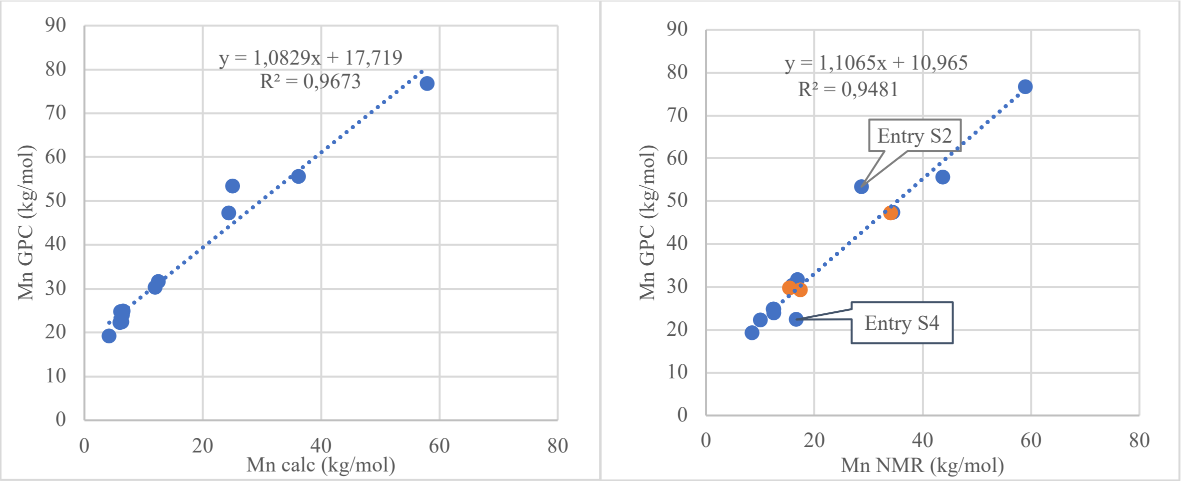

Figure S1: Left: $M_{n\,GPC}$ vs $M_{n\,calc}$, of La-**1** catalyzed experiments (in blue), from Table S1. Right: The $M_{n\,NMR}$ and $M_{n\,GPC}$ data from Table S1 show a linear correlation with an offset of ca 11 kg/mol. Orange data points: Y-**1** catalyzed polymerization. Blue data points: La-**1** catalyzed polymerizations. The outliers entry S2 (**M1**/La-**1**/^{i}PrOH = 400/1/2) and entry S4 (**M1**/La-**1**/Ph_2CHOH = 100/1/2) are labeled.

Effect of stirring speed and time on formation of cyclic dimer (M1)$_2$

It was observed that stirring rate and reaction time influence cyclic dimer formation and therefore maximal polymer yield. When the reaction mixture was stirred at 1000 rpm for 2 h, the cyclic dimer was formed in 14% spectroscopic yield, as determined by quantitative ^{1}H NMR (Table S3, entry S7). Dimer formation could be avoided by either decreasing the reaction time (entry S8) or the stirring speed (entry S6). Combining both a lower stirring speed and reaction time resulted in only 2% cyclic dimer formation (entry 2). A decrease in cyclic dimer formation and increase in polymer yield was also observed when using 3 equiv. of BnOH (entry 3 vs S9), or 2 equiv. of Ph_2CHOH (entry S4 vs S10).

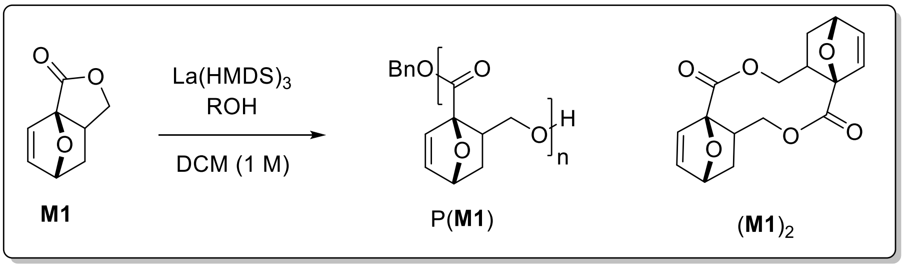

Table S3: Results of ROP of **M1** with different stirring speeds, reaction times and initiators.[a]

Entry	Init.	[M1]/[Cat.]/[I]	Time (h)	Stir speed (RPM)	M1 conv. (%)[b]	Sel. P(M1) (%)	Isol. P(M1) yield (%)	$M_{n,calc}$ (kg/mol) [c]	$M_{n,NMR}$ (kg/mol) [d]	$M_{n,GPC}$ (kg/mol) [e]	Đ[f]
S6	BnOH	100/1/2	2	50	83	94	57	6.0	10.3	17.8	1.36
S7	BnOH	100/1/2	2	1000	76	82	34	4.7	5.5	15.9	1.30
2	BnOH	100/1/2	0.25	50	80	99	61	6.0	10.1	22.3	1.36
S8	BnOH	100/1/2	0.25	1000	81	99	43	6.2	11.5	15.4	1.41
3	BnOH	100/1/3	0.25	50	83	99	55	4.2	8.6	19.3	1.31
S9	BnOH	100/1/3	2	1000	81	86	37	3.6	6.8	16.2	1.21
S4	Ph_2CHOH	100/1/2	0.25	50	81	>99	58	6.3	14.3	22.4	1.65
S10	Ph_2CHOH	100/1/2	2	1000	78	90	57	5.4	6.4	17.1	1.33

[a] Conditions: **M1** (152 mg 1.0 mmol), La(HMDS)$_3$ catalyst, [**M1**]$_0$ = 1 M in DCM, rt. [b] Monomer conversion determined by ^{1}H NMR analysis. [c] $M_{n,calc}$ = conversion × [**M1**]/[Init.] × MW of monomer + MW of initiator. [d] $M_{n,NMR}$ = chain length n based on end-group analysis × MW monomer + MW initiator. [e] $M_{n,GPC}$ and $M_{w,GPC}$ determined by gel permeation chromatography at 65 °C in *N*,*N*-dimethylformamide, calibrated with polystyrene standards. [f] Đ = M_w/M_n.

P(M1) molecular weight monitoring during cyclic dimer formation

La(HMDS)$_3$ catalyzed

M1 was polymerized according to the general polymerization procedure with **M1**/La(HMDS)$_3$/BnOH = 100/1/2. After 15 min stirring at 50 rpm, an aliquot was taken, quenched with AcOH and evaporated. The same polymerization mixture was stirring for 2 h at 1000 rpm and another aliquot was taken and quenched with AcOH, and after 5 h at 1000 rpm the remaining reaction mixture was quenched. The aliquots were analyzed with ^{1}H NMR in $CDCl_3$ to determine the ratio **M1** : P(**M1**) : (**M1**)$_2$ and with GPC to determine the molecular weight and dispersity. For the ^{1}H NMR spectra and GPC traces, see the data repository.

Table S4: Product distribution and molecular weight data during cyclic dimer formation with La-**1**.

**Entry**	**Reaction time**	**Ratio M1 : P(M1) : (M1)$_2$**	**$M_{n,GPC}$ (kg/mol)**	**Đ**
1	15 min	15 : 84 : 1	23.8	1.69
2	2 h	11 : 83 : 6	19.0	1.35
3	5 h	10 : 75 : 15	15.9	1.31

Y-1 catalyzed

M1 was polymerized according to the general polymerization procedure with **M1**/Y-1/BnOH = 100/1/2. After 15 min stirring at 50 rpm, an aliquot was taken, quenched with AcOH and evaporated. The same polymerization mixture was stirring for 2 h at 1000 rpm and another aliquot was taken and quenched with AcOH, and after 24 h at 1000 rpm the remaining reaction mixture was quenched. The aliquots were analyzed with ^{1}H NMR in $CDCl_3$ to determine the ratio **M1** : P(**M1**) : (**M1**)$_2$ and with GPC to determine the molecular weight and dispersity. For the ^{1}H NMR spectra and GPC traces, see the data repository.

Table S5: Product distribution and molecular weight data during cyclic dimer formation with **Y-1**.

**Entry**	**Reaction time**	**Ratio M1 : P(M1) : (M1)$_2$**	**$M_{n,GPC}$ (kg/mol)**	**Đ**
1	15 min	20 : 78 : 2	21.2	1.35
2	2 h	16 : 82 : 3	21.0	1.36
3	24 h	14 : 81 : 5	17.1	1.36

Polymerization profile of M1

Monomer conversion and polymer molecular weight were followed over time for **M1**/La/BnOH 100/1/2, at 0.5 M in DCM at 0 °C.

Procedure: A catalyst stock solution was prepared by weighing La(HMDS)$_3$ (12.6 mg, 0.020 mmol, 1 mol%). The catalyst was dissolved in dry DCM (0.8 mL, concentration of 0.025 M). A BnOH solution was prepared (13.7 mg in 0.633 mL dry DCM, concentration 0.2 M). A portion of the BnOH solution (0.2 mL, 0.04 mmol, 2 mol%) was then added to the La(HMDS)$_3$ solution and the mixture was stirred for a few minutes and then cooled to 0 °C. A solution of monomer **M1** (311 mg, 2.05 mmol, 1 equiv.) solution in dry DCM (3.1 mL) at 0 °C was then added at once to the catalyst/alcohol mixture, resulting in a final volume of 4.1 mL ([**M1**]$_0$ = 0.5 M). The reaction was stirred at 0 °C and aliquots of *ca.* 0.5 mL were taken at the indicated times and quenched with a few drops of AcOH. The conversion was determined with ^{1}H NMR ($CDCl_3$). The aliquots were precipitated from MeOH (2 mL), filtered, and washed with MeOH (*ca.* 1 mL). The polymers were dissolved in DMF and analyzed by GPC. For the

^{1}H NMR spectra and GPC traces, see the data repository. No (**M1**)$_2$ formation was observed, the selectivity to P(**M1**) was >99%.

Table S6: Conversion of **M1** and P(**M1**) molecular weight data over time.

Time (s)	M1 conversion (%)	$M_{n,GPC}$ (kg/mol)	Đ
32	29.1	14.9	1.08
62	43.8	18.5	1.09
90	54.3	20.1	1.11
122	62.1	21.3	1.12
180	70.5	22.3	1.13
302	77.5	22.5	1.14
600	80.7	23.1	1.13
3480	80.6	23.5	1.13

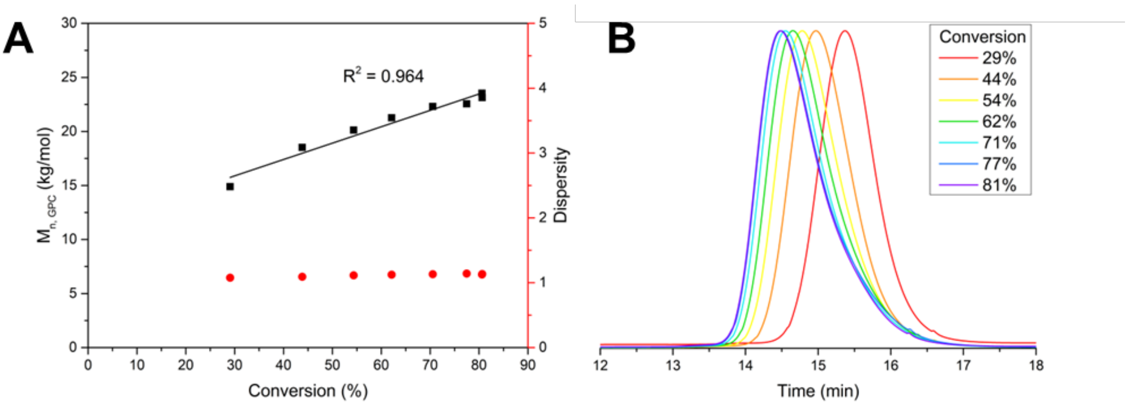

Figure S2: (**A**) Plot of $M_{n,GPC}$ (black) and Đ (red) of P(**M1**) obtained with **M1**/La-**1**/BnOH = 100/1/2, (0 °C, 0.5 M) as a function of conversion; (**B**) Overlay of the GPC traces at various conversions.

Ceiling temperature determination of M1

In the glovebox, a catalyst stock solution was prepared by weighing La(HMDS)$_3$ and dissolving it in dry DCM (concentration 0.05 M). Separately, a BnOH solution was prepared (concentration 0.2 M) as well as a solution of **M1** (179 mg) in dry DCM (2 mL). Outside of the glovebox (using Schlenk techniques) 0.2 mL of the catalyst solution (containing 0.01 mmol catalyst, 1 mol%) and 0.1 mL of the alcohol solution (0.02 mmol, 2 mol%) were mixed and stirred for 5-10 min. The vial was brought to the desired temperature (0 °C, 10 °C, 20 °C and 30 °C resp.) and 1.7 mL of the monomer solution (corresponding to 152 mg of **M1**, 1 mmol, 1 equiv.) was added rapidly to the catalyst/alcohol solution (final volume 2 mL, [**M1**]$_0$ = 0.5 M). The conversion of the monomer was monitored by ^{1}H NMR by periodic sampling (quenching with few drops AcOH) until **M1** conversion was constant. For the ^{1}H NMR spectra, we refer to the data repository.

The equilibrium conversion and resulting equilibrium monomer concentration [**M1**]$_{eq}$ at the different temperatures are shown in Table S7. The Van 't Hoff plot (Figure 3) gives a straight line with a slope of –3.108 and an intercept of 8.970. According to ln[**M1**]$_{eq}$ = $\Delta H_p°/RT - \Delta S_p°/R$, $\Delta H_p°$ is calculated to be –25.8 kJ/mol, and $\Delta S_p°$ to be –74.6 J/mol*K. According to $T_c = \Delta H_p° /(\Delta S_p° + R \ln [\mathbf{M1}]_0)$, the T_c is calculated to be 73 °C at [**M1**]$_0$ = 1 mol/L.

Table S7: The equilibrium concentrations and conversion of **M1** at different temperatures.

T (°C)	Equilibrium conversion of M1 (%)	$[M1]_{eq}$ (mol/L)	$ln[M1]_{eq}$	1/T ×10^3 (K^{-1})
0	81	0.095	-2.354	3.661
10	75	0.125	-2.079	3.532
20	62	0.190	-1.661	3.411
30	42	0.290	-1.238	3.299

Polymerization of M1-H_2

General polymerization procedure

In the glovebox, a stock solution of BnOH (0.1 M in DCM) was prepared and added to a weighed amount of $La(HMDS)_3$, forming a catalyst/initiator mixture with $La(HMDS)_3$ (0.05 M) and BnOH (0.1 M) in dry DCM. A solution of **M1-H_2** of 2.14 M in dry DCM was prepared. Outside of the glovebox both vials were cooled to the desired temperature, and (using Schlenk techniques) a volume of the catalyst/initiator mixture containing 1 mol% $La(HMDS)_3$ and 2 mol% BnOH with respect to **M1-H_2** was added rapidly to the monomer solution (final concentration [**M1-H_2**]$_0$ = 1.5 M). The conversion of the monomer was monitored by ^{1}H NMR by periodic sampling (quenching with few drops AcOH) until **M1-H_2** conversion was constant. For the ^{1}H NMR spectra, we refer to the data repository. P(**M1-H_2**) formed at -40 °C was isolated and analyzed (see Table S8).

Table S8: Results for ROP of **M1-H_2** according to the general procedure.

T (°C)	Time (h)	M1-H_2 conv. (%)[b]	Isol. yield (%)	$M_{n,calc}$ (kg/mol) [c]	$M_{n,NMR}$ (kg/mol) [d]	$M_{n,GPC}$ (kg/mol) [e]	Đ[f]	T_{onset} (°C) [g]	T_g (°C) [h]
-40	67	75	47%	5.8	47.3	48.7	2.71	263.5	136.8

Ceiling temperature determination of M1-H_2

The equilibrium conversion and resulting equilibrium monomer concentration [**M1-H_2**]$_{eq}$ at the different temperatures are shown in Table S9. The datapoint obtained at –10 °C was omitted for obtaining the thermodynamic parameters for ROP of **M1-H_2**, since the equation ln[**M1-H_2**]$_{eq}$ = $\Delta H_p°/RT - \Delta S_p°/R$ is derived using Flory's assumption, that the reactivity of an active center at a sufficiently long macromolecule does not depend on its polymerization degree.[5] Shorter chains (n < 20) do not conform to Flory's assumption, and 7% conversion of monomer was expected to form oligomers <20.

The Van 't Hoff plot (Figure 3) gives a straight line with a slope of –2.985 and an intercept of 11.822. According to ln[**M1-H_2**]$_{eq}$ = $\Delta H_p°/RT - \Delta S_p°/R$, $\Delta H_p°$ is calculated to be –24.8 kJ/mol, and $\Delta S_p°$ to be –-98.3 J/mol*K. According to $T_c = \Delta H_p° /(\Delta S_p° + R \ln$ [**M1-H_2**]$_0$), the T_c is calculated to be –21 °C at [**M1-H_2**]$_0$ = 1 mol/L.

Table S9: The equilibrium concentrations and conversion of **M1-H_2** at different temperatures.

T (°C)	Equilibrium conversion of M1-H_2 (%)	[M1-H_2]$_{eq}$ (mol/L)	ln[M1-H_2]$_{eq}$	1/T ×10^3 (K^{-1})
-10 (omitted)	*7*	*1.395*	*0.333*	*3.80*
-20	29	1.065	0.063	3.95
-25	47	0.810	-0.229	4.03
-30	60	0.600	-0.511	4.11

-35	66	0.510	-0.673	4.20
-40	75	0.375	-0.981	4.29

Post-polymerization hydrogenation

For hydrogenation, ^{i}PrOH end-capped P(**M1**) was used to avoid the potential deprotection of the BnOH end-group by hydrogenolysis.

Procedure: P(**M1**) (106 mg, 0.70 mmol) formed by Y-**1** (Table 1, entry 9) was dissolved in DCM (3 mL) and the solution was degassed with N_2. Pd/C (10 wt.% Pd) (70 mg, 0.07 mmol, 10 mol% Pd) was added and a hydrogen balloon was attached. After stirring at rt for 6 h, the mixture was filtered over celite, and the filter was washed with DCM (2x 10 mL). The filtrate was concentrated *in vacuo* and dried at 60 °C under vacuum, affording a white powder. ^{1}H NMR analysis showed full conversion of the olefinic bond.

Table S10: Comparison of the polymer properties of P(**M1**) and P(**M1-H$_2$**)

	**P(M1)**	**P(M1-H$_2$)**
**Synthesis**	Table 1, entry 9, formed with M1/Y-1/iPrOH 100/1/1	Post polymerization hydrogenation of P(**M1**) from Table 1, entry 9
$M_{n,NMR}$	15376 g mol^{-1}	17876 g mol^{-1}
$M_{n\ GPC}$	29779 g mol^{-1}	31578 g mol^{-1}
**Đ**	1.25	1.19
$T_{5\%}$ **(°C)**	162.1	261.2 °C
T_g **(°C)**	121.1 °C	129.6 °C

Synthesis of cyclic dimer (M1)$_2$

Procedure: 1.063 mL of a 0.025 M stock solution of La(HMDS)$_3$ in DCM (containing 0.027 mmol, 1 mol%) and a 0.266 mL of a 0.2 M stock solution of BnOH in DCM (containing 0.053 mmol, 2 mol%) were stirred for 5 minutes. A solution of the **M1** (404 mg, 2.66 mmol, 1 equiv.) in 0.797 mL DCM was rapidly added to the catalyst/initiator solution. The vial containing **M1** was rinsed with 0.55 mL DCM, resulting in a final concentration of 1 M. The mixture was stirred at 1500 rpm for 4 days and quenched with *ca*. 20 drops of AcOH. ^{1}H NMR analysis show a mixture of four compounds: the unreacted **M1** (13%), the polymer P(**M1**) (7%), the cyclic dimer (**M1**)$_2$ (68%), and a third, similar, unknown product (12%). The mixture was separated by silicagel flash column chromatography (30 to 60 v/v % ethyl acetate in petroleum ether), affording the cyclic dimer (**M1**)$_2$ (155 mg, 0.509 mmol, 38% yield) as an off-white solid.

TLC (ethyl acetate/petroleum ether, 50/50 v/v) R_f = 0.25; **^{1}H NMR** (400 MHz, $CDCl_3$) δ 6.50 – 6.40 (m, 4H), 5.04 (d, *J* = 4.7 Hz, 2H), 4.43 (t, *J* = 11.9 Hz, 2H), 4.28 (dd, *J* = 11.6, 4.3 Hz, 2H), 2.74 (ddt, *J* = 11.8, 7.4, 3.7 Hz, 2H), 1.65 – 1.54 (m, 2H), 1.37 (dt, *J* = 11.5, 4.2 Hz, 2H); **^{13}C{^{1}H} NMR** (100 MHz, $CDCl_3$) δ 169.2, 137.2, 134.4, 87.7, 77.4, 66.7, 39.7, 30.2; **IR** (ATR): $\tilde{\nu}$ = 3096 (w), 3006 (w), 1737 (m), 2957 (s), 2922 (m), 2852 (m), 1743 (s), 1731 (s), 1458 (w), 1403 (w), 1385 (w), 1309 (w), 1356 (w), 1330 (m), 1293 (m), 1276 (m), 1261 (m), 1200 (w), 1177 (w), 1138 (m), 1084 (m), 1055 (m), 1015 (w), 957 (m), 932 (m), 864 (m), 785 (w), 726 (m), 711 (m) cm^{-1}; **ESI-MS**: *m/z* [*M*+Na]$^+$ calculated for $C_{16}H_{16}NaO_6$: 327.0845, found: 327.0831. Also *m/z* [2*M*+Na]$^+$ was observed, calculated for $C_{32}H_{32}NaO_{12}$: 631.1792, found: 631.1811.

Thermal stability of P(M1) in solid and solution state

Solid state

A solid sample of P(**M1**) (4.2 mg) formed with **M1**/La-1/BnOH = 100/1/2 (Table 1 entry S3) was heated for MDSC from 0 °C to 130 °C with 3.00 °C/min (well below the T_{onset} measured by TGA). ^{1}H NMR analysis of the P(**M1**) sample after heating showed 5% rDA (see Figure S50).

Solution state

A sample of P(**M1**) (12 mg) formed according general polymerization procedure with **M1**/Y-**1**/BnOH = 100/1/2 (with $M_{n,GPC}$ = 21.7 kg mol^{-1} and Đ = 1.31, see GPC trace in Figure S52) was dissolved in 1 mL DMSO-d_6 and an initial NMR spectrum was measured (t = 0 h). The sample was heated to 100 °C for 1 h and measured again. NMR analysis indicated 2% allyl furoate formation, a decreased molecular weight and increased dispersity of the polymer ($M_{n,GPC}$ 12.5 kg mol^{-1}; Đ = 1.37, see Figure S53), due to chain scission caused by the rDA pathway. From ^{1}H NMR analysis (see Figure S51, it was observed 2% rDA had taken place.

Depolymerization experiments

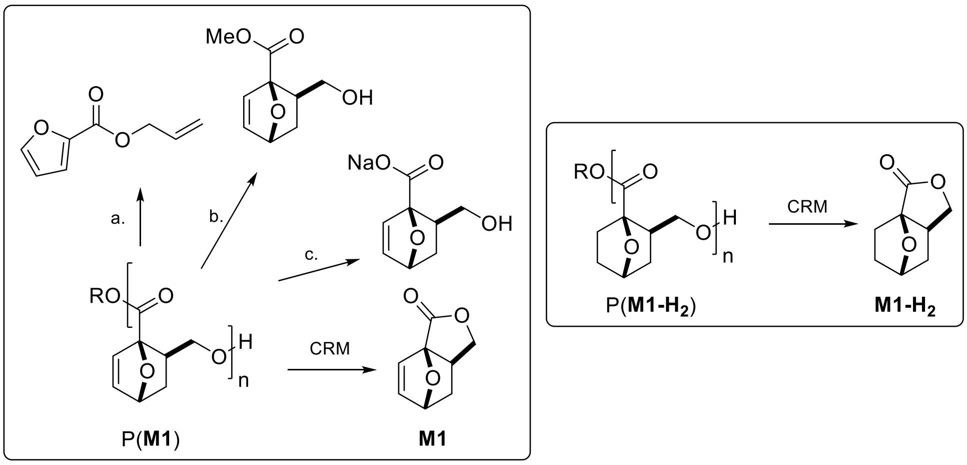

Scheme S2: Overview of the possible recycling routes for P(**M1**) and P(**M1-H$_2$**).

^{1}H NMR spectra and GPC traces are reported in the data repository.

Procedure CRM: The polymer P(**M1**) or P(**M1-H$_2$)** was dissolved in DCM (0.2 M), *p*TsOH (10 mol%) was added, and the solution was stirred at 100 °C in a capped microwave vial. After the indicated time, the reaction was quenched with a few drops of Et_3N.

For P(**M1**): Starting polymer, formed with Y-**1** and BnOH (conditions of Table 1 entry 11) (M_n = 23.1 kg mol^{-1}, Đ = 1.26); 78% conversion after 1 h (M_n residual polymer = 15.3 kg/mol, Đ = 1.32), 81% conversion after 6 h (M_n residual polymer = 9.0 kg mol^{-1}, Đ = 1.26).

Procedure a: P(**M1**) (15 mg) was dissolved in DMSO-d_6 (1 mL). The sample was transferred to an NMR tube and heated at 150 °C for 1 h. Full and clean conversion to allyl furoate was observed. Spectra data was consistent with reported values.[6]

Procedure b: P(**M1**) (38 mg) was dissolved in DCM (0.5 mL), and the solution was diluted with methanol (0.5 mL). Solid K_2CO_3 (1 mg, 2.5 wt.% w.r.t. to the polymer) was added and the solution was stirred at rt for 2 h. NMR analysis indicated complete and clean methanolysis of the polymer. Spectral data was consistent with reported values.[3]

Procedure c: P(**M1**) (10 mg) was added to a freshly prepared NaOH 1 M solution in D_2O and the formed suspension was stirred at rt for 18 h. Gradual dissolution was observed (complete within the experiment timeframe). NMR analysis indicated complete and clean saponification of the polymer. Spectral data was consistent with reported values.[3]

Reaction profile CRM of P(M1)

The general procedure was followed for CRM of P(**M1**) from Table 1 entry S3, using 10 mol% *p*TsOH. Aliquots were taken, quenched with Et_3N, and analyzed with ^1H NMR (see Figure S3) and GPC (see data repository).

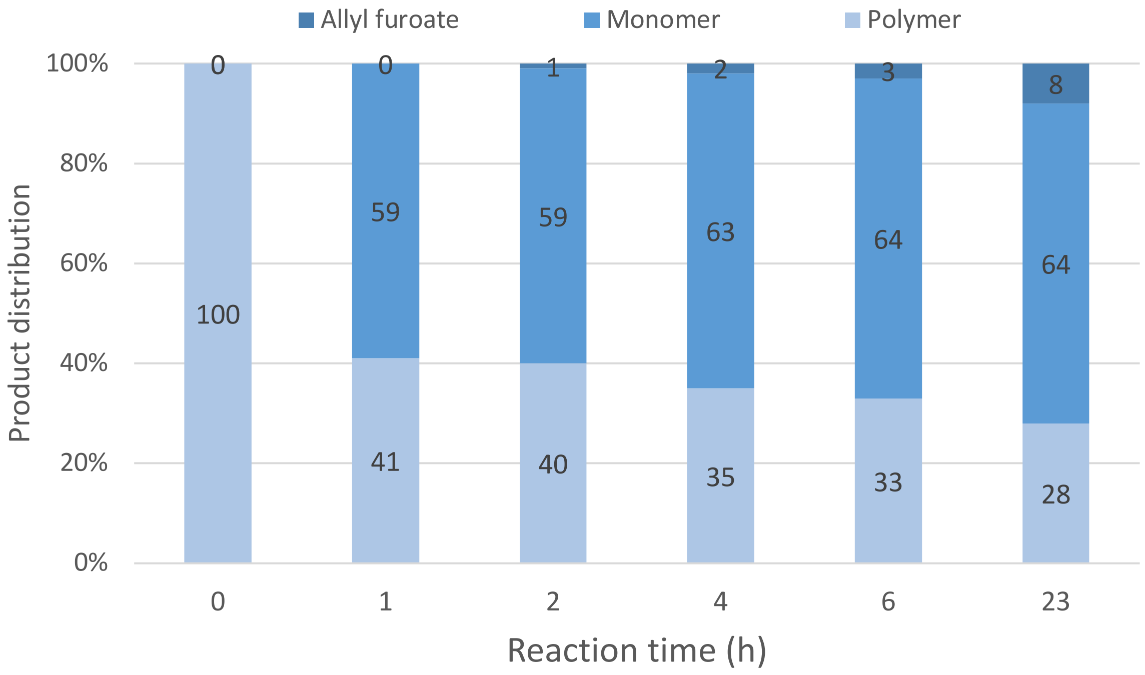

Figure S3: The product distribution during the depolymerization reaction of P(**M1**) (Table 1 entry S3) with *p*TsOH.

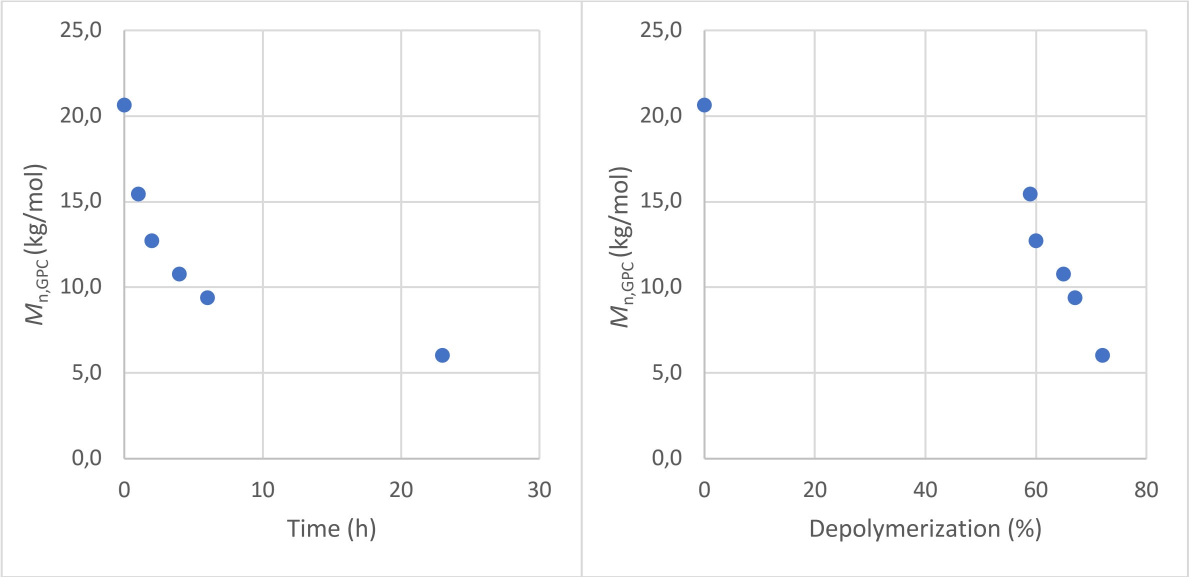

Figure S4: Left: $M_{n,GPC}$ decrease over time during CRM of P(**M1**). Right: $M_{n,GPC}$ decrease with depolymerization percentage.

X-ray crystal structure determinations

Monomer M1

Single crystals of **M1** were grown by cooling (at *ca.* –25 °C) of a methanolic solution.

$C_8H_8O_3$, Fw = 152.14, colorless plate, 0.40 × 0.17 × 0.04 mm^3, monoclinic, Ia (no. 9), a = 8.8258(9), b = 6.8625(6), c = 11.3344(10) Å, β = 99.108(6)°, V = 677.84(11) Å^3, Z = 4, D_x = 1.491 g/cm^3, μ = 0.12 mm^{-1}. The diffraction experiment was performed on a Bruker Kappa ApexII diffractometer with sealed tube and Triumph monochromator (λ = 0.71073 Å) at a temperature of 150(2) K up to a resolution of (sin θ/λ)$_{max}$ = 0.65 Å^{-1}. The Eval15 software[7] was used for the intensity integration. A numerical absorption correction and scaling was performed with SADABS[8] (correction range 0.92-1.00). A total of 5509 reflections was measured, 1558 reflections were unique (R_{int} = 0.026), 1426 reflections were observed [I>2σ(I)]. The structure was solved with Patterson superposition methods using SHELXT.[9] Structure refinement was performed with SHELXL-2018[10] on F^2 of all reflections. Non-hydrogen atoms were refined freely with anisotropic displacement parameters. All hydrogen atoms were located in difference Fourier maps. Hydrogen atoms H2 and H3 were refined freely with isotropic displacement parameters; all other hydrogen atoms were refined with a riding model. 108 Parameters were refined with 2 restraints (floating origin). R1/wR2 [I > 2σ(I)]: 0.0305 / 0.0646. R1/wR2 [all refl.]: 0.0350 / 0.0666. S = 1.078. The absolute structure of this light-atom compound could not be determined reliably from anomalous scattering. Residual electron density between -0.20 and 0.18 e/Å^3. Geometry calculations and checking for higher symmetry was performed with the PLATON program.[11]

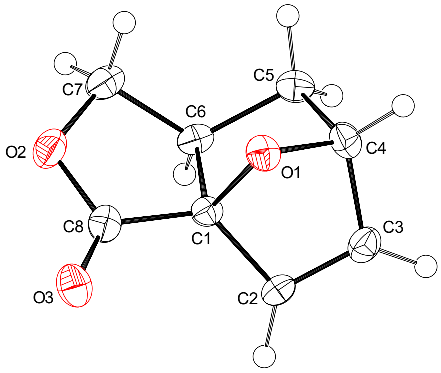

Figure S5: Molecular structure of **M1** in the crystal (50% probability level).

Monomer M1-H_2

Single crystals of saturated monomer were obtained by slow evaporation of an ethyl acetate solution.

$C_8H_{10}O_3$, Fw = 154.16, colorless block, 0.38 × 0.34 × 0.27 mm^3, orthorhombic, $P2_12_12_1$ (no. 19), a = 5.6095(2), b = 6.4739(2), c = 19.5029(10) Å, V = 708.26(5) Å^3, Z = 4, D_x = 1.446 g/cm^3, μ = 0.11 mm^{-1}. The diffraction experiment was performed on a Bruker Kappa ApexII diffractometer with sealed tube and Triumph monochromator (λ = 0.71073 Å) at a temperature of 150(2) K up to a resolution of (sin θ/λ)$_{max}$ = 0.65 Å^{-1}. The Eval15 software[7]]was used for the intensity integration. A numerical absorption correction and scaling was performed with SADABS[8] (correction range 0.92-1.00). A total of 8587 reflections was measured, 1641 reflections were unique (R_{int} = 0.022), 1593 reflections were observed [I>2σ(I)]. The structure was solved with Patterson superposition methods using SHELXT.[9] Structure refinement was performed with SHELXL-2018[10] on F^2 of all reflections. Non-hydrogen atoms were refined freely with anisotropic displacement parameters. All hydrogen atoms were located in difference Fourier maps and refined with a riding model. 100 Parameters were refined with no restraints. R1/wR2 [I > 2σ(I)]: 0.0285 / 0.0773. R1/wR2 [all refl.]: 0.0298 / 0.0783. S = 1.075. The absolute structure of this light-atom compound could not be determined reliably from anomalous scattering. Residual

electron density between -0.21 and 0.24 e/Å^3. Geometry calculations and checking for higher symmetry was performed with the PLATON program.[11]

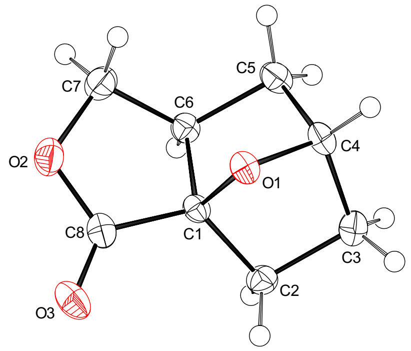

Figure S6: Molecular structure of **M1-H₂** in the crystal (50% probability level).

Cyclic dimer (M1)₂

Suitable crystals for X-ray crystal structure determination were grown by cooling (at *ca.* –25 °C) of a solution in DCM.

$C_{16}H_{16}O_6$, Fw = 304.29, colorless block, 0.44 × 0.21 × 0.18 mm^3, orthorhombic, Pca2$_1$ (no. 29), a = 7.7933(2), b = 8.8814(2), c = 19.5145(5) Å, V = 1350.69(6) Å^3, Z = 4, D_x = 1.496 g/cm^3, μ = 0.12 mm^{-1}. The diffraction experiment was performed on a Bruker Kappa ApexII diffractometer with sealed tube and Triumph monochromator (λ = 0.71073 Å) at a temperature of 150(2) K up to a resolution of $(\sin \theta/\lambda)_{max}$ = 0.70 Å^{-1}. The Eval15 software[7] was used for the intensity integration. A multi-scan absorption correction and scaling was performed with SADABS[8] (correction range 0.72-0.75). A total of 27692 reflections was measured, 3948 reflections were unique (R_{int} = 0.034), 3737 reflections were observed [I>2σ(I)]. The structure was solved with Patterson superposition methods using SHELXT.[9] Structure refinement was performed with SHELXL-2018[10] on F^2 of all reflections. Non-hydrogen atoms were refined freely with anisotropic displacement parameters. All hydrogen atoms were located in difference Fourier maps and refined freely with isotropic displacement parameters. 263 Parameters were refined with 1 restraint (floating origin). R1/wR2 [I > 2σ(I)]: 0.0334 / 0.0871. R1/wR2 [all refl.]: 0.0351 / 0.0879. S = 1.038. The absolute structure of this light-atom compound could not be determined reliably from anomalous scattering. Residual electron density between -0.18 and 0.27 e/Å^3. Geometry calculations and checking for higher symmetry was performed with the PLATON program.[11]

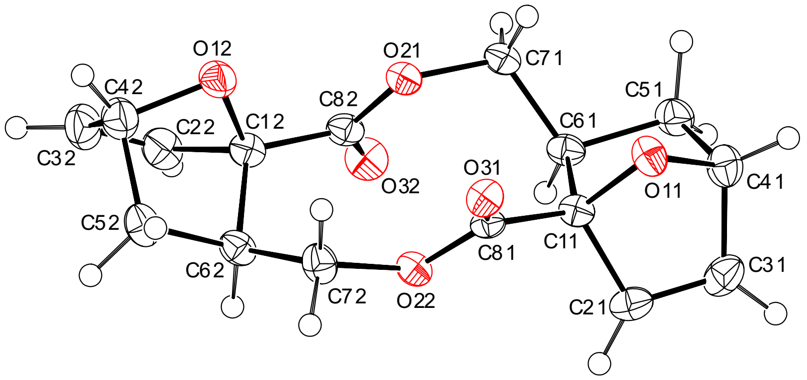

Figure S7: Molecular structure of (**M1**)₂ in the crystal (50% probability level).

CCDC 2368124-2368126 contain the supplementary crystallographic data for this paper. These data can be obtained free of charge from The Cambridge Crystallographic Data Centre via www.ccdc.cam.ac.uk/data_request/cif.

The crystal structure determination for **M2** was reported previously (CCDC 2006649).[4]

Computational details

Density functional theory (DFT) calculations were performed using the B3LYP functional with all electron 6-311+G(d,p) basis set on all atoms as implemented in Gaussian 16 C.01 program.[12] Grimme's D3 correction scheme with Becke-Johnson damping was used in all calculations.[13] Nature of the stationary points was confirmed by the vibrational analysis carried out at the same level of theory. All structures corresponding to local minima showed no imaginary frequencies. Reaction Gibbs free energies (ΔG_{298K}) were computed using the results of the normal-mode analysis within the ideal gas approximation at a pressure of 1 atm and temperature of 298.15 K.

Coordinates of optimized structures are presented in the log files, which can be found in the data repository, as well as the input files.

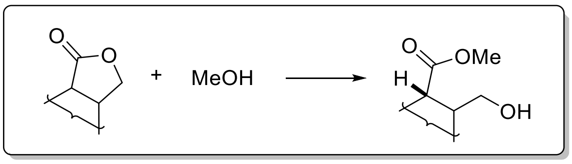

Table S11: Computed energies of lactone ring-opening with methanol.

Structure	Energies lactone monomers		Energies methanol adducts		Energy of ring opening reaction		Experimental T_c (°C)
	H (a.u.)	G (a.u.)	H (a.u.)	G (a.u.)	ΔH (kcal mol^{-1})	ΔG (kcal mol^{-1})	
**MeOH**	-115.712698	-115.73978	n.a.	n.a.	n.a.	n.a.	n.a.
**GBL**	-306.496151	-306.530871	-422.219864	-422.267419	-6.912	2.028	−136[14]
**M1**	-535.308983	-535.350073	-651.016094	-651.067991	-12.560	-1.739	73
**M1-H$_2$**	-536.53579	-536.577789	-652.265102	-652.316898	-10.425	0.421	-21
**M2**	-535.31687	-535.357899	-651.049242	-651.099604	-12.346	-1.208	-
**3,4-T6GBL**	-462.510248	-462.552226	-578.24058	-578.292905	-11.066	-0.564	0[15]
**4,5-T6GBL**	-462.513879	-462.555823	-578.242531	-578.295504	-10.011	0.062	4[16]
**3,4-C6GBL**	-462.514107	-462.556088	-578.236116	-578.287904	-5.843	4.997	-
**Pht**	-458.948455	-458.988362	-574.668054	-574.718236	-4.330	6.216	-
**BiL**	-423.210984	-423.249801	-538.943863	-538.99252	-12.664	-1.844	106[17]
**BiL**$^{=}$	-421.997424	-422.035753	-537.733424	-537.781554	-14.6222	-3.7782	118[18]

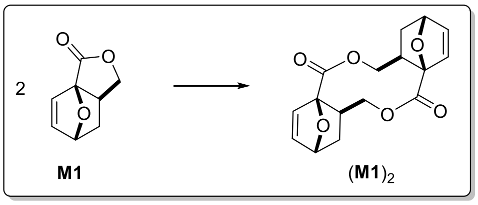

Table S12: Computed energy of the cyclic dimer (**M1**)$_2$ formation.

Energy M1		Energy (M1)$_2$		Energy of reaction, per mol of dimer		Energy of reaction, per mol of M1	
H (a.u.)	G (a.u.)	H (a.u.)	G (a.u.)	ΔH (kcal mol^{-1})	ΔG (kcal mol^{-1})	ΔH (kcal mol^{-1})	ΔG (kcal mol^{-1})
-535.308983	-535.350073	-1070.648169	-1070.708817	-18.953	-5.441	-9.476	-2.720

Structural property comparison of the monomers (from crystal structure and calculations)

Table S13: Relaxation of the DFT calculated angles around the bridgehead atoms.

Angle (°) (DFT)	M1 → M1-open (Δ)	M1-H$_2$ → M1-H$_2$-open (Δ)	M2 → M2-open (Δ)
C2-C1-C8	125.9 → 115.7 (10.2)	122.4 → 113.8 (8.6)	127.6 → 117.4 (10.2)
C5-C6-C7	118.7 → 111.5 (7.2)	118.0 → 110.9 (7.1)	118.3 → 117.3 (1.0)

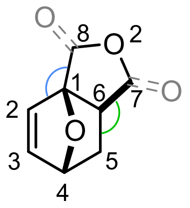

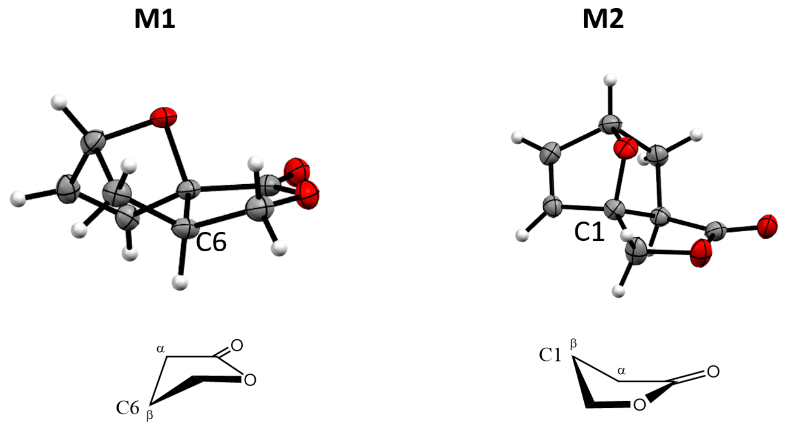

Figure S8: Envelope conformation of lactones **M1** and **M2** (from the X-ray crystal structures)

Table S14: Comparison between experimental (X-ray crystal structure) and theory (DFT) derived bond lengths (Å), angles and torsion angles (°) in the lactone fragment.[a]

Monomer	**M1**		**M1-H$_2$**		**M2**[4]		**GBL**
Method	Exp.	Theo.	Exp.	Theo.	Exp.	Theo.	Theo.
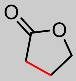 C1-C6	1.546(3)	1.5590	1.5295(19)	1.5379	1.546(2)	1.5569	1.5298
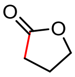 C1-C8	1.513(3)	1.5162	1.5104(19)	1.5181	1.505(2)	1.5199	1.5235
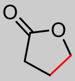 C6-C7	1.525(3)	1.5317	1.529(2)	1.5335	1.503(2)	1.5069	1.5340
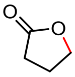 O2-C7	1.470(3)	1.4565	1.471(2)	1.4536	1.4646(18)	1.4477	1.4448
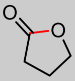 O2-C8	1.342(3)	1.3591	1.3504(19)	1.3596	1.3569(18)	1.3678	1.3636
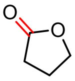 O3-C8	1.203(3)	1.1958	1.2014(19)	1.1961	1.2078(18)	1.1965	1.1969
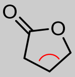 C1-C6-C7	100.77(18)	99.8	100.76(12)	100.7	104.14(12)	101.4	101.8
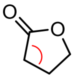 C6-C1-C8	105.02(19)	104.4	104.59(11)	104.8	100.76(11)	104.0	103.6
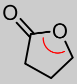 C7-O2-C8	111.66(17)	111.7	110.65(11)	111.4	111.00(11)	112.0	110.8
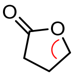 O2-C7-C6	106.03(18)	105.7	105.80(12)	105.8	103.96(12)	105.0	105.5
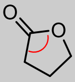 O2-C8-C1	108.50(19)	107.9	108.72(13)	108.2	110.23(12)	109.2	107.5
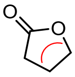 C1-C6-C7-O2	-26.1(2)	-30.5	-28.13(14)	-28.9	28.68(15)	26.1	-29.8
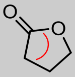 C6-C1-C8-O2	-18.9(2)	-19.9	-21.31(15)	-17.7	22.08(15)	20.7	-16.5
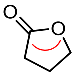 C7-O2-C8-C1	1.8(2)	-0.1	2.94(16)	-1.3	-4.63(17)	4.9	2.6
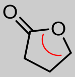 C8-O2-C7-C6	16.3(3)	20.6	16.70(16)	20.0	-15.68(17)	13.9	20.8
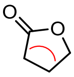 C8-C1-C6-C7	26.9(2)	30.2	29.44(14)	27.8	-30.45(14)	-28.1	27.4

[a] The atom numbering of the lactone fragments of **M1**/**M1-H$_2$** vs **M2** differs due to the regioisomery. For comparison of the structural parameters of the lactone fragment, the related/analogous parameters are compared, as depicted in the red highlighted bonds, angles and torsion angles in the table. The atom numbers are given as found in **M1** and **M1-H$_2$**.

Table S15: Comparison between experimental (X-ray crystal structure) and theory (DFT) derived bond lengths (Å) and angles (°) of oxanorbornene fragments.

**Monomer**	**M1**		**M1-H₂**		**M2[4]**		**Oxa-norbornene**
Method	Exp.	Theo.	Exp.	Theo.	Exp.	Theo.	Theo.
C1-C6	1.546(3)	1.5590	1.5295(19)	1.5379	1.546(2)	1.5569	1.5628
C1-C2	1.506(3)	1.5093	1.5180(19)	1.5240	1.506(2)	1.5156	1.5230
C2-C3	1.330(3)	1.3353	1.560(2)	1.5621	1.324(2)	1.3356	1.3338
C3-C4	1.516(3)	1.5248	1.535(2)	1.5468	1.514(2)	1.5267	1.5230
C4-C5	1.555(3)	1.5693	1.538(2)	1.5467	1.553(2)	1.5682	1.5628
C5-C6	1.545(3)	1.5464	1.550(2)	1.5510	1.5414(19)	1.5427	1.5561
O1-C1	1.447(3)	1.4413	1.4505(16)	1.4446	1.4442(17)	1.4401	1.4348
O1-C4	1.451(3)	1.4422	1.4598(17)	1.4436	1.4474(18)	1.4380	1.4348
C1-C2-C3	104.1(2)	104.4	99.87(11)	99.9	104.77(13)	104.6	105.4
C1-C6-C5	101.26(18)	101.3	101.38(11)	101.7	102.26(11)	102.1	100.8
C1-O1-C4	94.67(16)	95.3	94.90(10)	95.8	95.04(10)	95.9	96.2
C2-C1-C6	108.94(18)	109.6	113.63(11)	112.9	109.36(11)	107.8	106.8
C2-C3-C4	106.6(2)	106.0	102.16(11)	101.8	106.28(13)	106.0	105.4
C3-C4-C5	107.68(19)	108.3	111.11(12)	110.8	108.68(12)	108.4	106.8
C4-C5-C6	100.52(19)	100.3	100.96(11)	100.4	99.74(11)	99.8	100.8
O1-C1-C2	102.35(18)	102.6	103.33(10)	103.1	102.17(11)	102.1	101.9
O1-C1-C6	101.16(17)	100.3	101.77(10)	101.8	99.49(11)	100.1	101.1
O1-C4-C3	101.29(18)	101.5	101.86(10)	102.2	101.26(12)	101.4	101.9
O1-C4-C5	100.75(18)	100.3	101.78(11)	101.9	100.77(11)	100.4	101.1

Table S16: Comparison between experimental (X-ray crystal structure) and theory (DFT) angles (°) around the bridgehead carbons of the oxanorbornene-fused γ-butyrolactones.

**Monomer**	**M1**		**M1-H₂**		**M2[4]**		**(M1)₂**	
Method	Exp.	Theo.	Exp.	Theo.	Exp.	Theo.	Exp.	Theo.
C2-C1-C8	127.9(2)	125.9	122.87(12)	122.4	127.50(13)	127.6	116.14(14) for C21-C11-C81<br>114.15(15) for C22-C12-C82	117.9
C5-C6-C7	118.24(19)	118.7	116.83(12)	118.0	117.42(12)	118.3	108.65(13) for C51-C61-C71<br>107.57(14) for C52-C62-C72	116.0
O1-C1-C8	108.42(17)	111.3	108.65(11)	110.1	110.87(12)	111.4	109.60(13) for O11-C11-C81<br>114.38(14) for O12-C12-C82	114.5

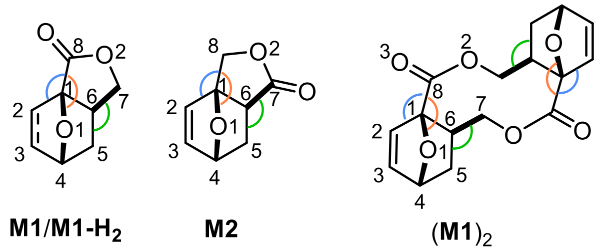

Characterizations and spectra

For two representative polymerization experiments (Table 1, entry 2 and 9), crude NMR spectra for conversion determination and NMR, IR, TGA, MDSC and GPC of the isolated polymer samples are reported. For the data of all other experiments in Table S1 we refer to the data repository.

P(M1) obtained with M1/La(HMDS)$_3$/BnOH = 100/1/2 (Table 1, entry 2)

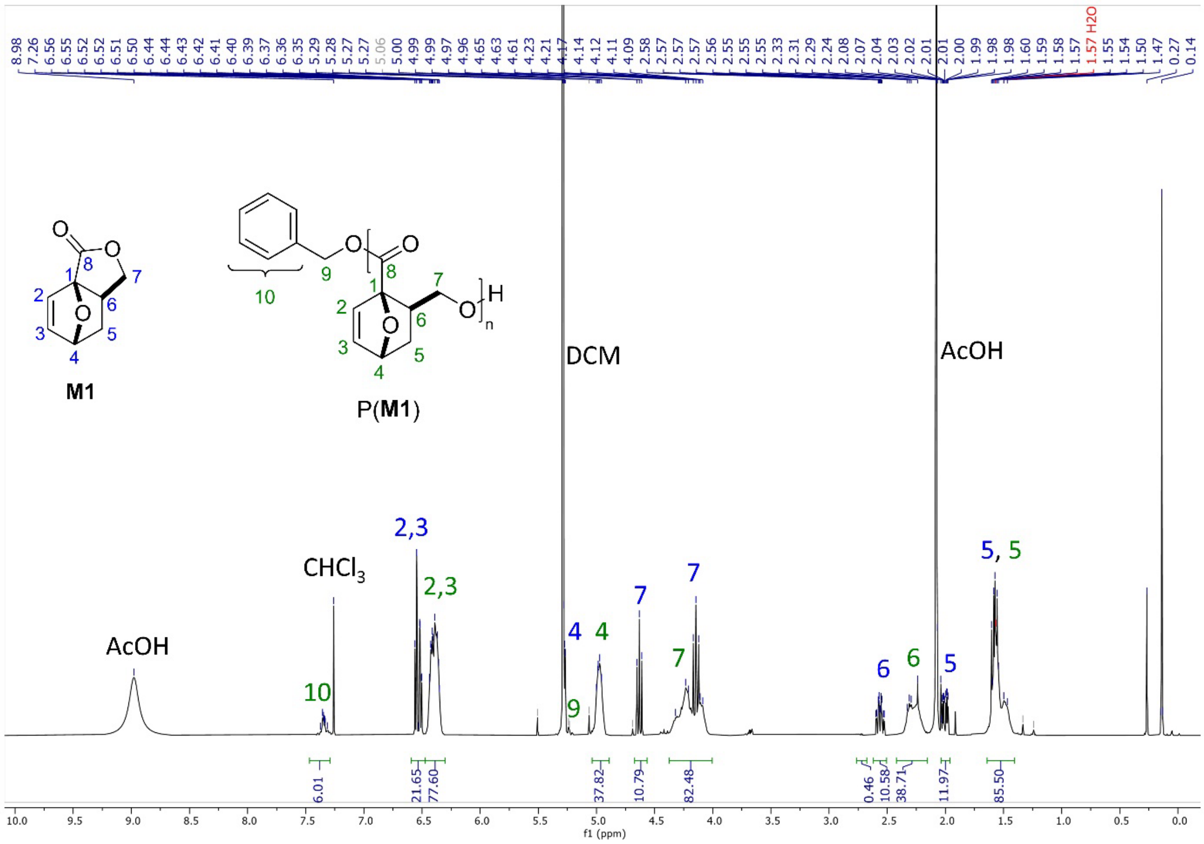

Figure S9: Crude ^{1}H NMR (CDCl$_3$) spectrum of P(**M1**) obtained with **M1**/La(HMDS)$_3$/BnOH = 100/1/2, (Table 1 entry 2); 78% conversion to polymer.

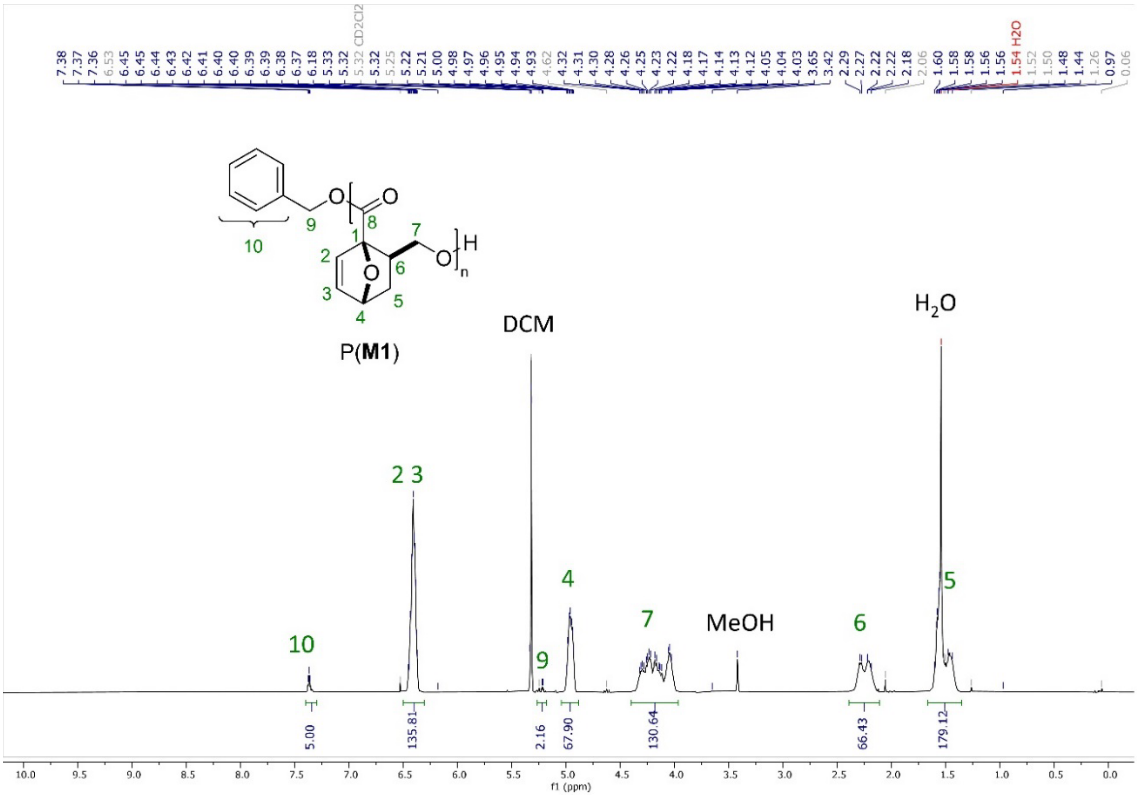

Figure S10: ^{1}H NMR (CD_2Cl_2) spectrum of isolated P(**M1**) obtained with **M1**/La(HMDS)$_3$/BnOH = 100/1/2, (Table 1, entry 2); $M_{n,NMR}$ = 10439 g mol^{-1}.

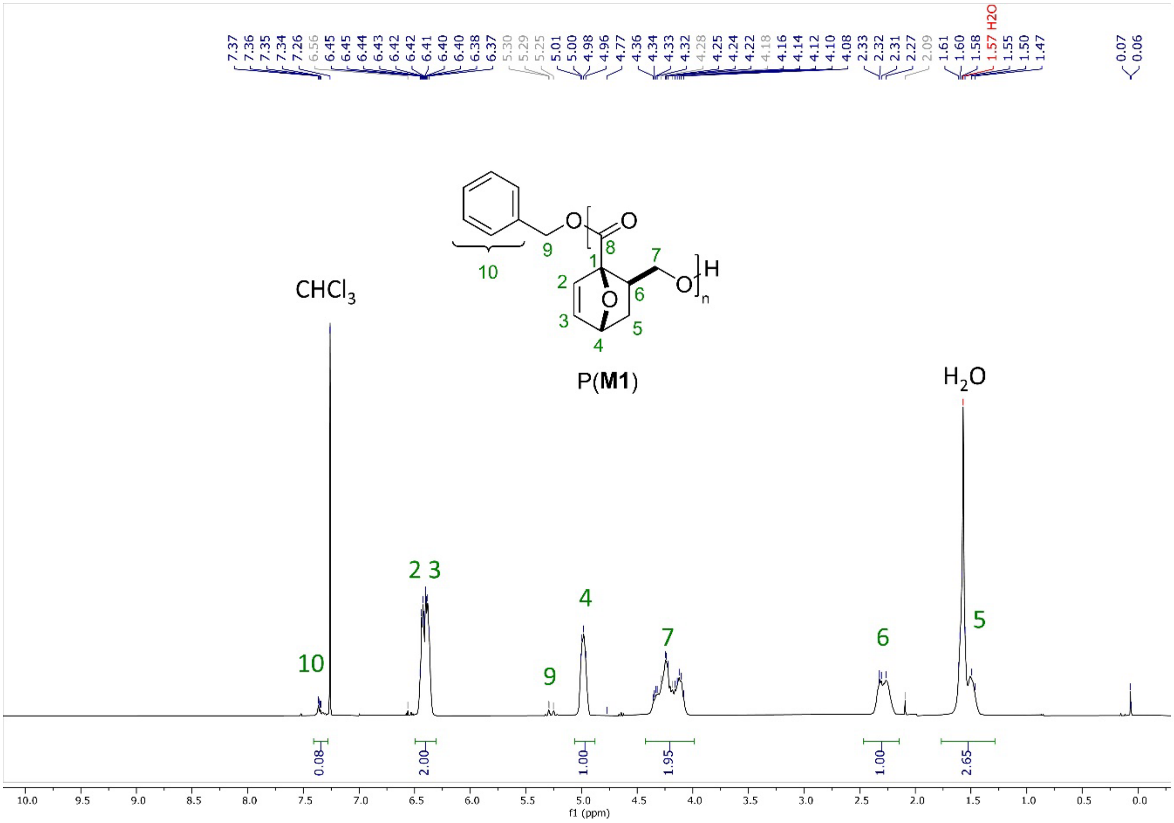

Figure S11: ^{1}H NMR ($CDCl_3$) spectrum of isolated P(**M1**) obtained with **M1**/La(HMDS)$_3$/BnOH = 100/1/2, (Table 1, entry 2).

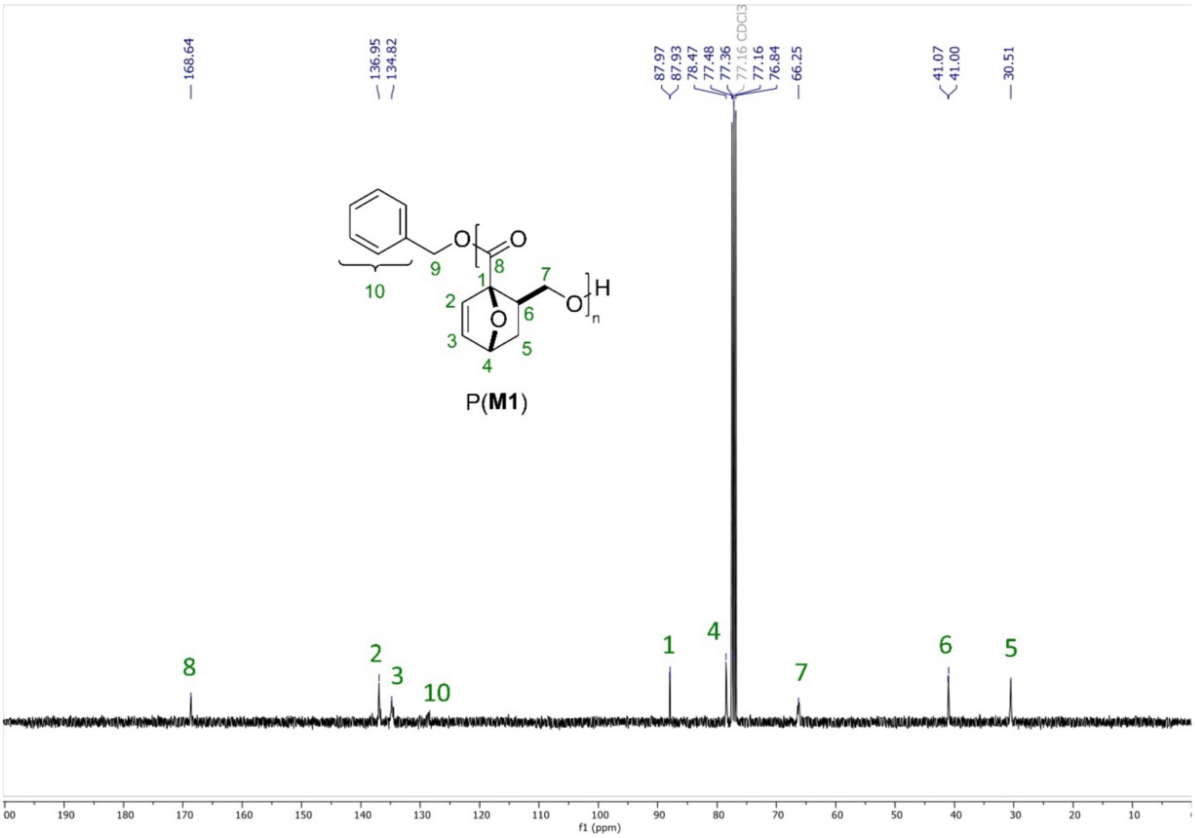

Figure S12: $^{13}C\{^1H\}$ NMR ($CDCl_3$) spectrum of isolated P(**M1**) obtained with **M1**/La(HMDS)$_3$/BnOH = 100/1/2 (Table 1, entry 2).

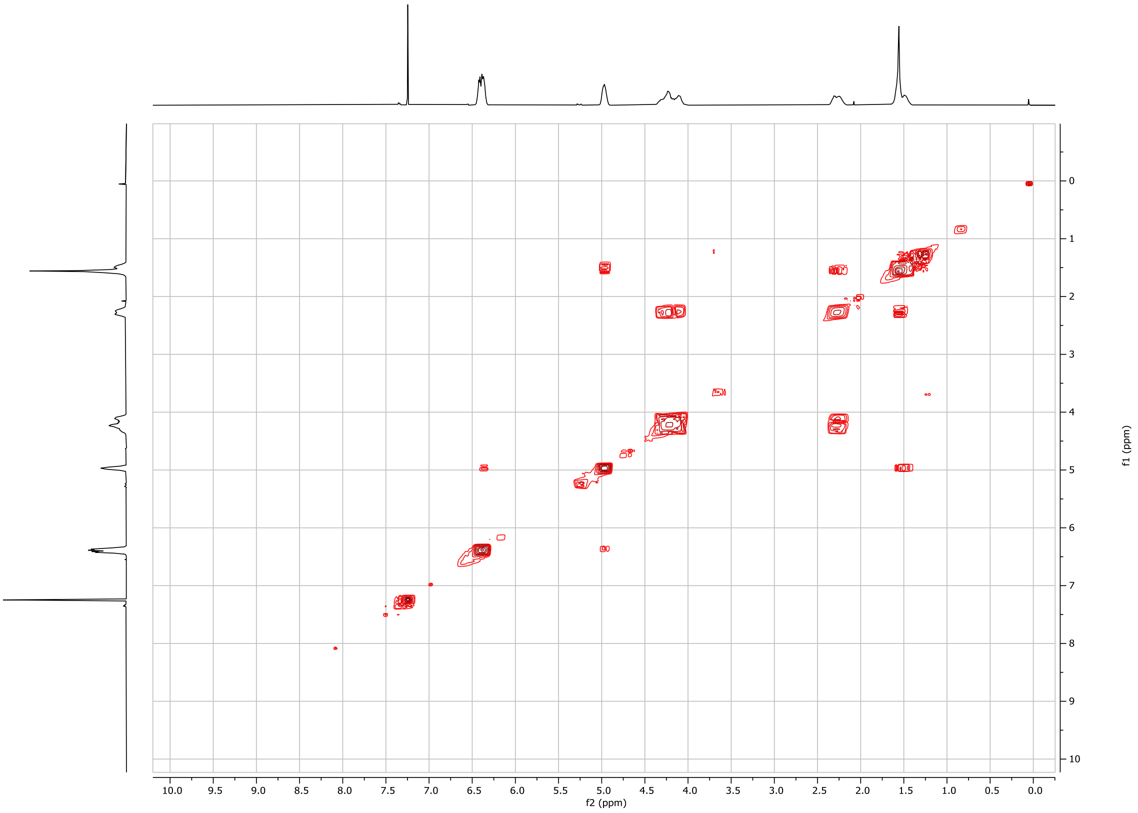

Figure S13: ^1H-^1H COSY ($CDCl_3$) spectrum of isolated P(**M1**) obtained with **M1**/La(HMDS)$_3$/BnOH = 100/1/2 (Table 1, entry 2).

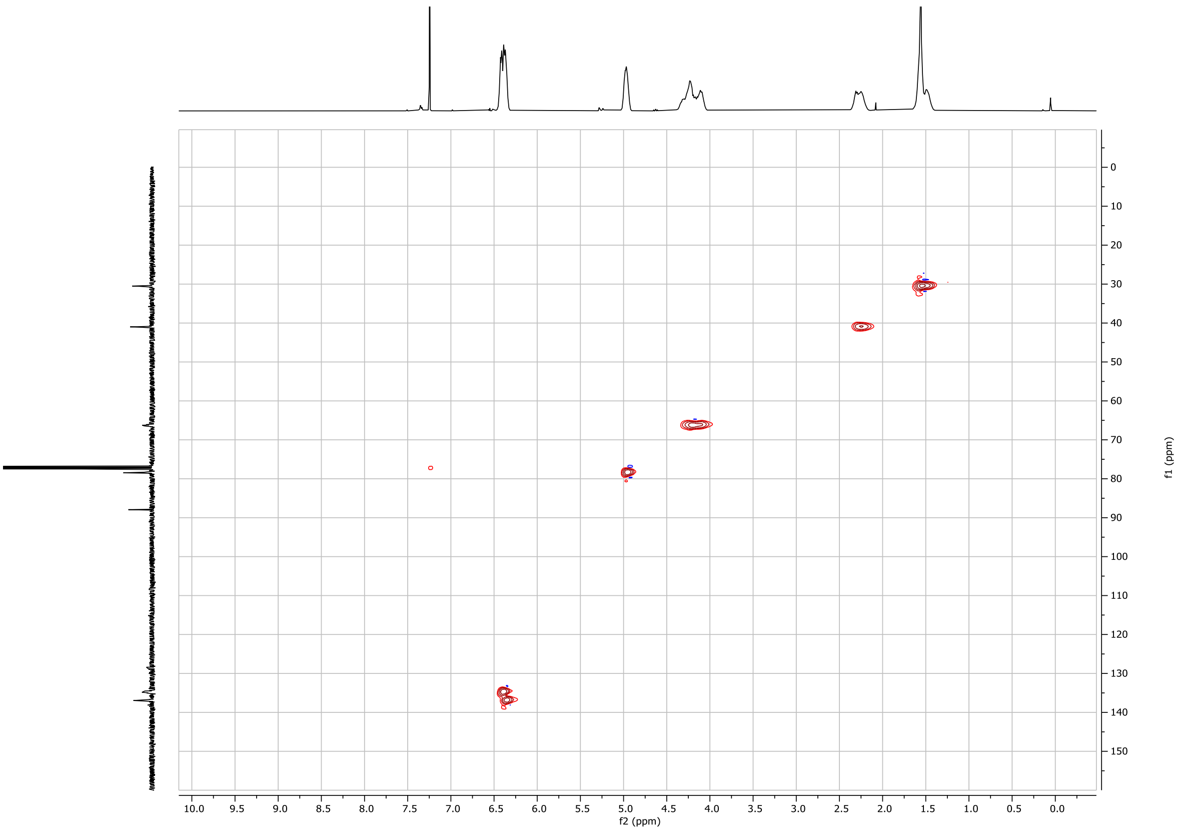

Figure S14: ASAP-HMQC ($CDCl_3$) spectrum of isolated P(**M1**) obtained with **M1**/La(HMDS)$_3$/BnOH = 100/1/2 (Table 1, entry 2).

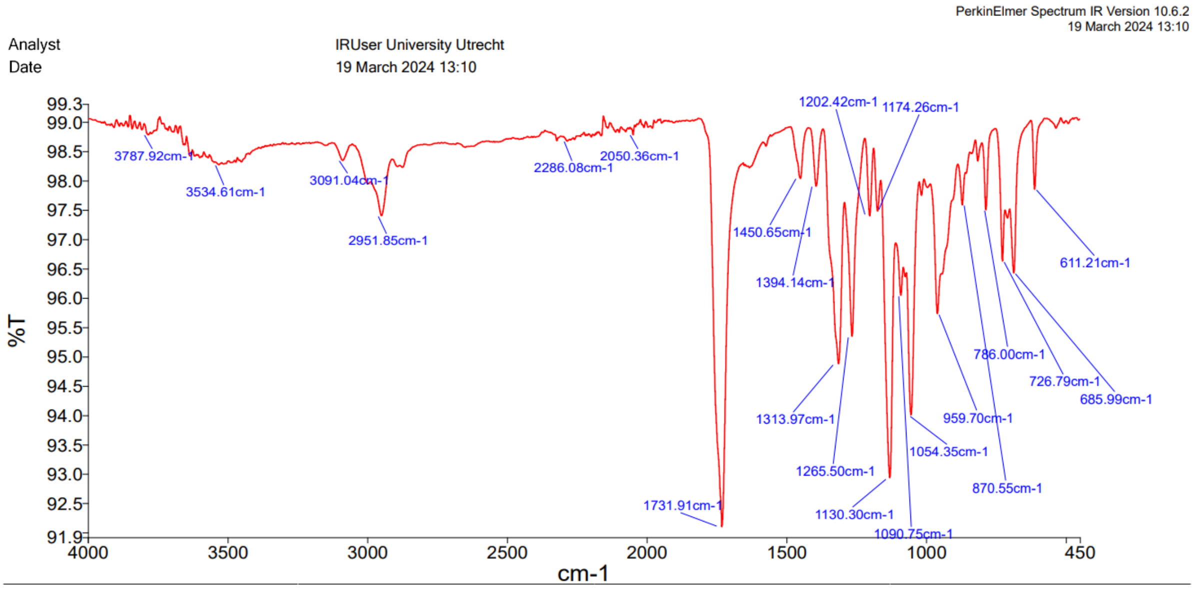

Figure S15: ATR-FTIR spectrum of isolated P(**M1**) obtained with **M1**/La(HMDS)$_3$/BnOH = 100/1/2 (Table 1, entry 2).

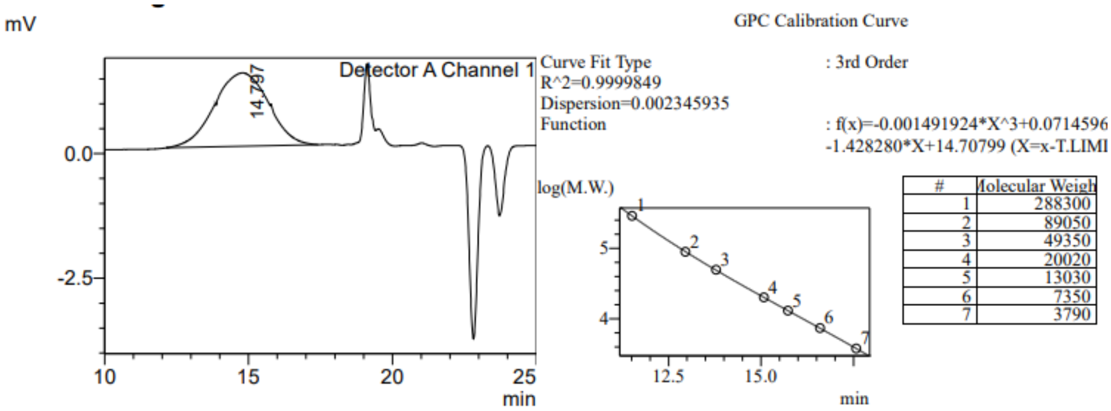

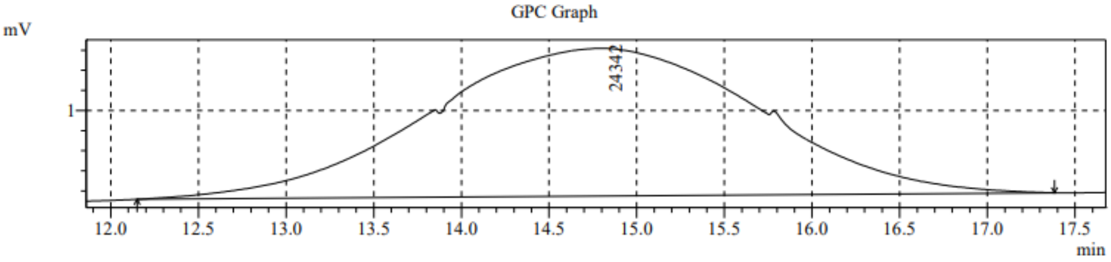

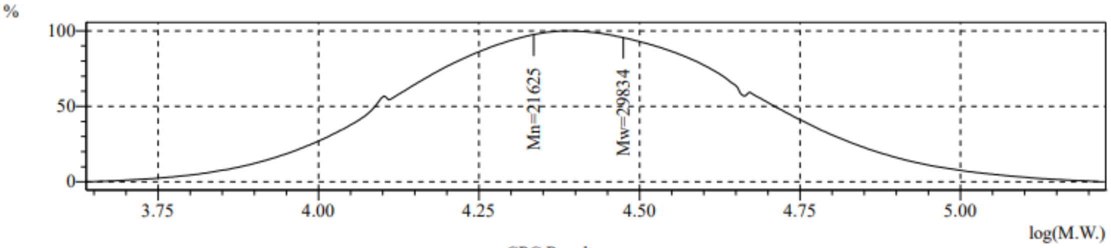

GPC Results

Peak#:1 (Detector A Channel 1)

[Peak Information]

Title	Time(min)	Molecular Weigl	Height
Start	12.150	168850	116
Top	14.797	24342	1470
End	17.383	4330	181

Area : 192109

[Average Molecular Weight]

Number Average Molecular Weight(Mn)	21625
Weight Average Molecular Weight(Mw)	29834
Mw/Mn	1.37960

Figure S16: GPC trace of isolated P(**M1**) obtained with **M1**/La(HMDS)$_3$/BnOH = 100/1/2 (Table 1, entry 2) and used calibration; $M_{n,GPC}$ = 21625 g mol^{-1}, Đ = 1.38.

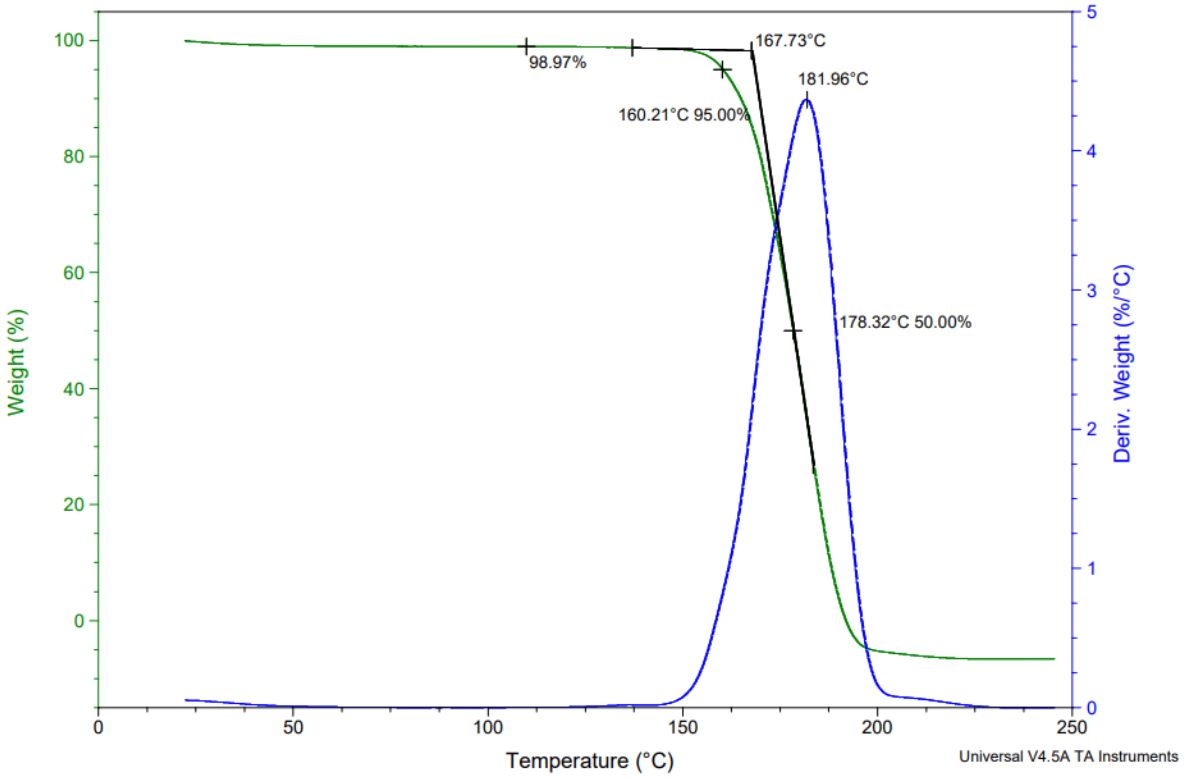

Figure S17: TGA (green) and DTG (blue) curves of P(**M1**) obtained with **M1**/La(HMDS)$_3$/BnOH = 100/1/2 (Table 1, entry 2); T_{onset} = 167.7 °C, $T_{5\%}$ = 160.2 °C, $T_{50\%}$ = 178.3 °C, T_{max} = 182.0 °C.

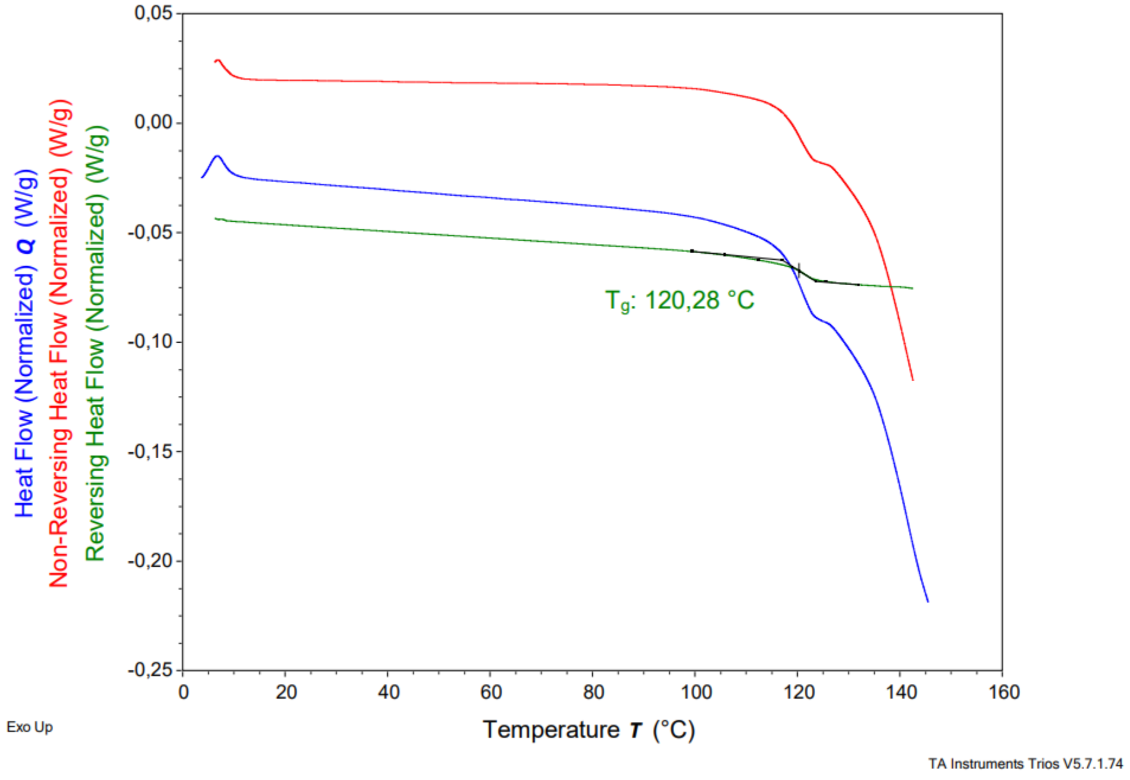

Figure S18: MDSC thermogram (second heating scan) of P(**M1**) obtained with **M1**/La(HMDS)$_3$/BnOH = 100/1/2 (Table 1, entry 2); T_g = 120.3 °C.

P(M1) obtained with M1/Y-1/iPrOH = 100/1/1 (Table 1, entry 9)

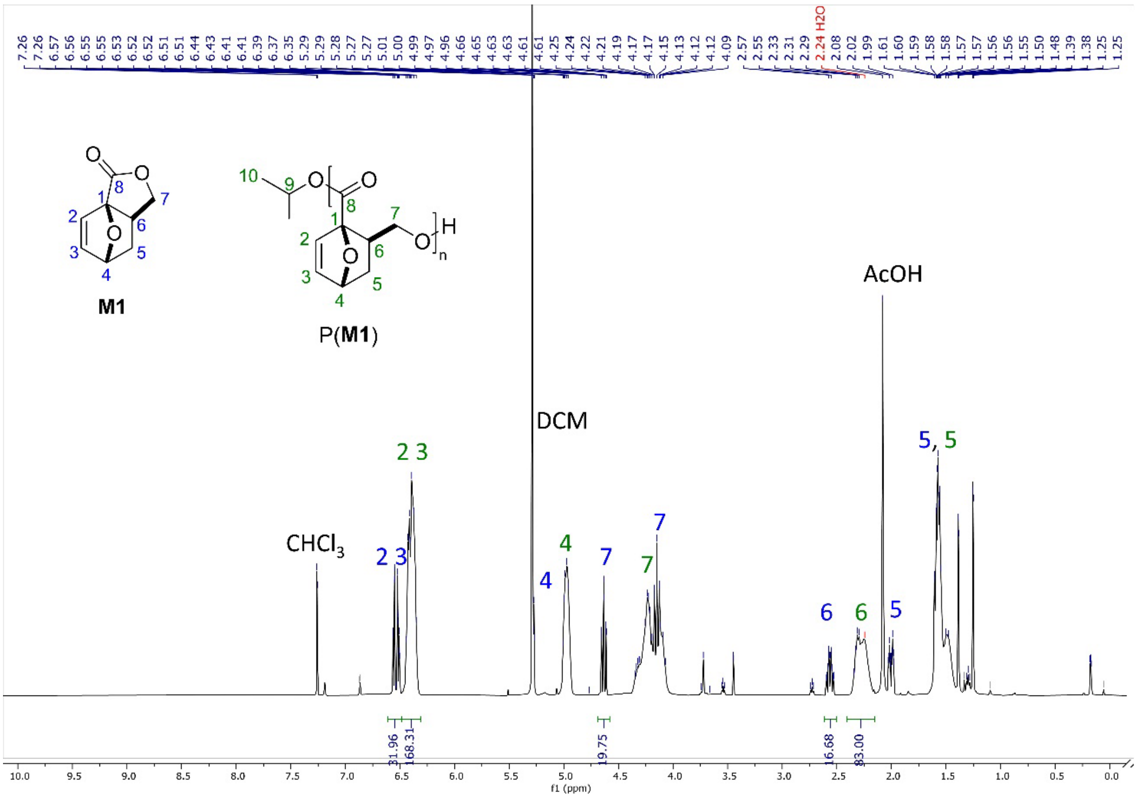

Figure S19: Crude ^{1}H NMR ($CDCl_3$) spectrum of P(**M1**) obtained with **M1**/Y-**1**/^{i}PrOH = 100/1/1, (Table 1 entry 9); 83% conversion to polymer.

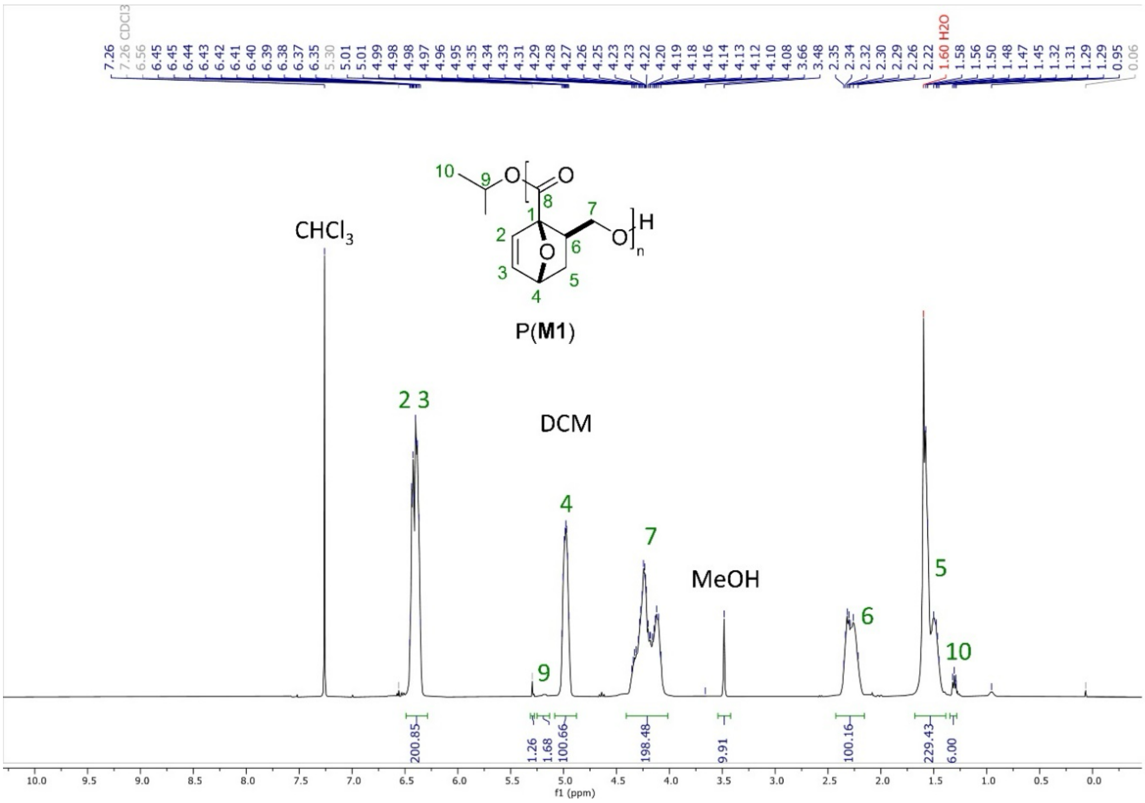

Figure S20: ^{1}H NMR ($CDCl_3$) spectrum of isolated P(**M1**) obtained with **M1**/Y-**1**/^{i}PrOH = 100/1/1, (Table 1, entry 9); $M_{n,NMR}$ = 15376 g mol^{-1}. Isopropyl end-group (9, 10) assignment confirmed by COSY.

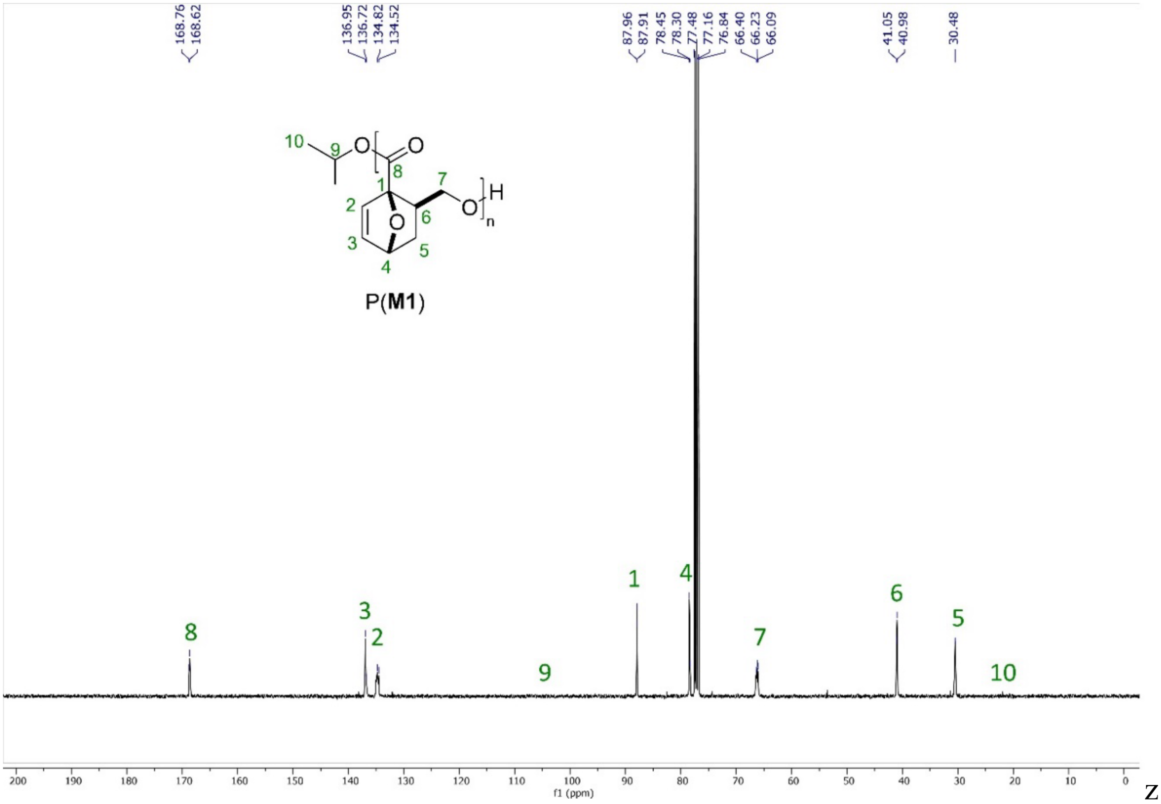

Figure S21: $^{13}C\{^1H\}$ NMR ($CDCl_3$) spectrum of isolated P(**M1**) obtained with **M1**/Y-**1**/^{i}PrOH = 100/1/1 (Table 1, entry 9).

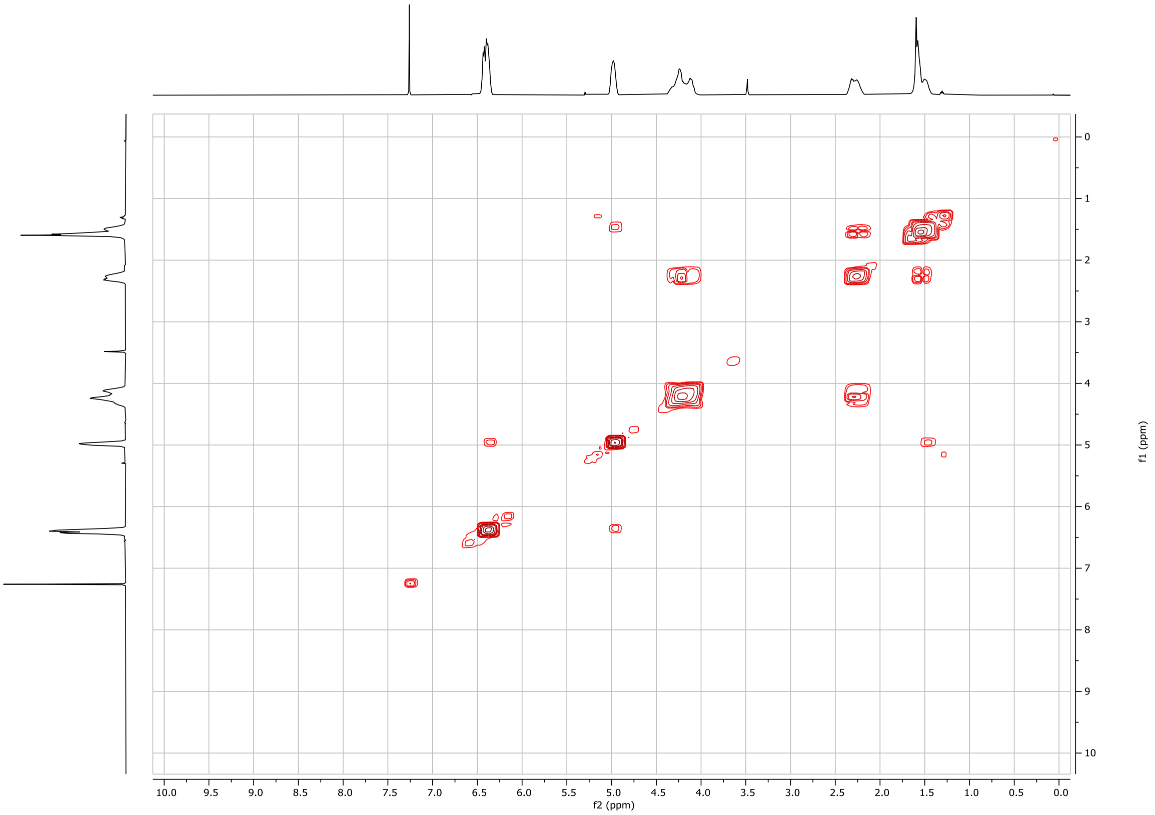

Figure S22: ^1H-^1H COSY ($CDCl_3$) spectrum of isolated P(**M1**) obtained with **M1**/Y-**1**/^{i}PrOH = 100/1/1 (Table 1, entry 9).

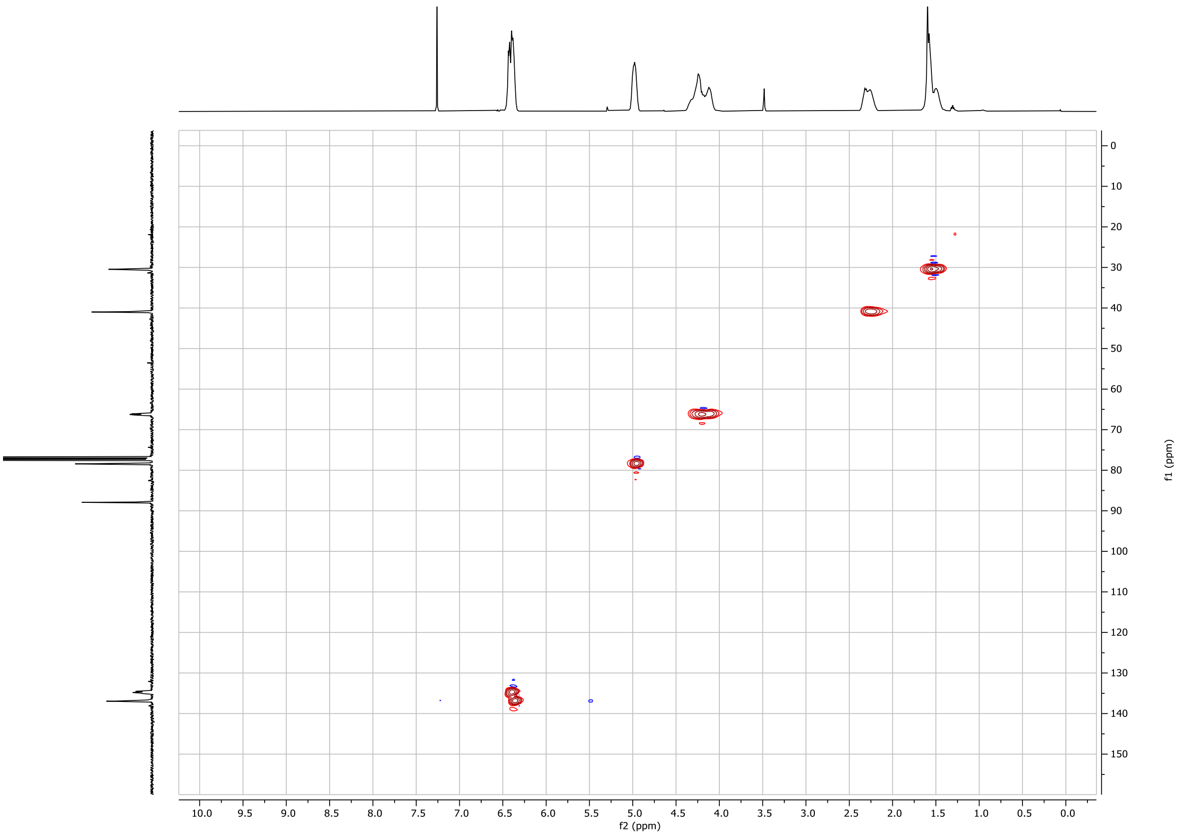

Figure S23: ASAP-HMQC ($CDCl_3$) spectrum of isolated P(**M1**) obtained with **M1**/Y-**1**/^{i}PrOH = 100/1/1 (Table 1, entry 9).

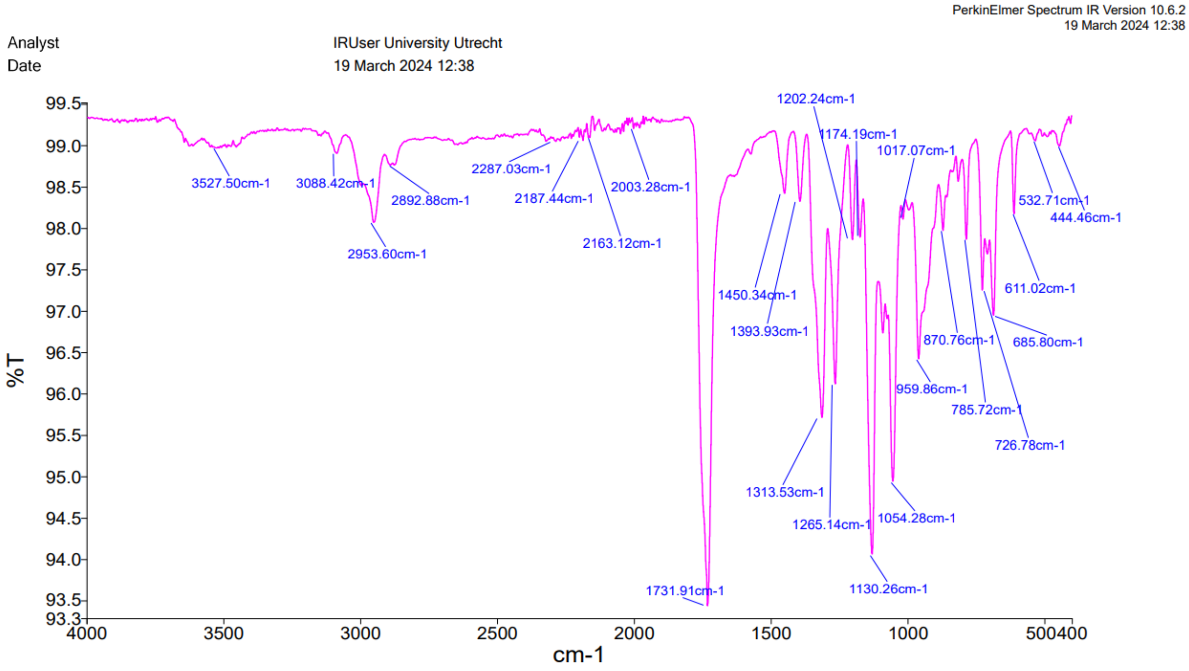

Figure S24: ATR-FTIR spectrum of isolated P(**M1**) obtained with **M1**/Y-**1**/^{i}PrOH = 100/1/1, (Table 1, entry 9).

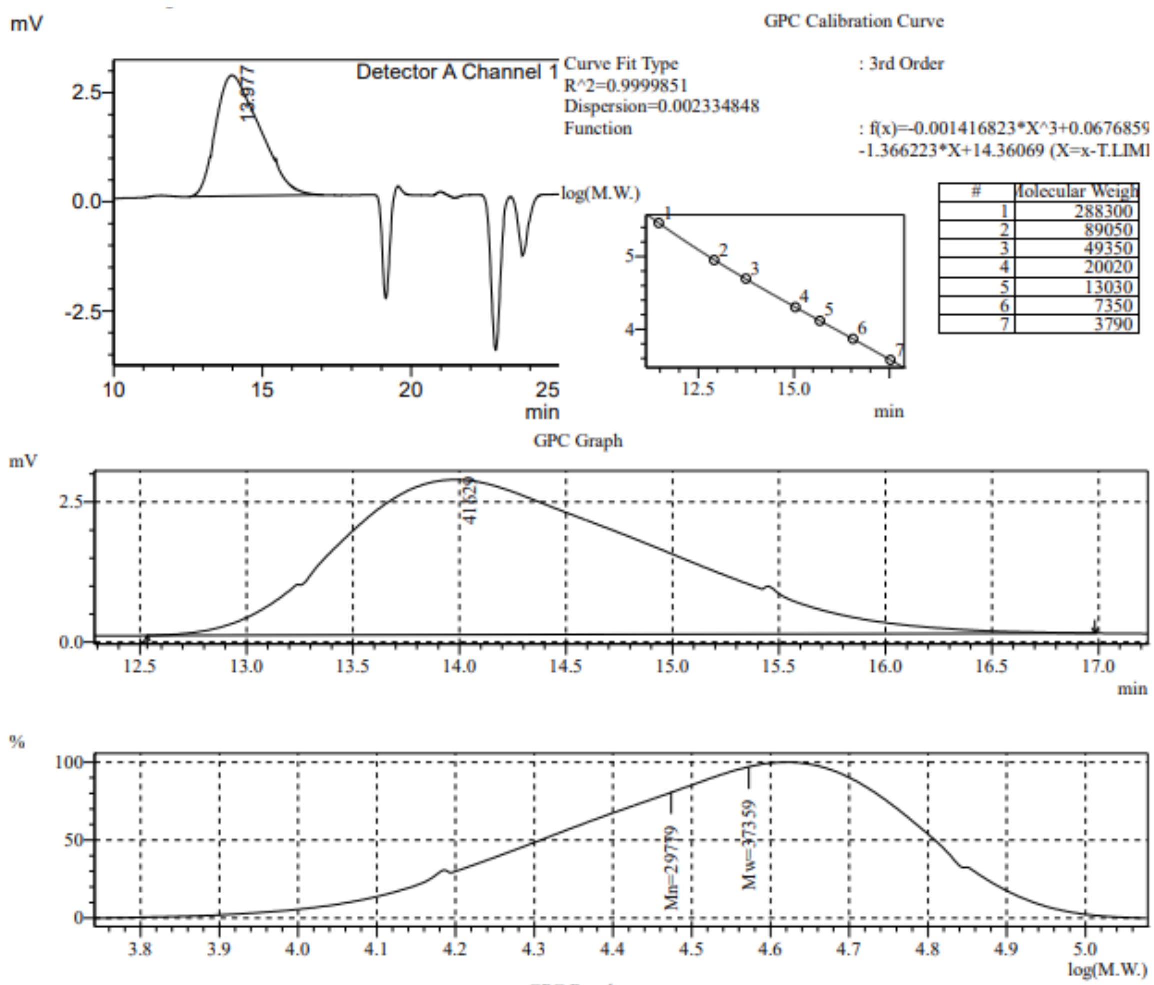

GPC Results

Peak#:1 (Detector A Channel 1)
[Peak Information]

Title	Time(min)	Molecular Weigl	Height
Start	12.533	120321	117
Top	13.977	41629	2769
End	16.983	5498	157

Area : 284171

[Average Molecular Weight]

Number Average Molecular Weight(Mn)	29779
Weight Average Molecular Weight(Mw)	37359
Mw/Mn	1.25453

Figure S25: GPC trace of P(**M1**) obtained with **M1**/Y-**1**/^{i}PrOH = 100/1/1, (Table 1 entry 9); and used calibration; $M_{n,GPC}$ = 29779 g mol^{-1}, Đ = 1.25

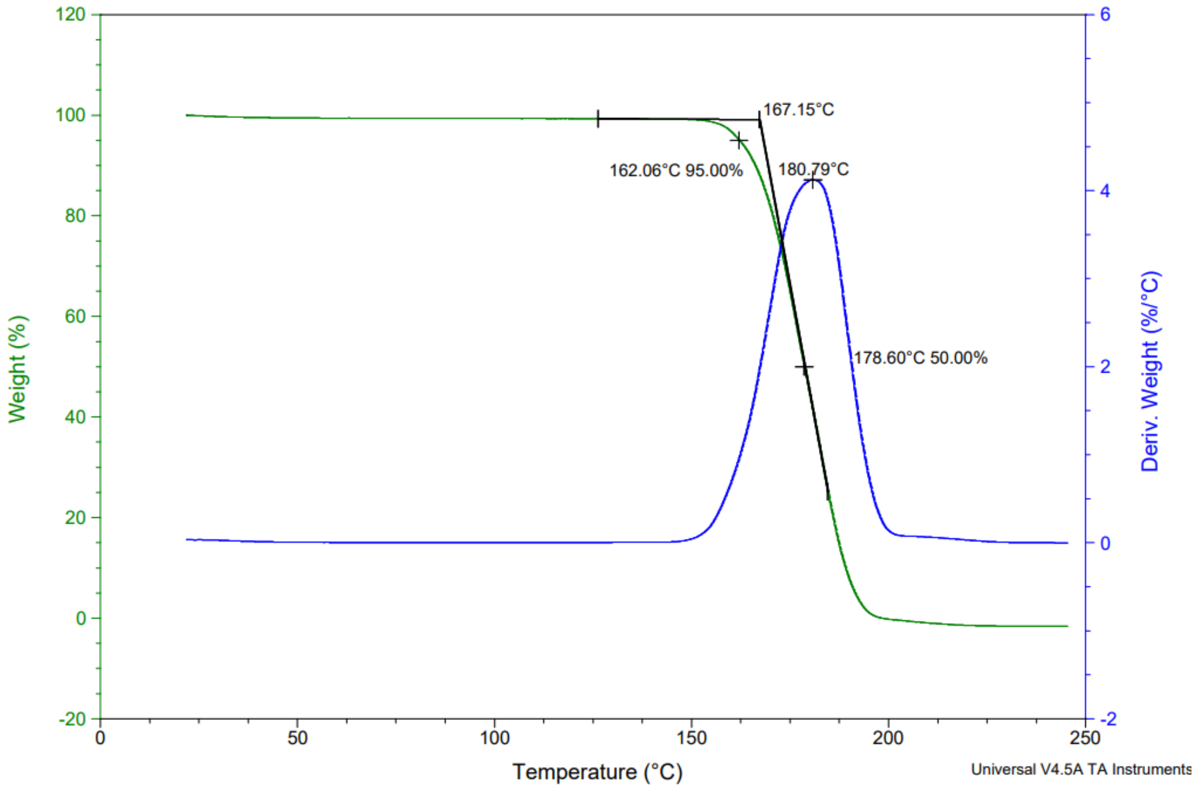

Figure S26: TGA (green) and DTG (blue) curves of P(**M1**) obtained with **M1**/Y-**1**/^{i}PrOH = 100/1/1, (Table 1, entry 9); T_{onset} = 167.2 °C, $T_{5\%}$ = 162.1 °C, $T_{50\%}$ = 178.6 °C, T_{max} = 180.8 °C.

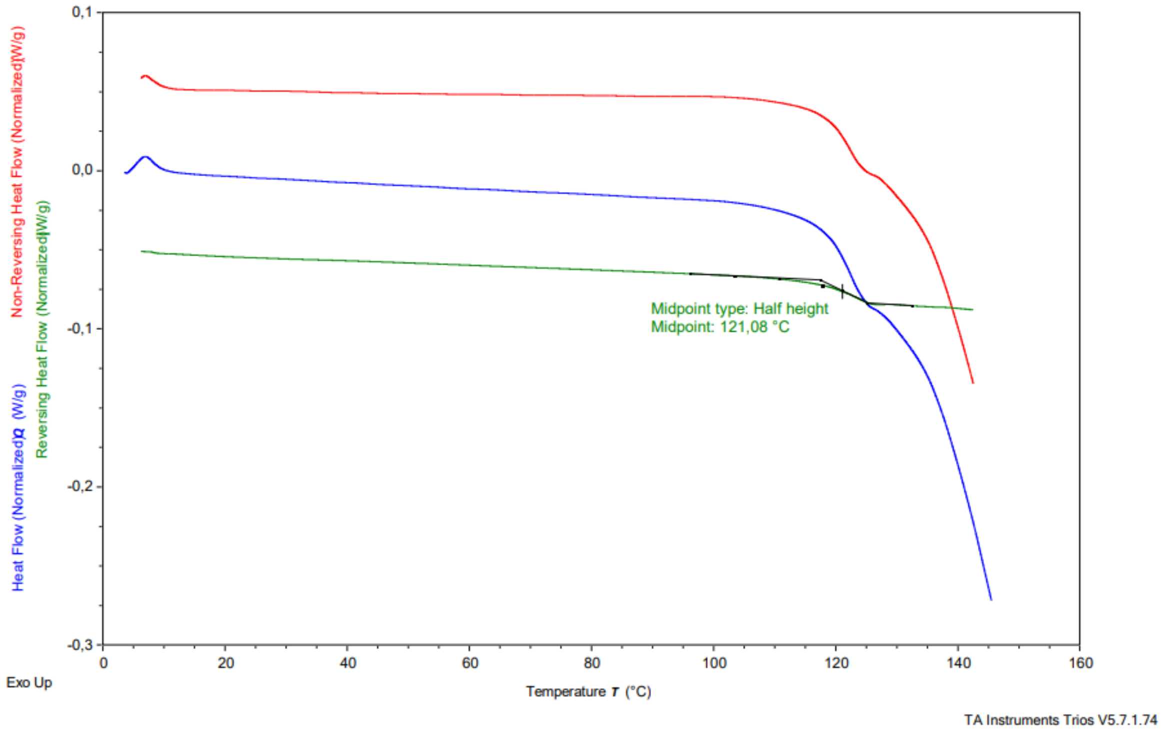

Figure S27: MDSC thermogram (second heating scan) of P(**M1**) obtained with **M1**/Y-**1**/^{i}PrOH = 100/1/1, (Table 1, entry 9); T_g = 121.1 °C.

Hydrogenated polymer P(M1-H2) from post-polymerizaton hydrogenation

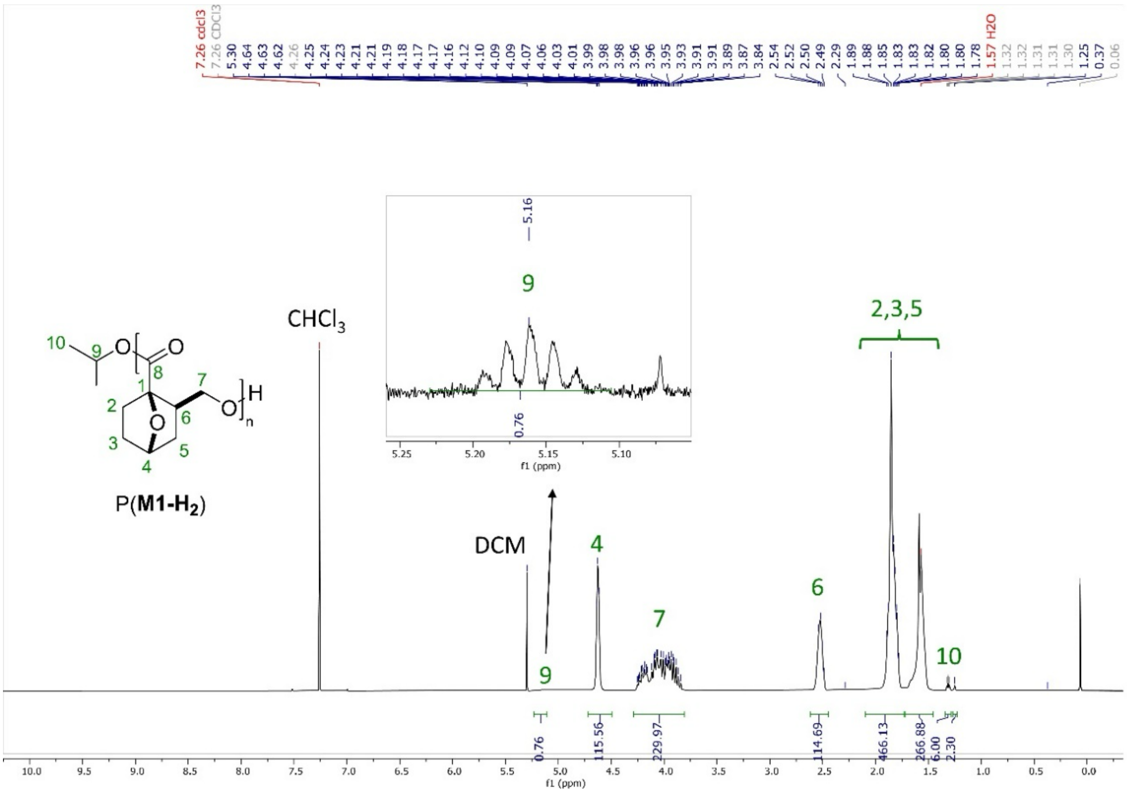

Figure S28: ^{1}H NMR ($CDCl_3$) spectrum of P(**M1-H₂**) obtained by hydrogenation of P(**M1**) (formed by **M1**/Y-**1**/^{i}PrOH = 100/1/1, Table 1, entry 9); $M_{n,NMR}$ = 15376 g mol^{-1}.

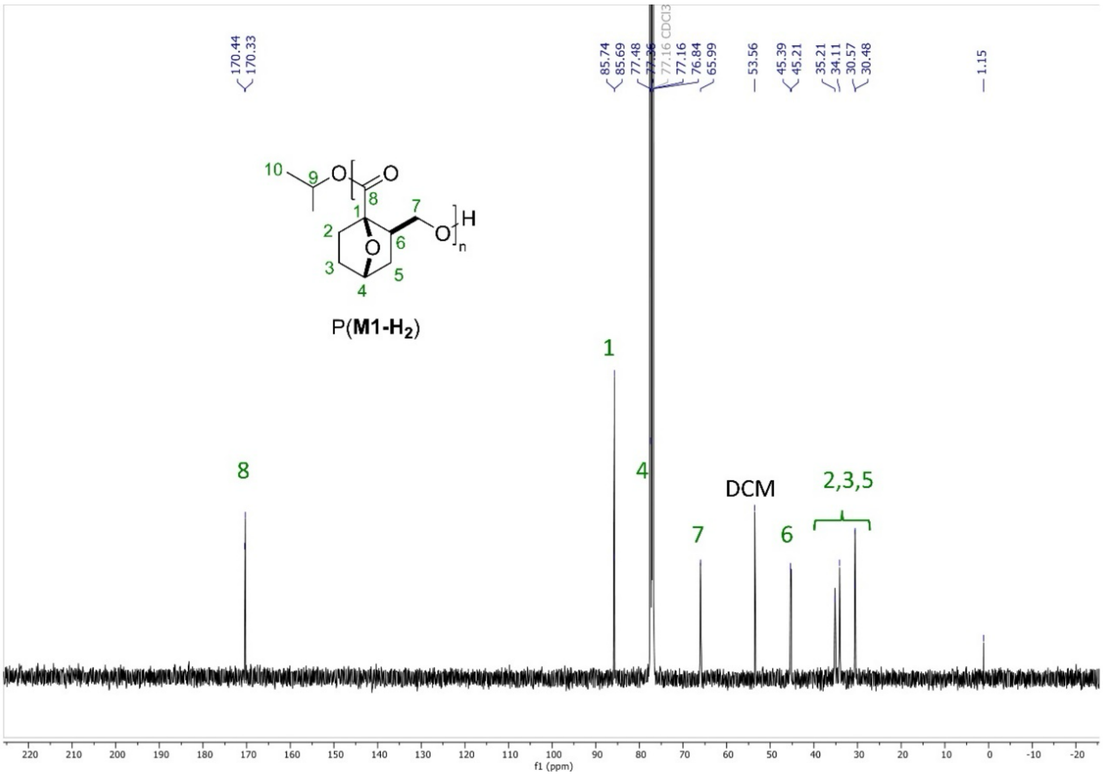

Figure S29: $^{13}C\{^1H\}$ NMR ($CDCl_3$) spectrum of P(**M1-H₂**) obtained by hydrogenation of P(**M1**) (**M1**/Y-**1**/^{i}PrOH = 100/1/1, Table 1, entry 9).

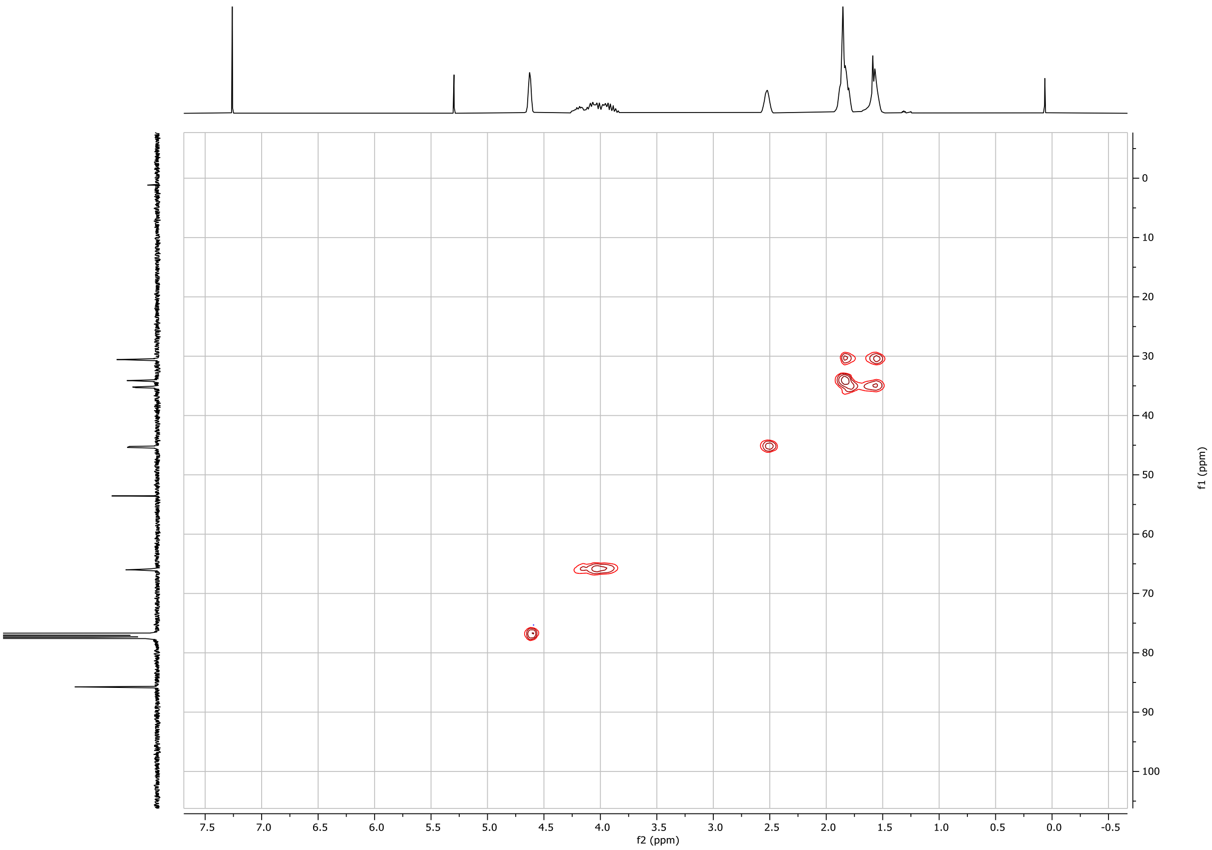

Figure S30: ASAP-HMQC ($CDCl_3$) spectrum of P(**M1-H₂**) obtained by hydrogenation of P(**M1**) (**M1**/Y-**1**/^{i}PrOH = 100/1/1, Table 1, entry 9).

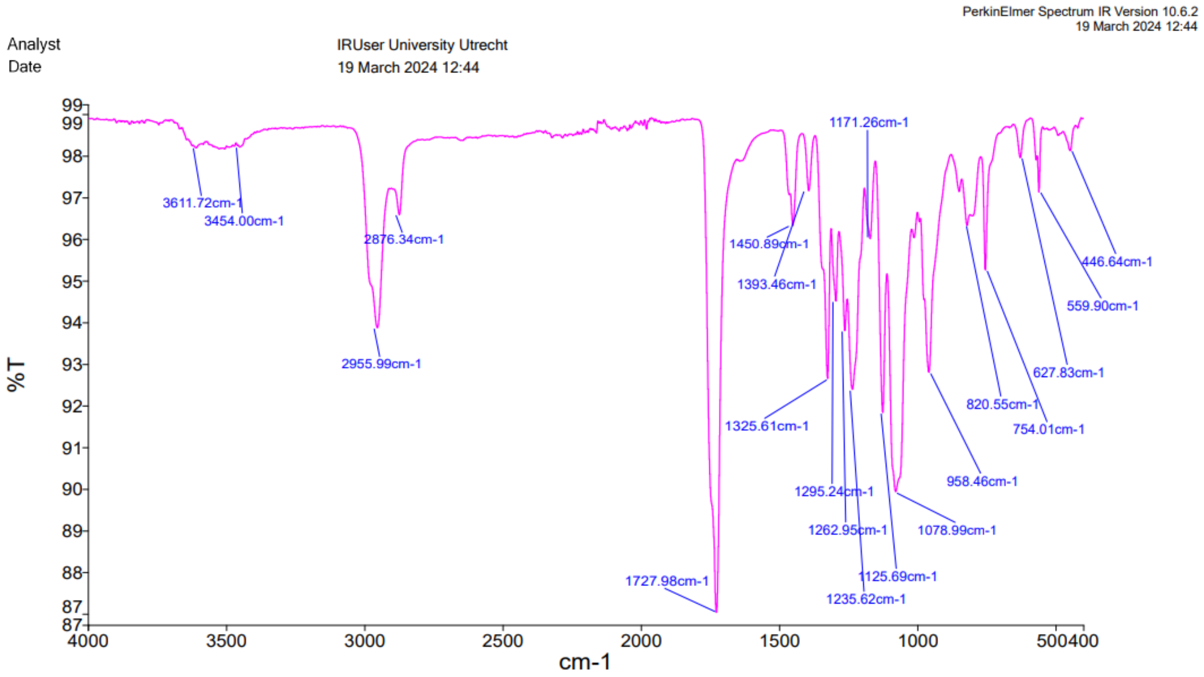

Figure S31: ATR-FTIR spectrum of P(**M1-H₂**) obtained by hydrogenation of P(**M1**) (**M1**/Y-**1**/^{i}PrOH = 100/1/1, Table 1, entry 9).

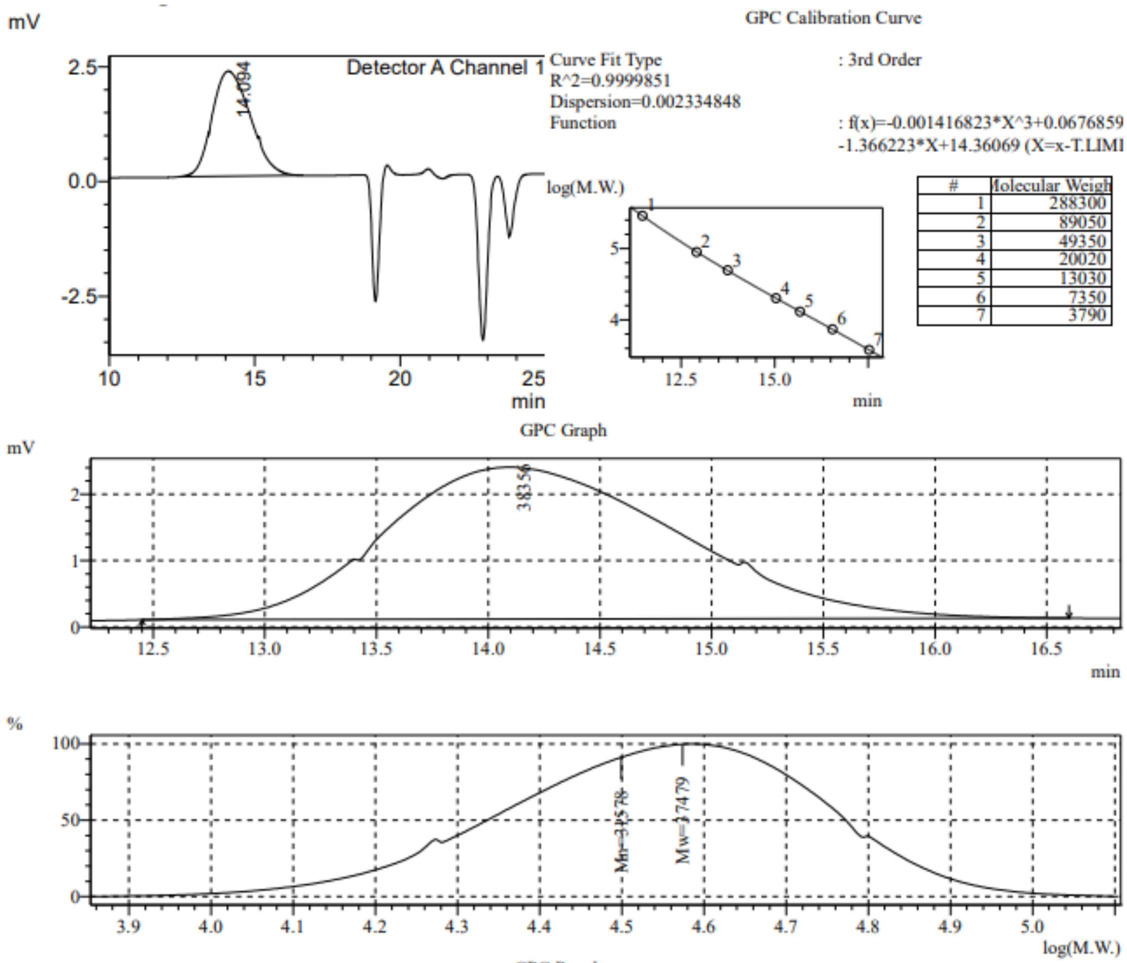

GPC Results

Peak#:1 (Detector A Channel 1)

[Peak Information]

Title	Time(min)	Molecular Weigl	Height
Start	12.450	128394	108
Top	14.094	38356	2290
End	16.600	7111	133

Area : 209082

[Average Molecular Weight]

Number Average Molecular Weight(Mn)	31578
Weight Average Molecular Weight(Mw)	37479
Mw/Mn	1.18687

Figure S32: GPC trace of P(**M1-H$_2$**) obtained by hydrogenation of P(**M1**) (**M1**/Y-**1**/^{i}PrOH = 100/1/1, Table 1, entry 9); and used calibration; $M_{n,GPC}$ = 31578 g mol^{-1}, Đ = 1.19.

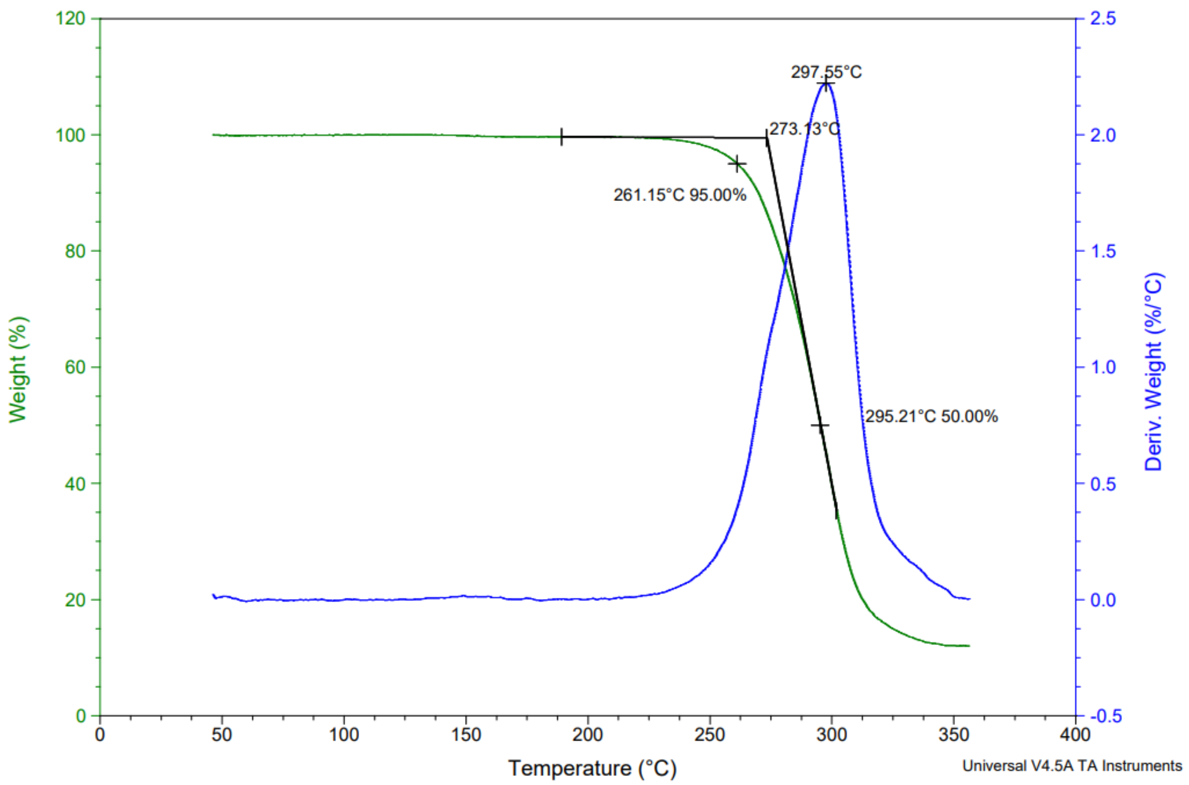

Figure S33: TGA (green) and DTG (blue) curves of P(**M1-H2**) obtained by hydrogenation of P(**M1**) (**M1**/Y-**1**/^{i}PrOH = 100/1/1, Table 1, entry 9); T_{onset} = 273.1 °C, $T_{5\%}$ = 261.2 °C, $T_{50\%}$ = 295.2 °C, T_{max} = 297.6 °C.

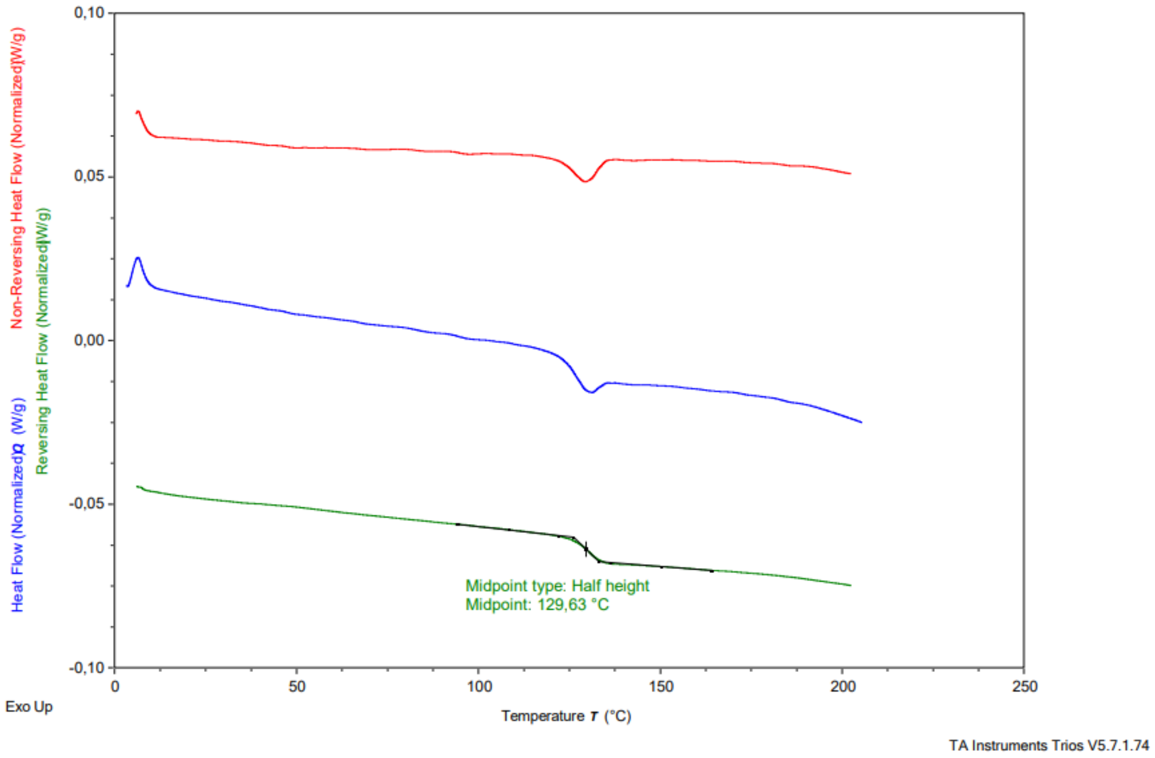

Figure S34: MDSC thermogram (second heating scan) of P(**M1-H2**) obtained by hydrogenation of P(**M1**) (**M1**/Y-**1**/^{i}PrOH = 100/1/1, Table 1, entry 9); T_g = 129.6 °C.

Hydrogenated polymer P(M1-H$_2$) from polymerization of M1-H$_2$

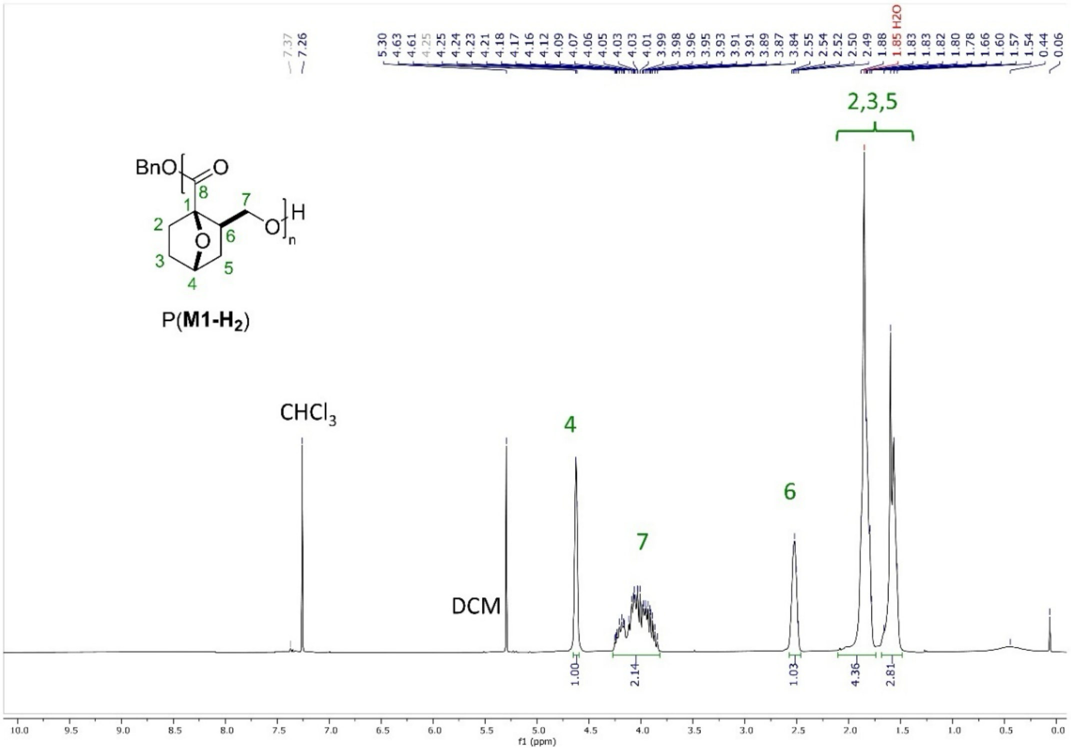

Figure S35: ^{1}H NMR (CDCl$_3$) spectrum of P(**M1-H$_2$**) obtained by polymerization of **M1-H$_2$** at -40 °C (see Table S9); $M_{n,NMR}$ = 47314 g mol^{-1}.

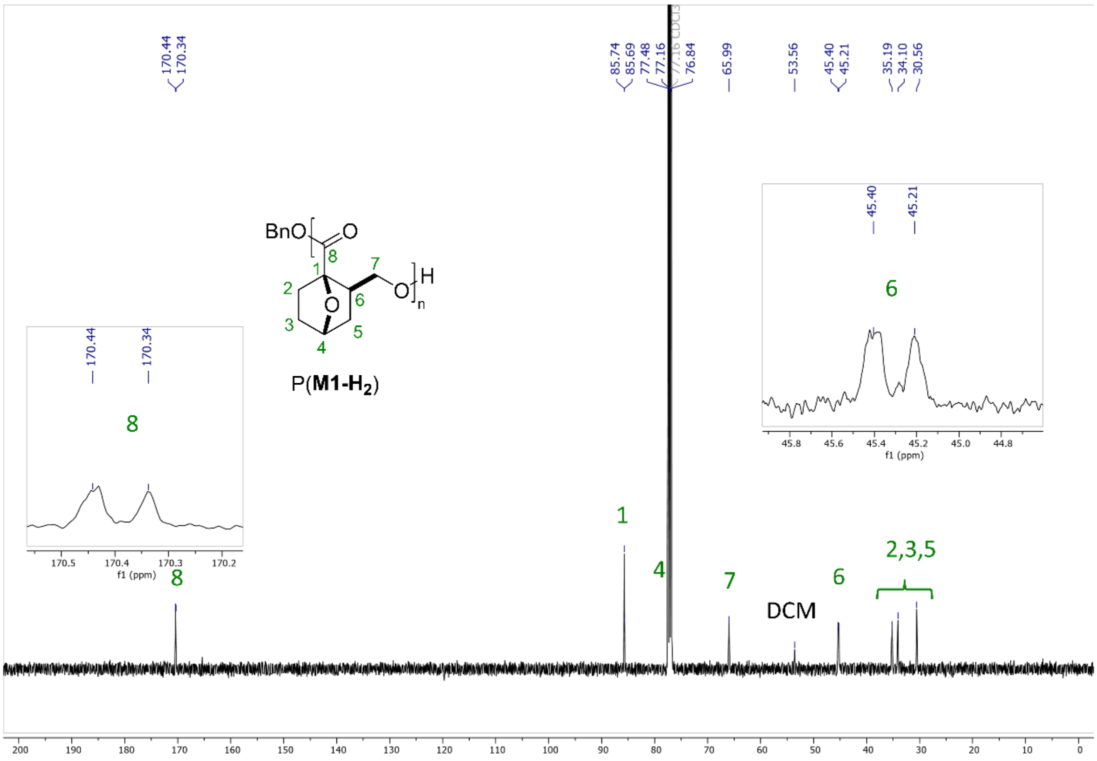

Figure S36: ^{13}C{^{1}H} NMR (CDCl$_3$) spectrum of P(**M1-H$_2$**) obtained by polymerization of **M1-H$_2$** at -40 °C (see Table S9).

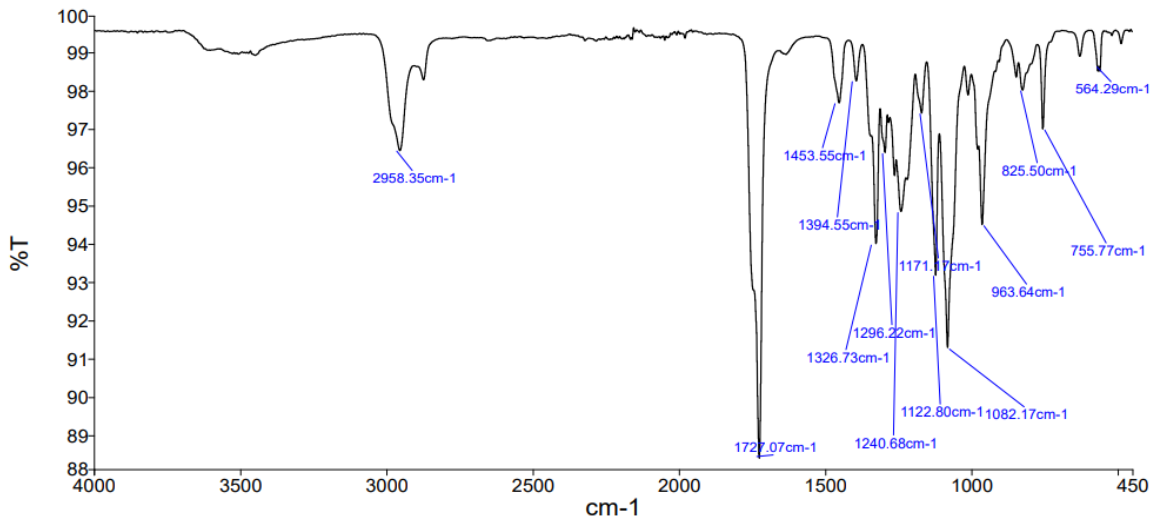

Figure S37: ATR-FTIR spectrum of P(**M1-H$_2$**) obtained by polymerization of **M1-H$_2$** at -40 °C (see Table S9).

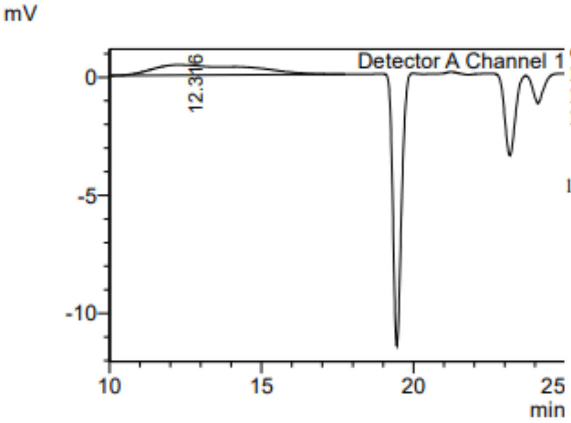

GPC Calibration Curve

Curve Fit Type : 3rd Order
R^2=0.9999834
Dispersion=0.002461034
Function : f(x)=-0.001291682*X^3+0.0628558
-1.307738*X+14.21107 (X=x-T.LIMI

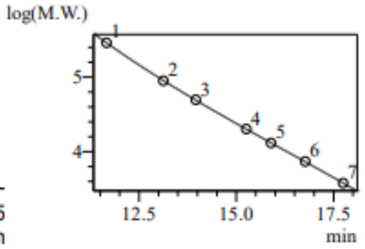

#	Iolecular Weigh
1	288300
2	89050
3	49350
4	20020
5	13030
6	7350
7	3790

GPC Graph

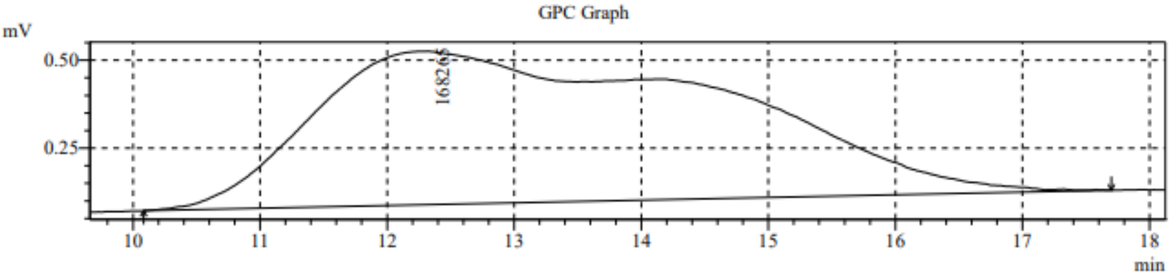

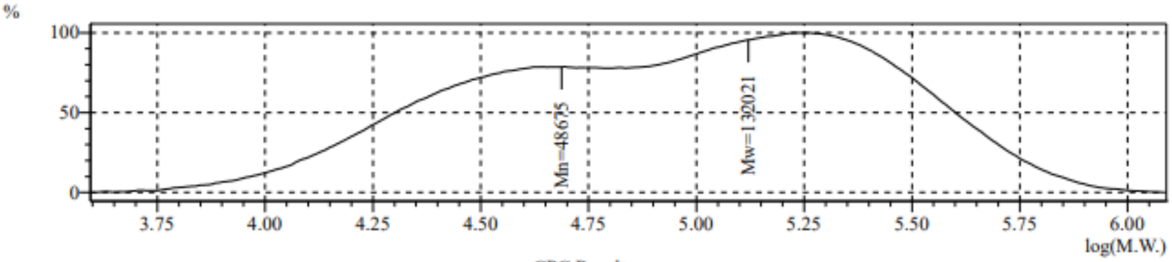

GPC Results

Peak#:1 (Detector A Channel 1)

[Peak Information]

Title	Time(min)	Iolecular Weigl	Height
Start	10.083	1233804	72
Top	12.316	168265	436
End	17.700	3922	131

Area : 97241

[Average Molecular Weight]

Number Average Molecular Weight(Mn)	48675
Weight Average Molecular Weight(Mw)	132021
Mw/Mn	2.71232

Figure S38: GPC trace of P(**M1-H₂**) obtained by polymerization of **M1-H₂** at -40 °C (see Table S9); and used calibration; $M_{n,GPC}$ = 48675 g mol^{-1}, Đ = 2.71

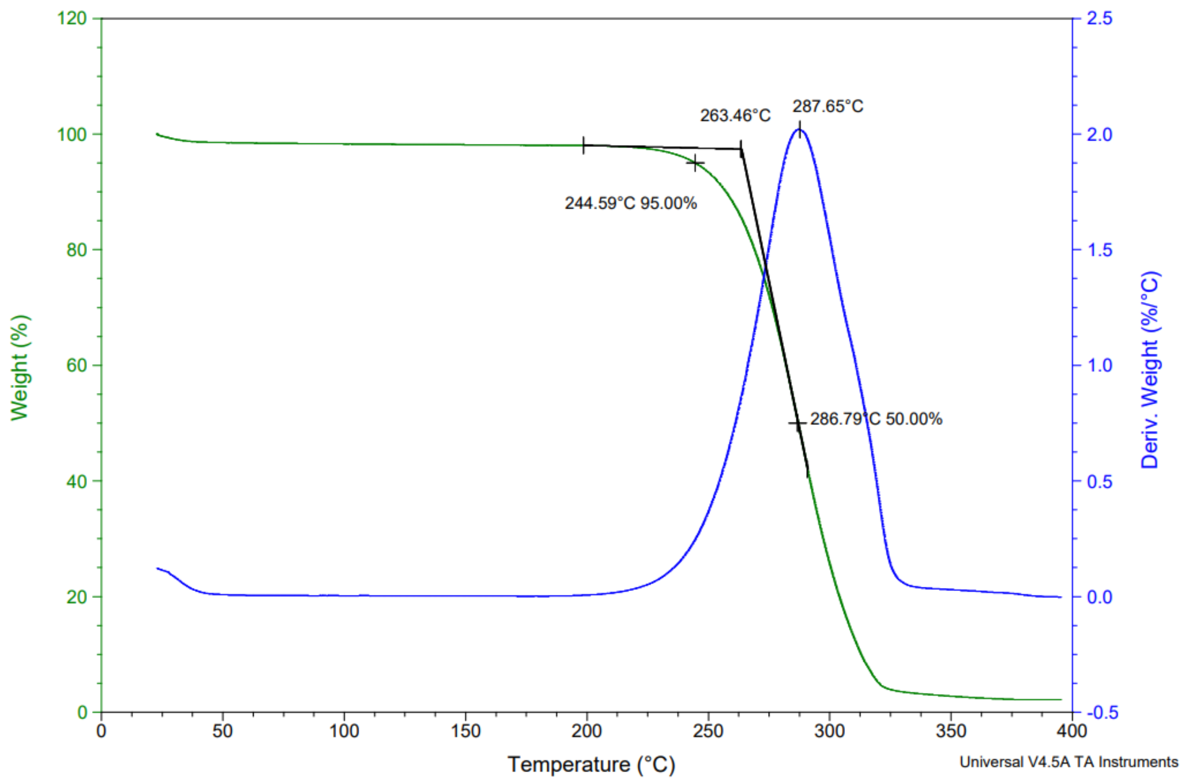

Figure S39: TGA (green) and DTG (blue) curves of P(**M1-H**$_2$) obtained by polymerization of **M1-H**$_2$ at -40 °C (see Table S9); T_{onset} = 263.5 °C, $T_{5\%}$ = 244.6 °C, $T_{50\%}$ = 286.8 °C, T_{max} = 287.7 °C.

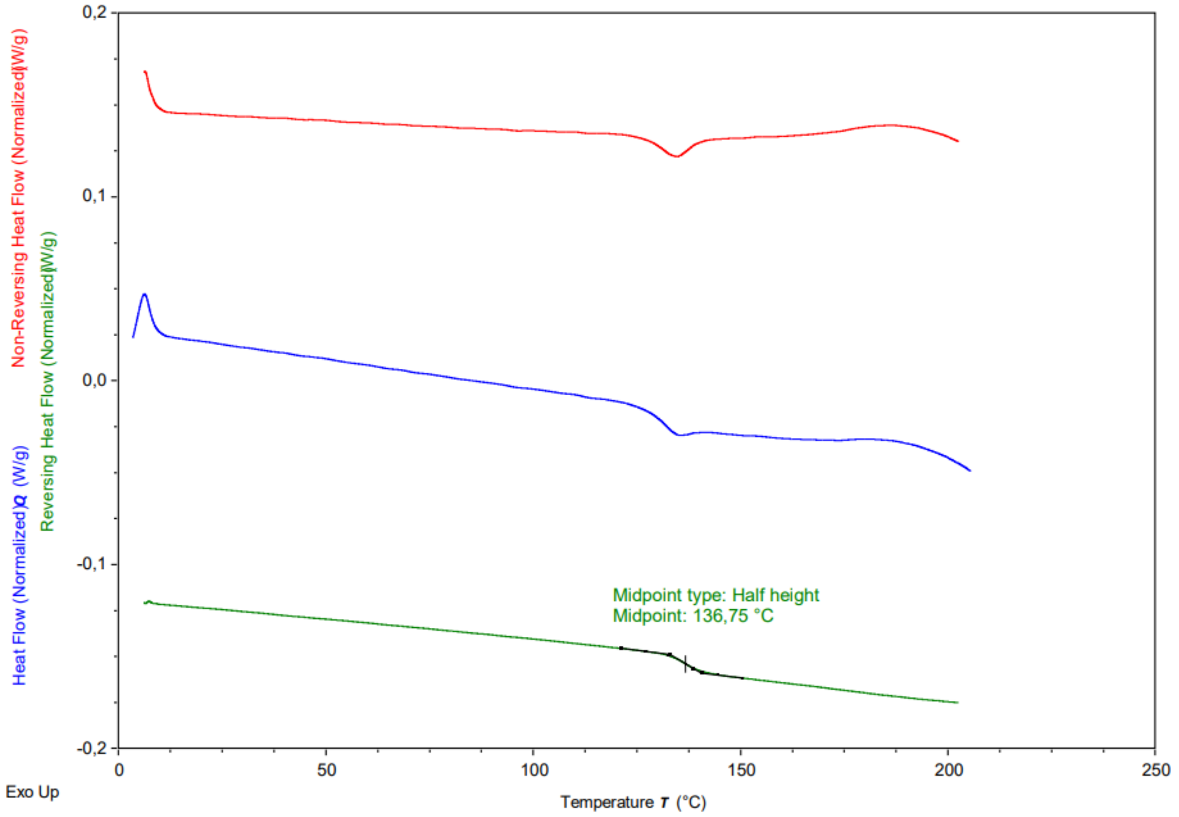

Figure S40: MDSC thermogram (second heating scan) of P(**M1-H**$_2$) obtained by polymerization of **M1-H**$_2$ at -40 °C (See Table S9); T_g = 136.8 °C.

Characterization cyclic dimer (M1)$_2$

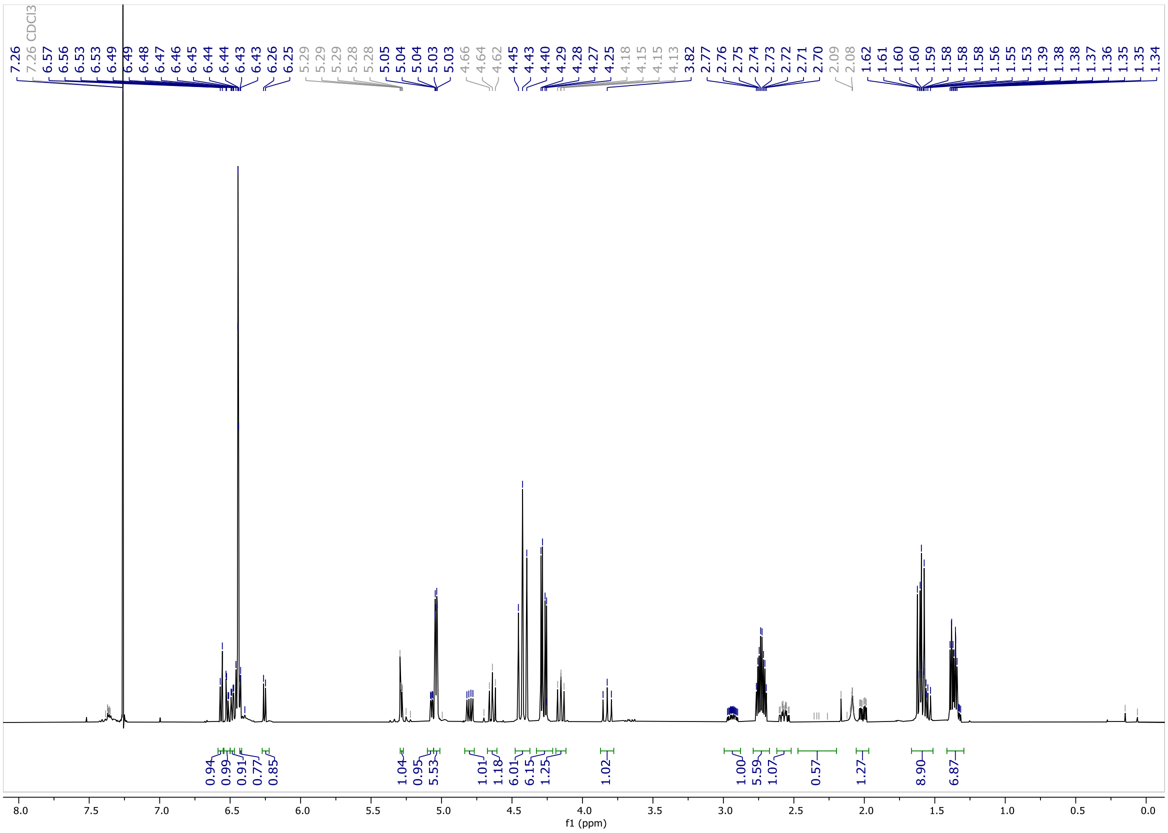

Figure S41: Crude ^{1}H NMR ($CDCl_3$) spectrum of the formation of cyclic dimer (**M1**)$_2$

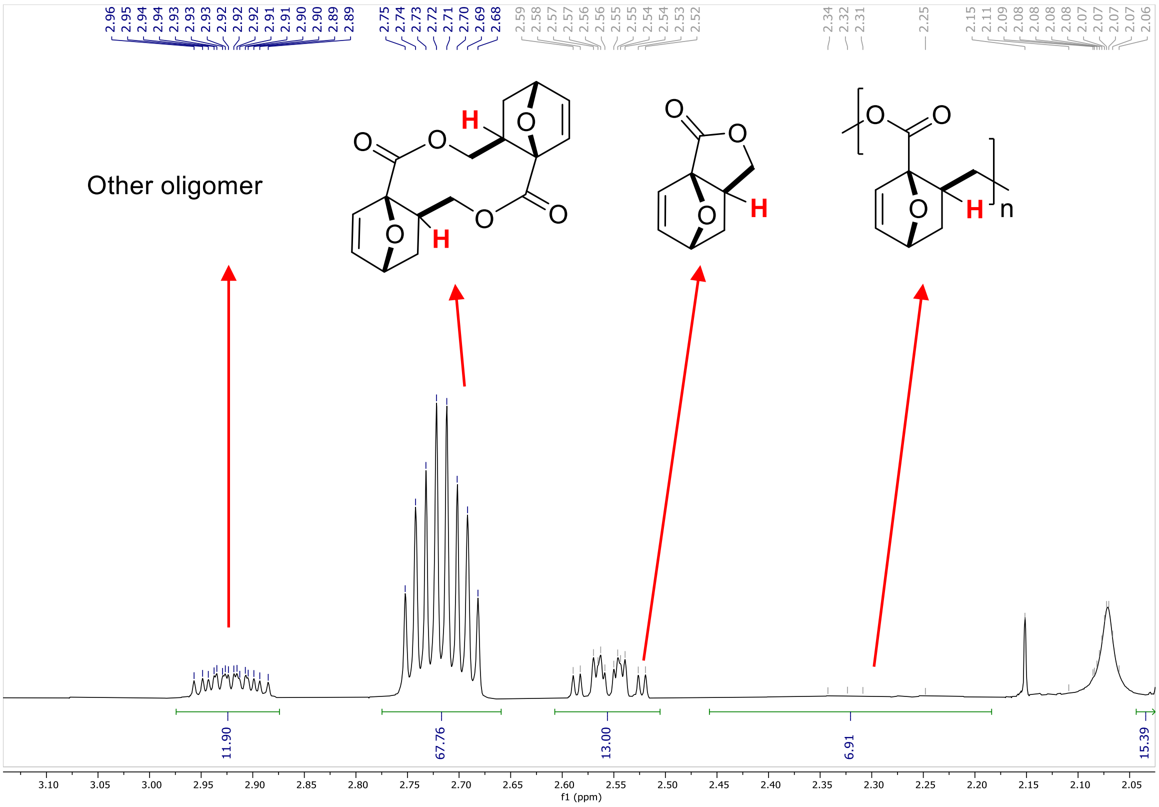

Figure S42: Zoom and assignment of crude ^{1}H NMR ($CDCl_3$) spectrum of the formation of cyclic dimer (**M1**)$_2$.

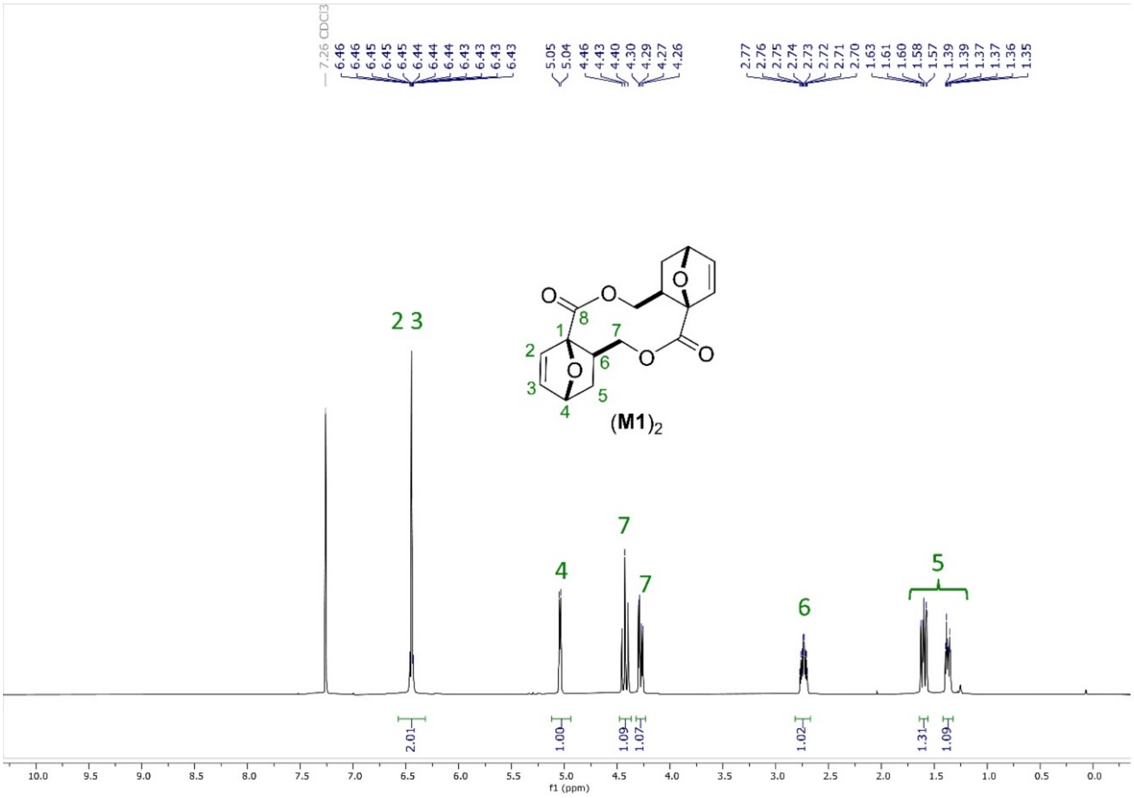

Figure S43: ^{1}H NMR ($CDCl_3$) spectrum of cyclic dimer (**M1**)$_2$.

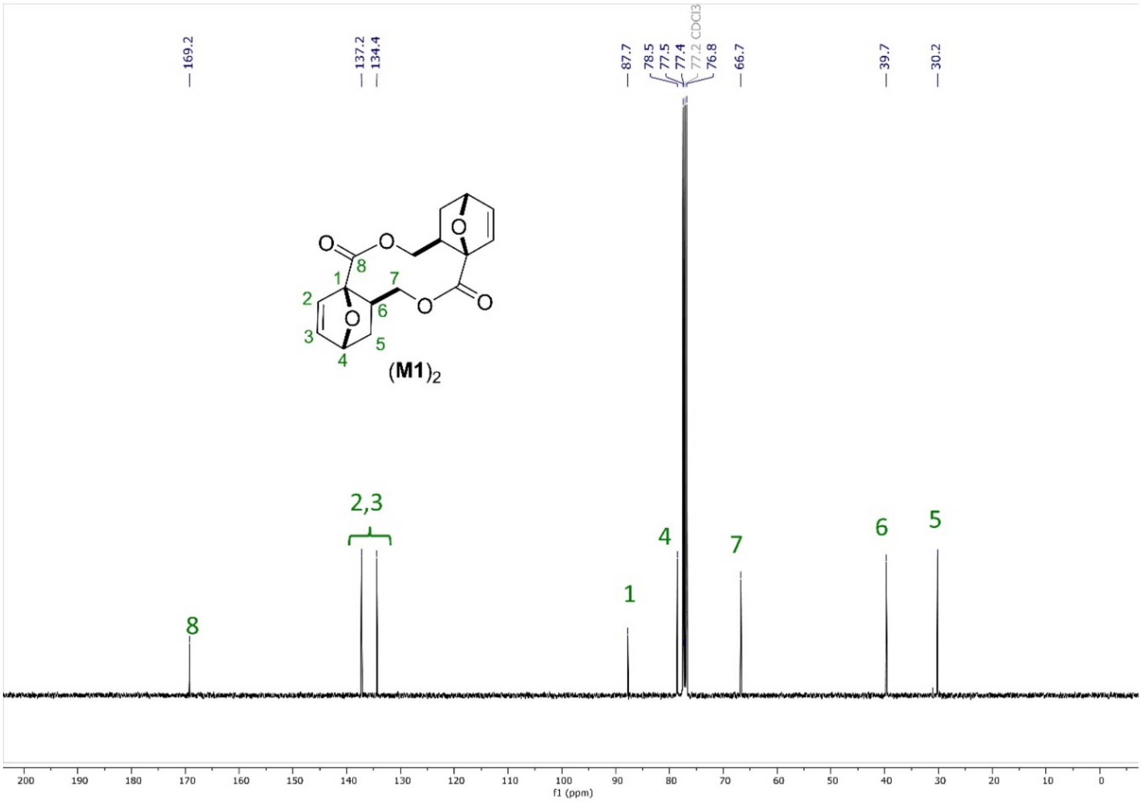

Figure S44: $^{13}C\{^{1}H\}$ NMR ($CDCl_3$) spectrum of cyclic dimer (**M1**)$_2$.

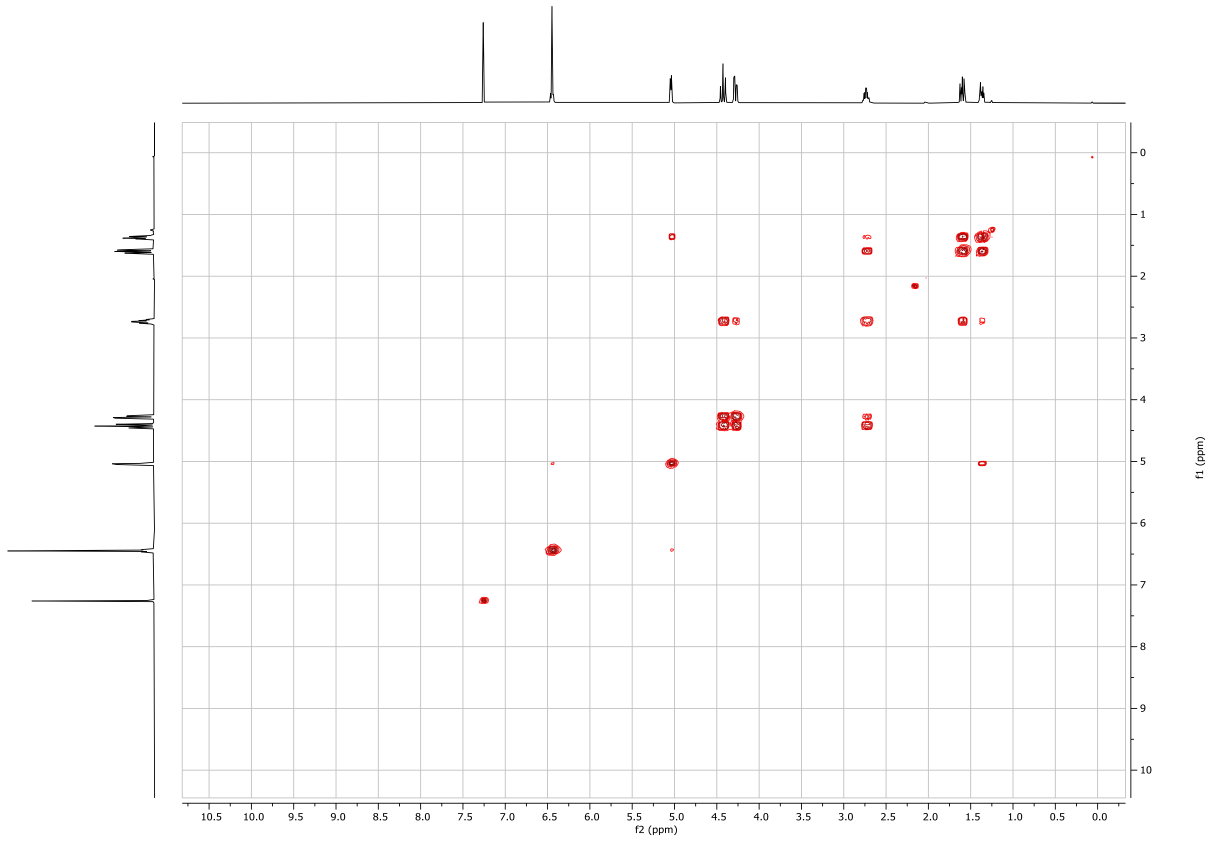

Figure S45: ^{1}H-^{1}H-COSY NMR ($CDCl_3$) spectrum of cyclic dimer (**M1**)$_2$.

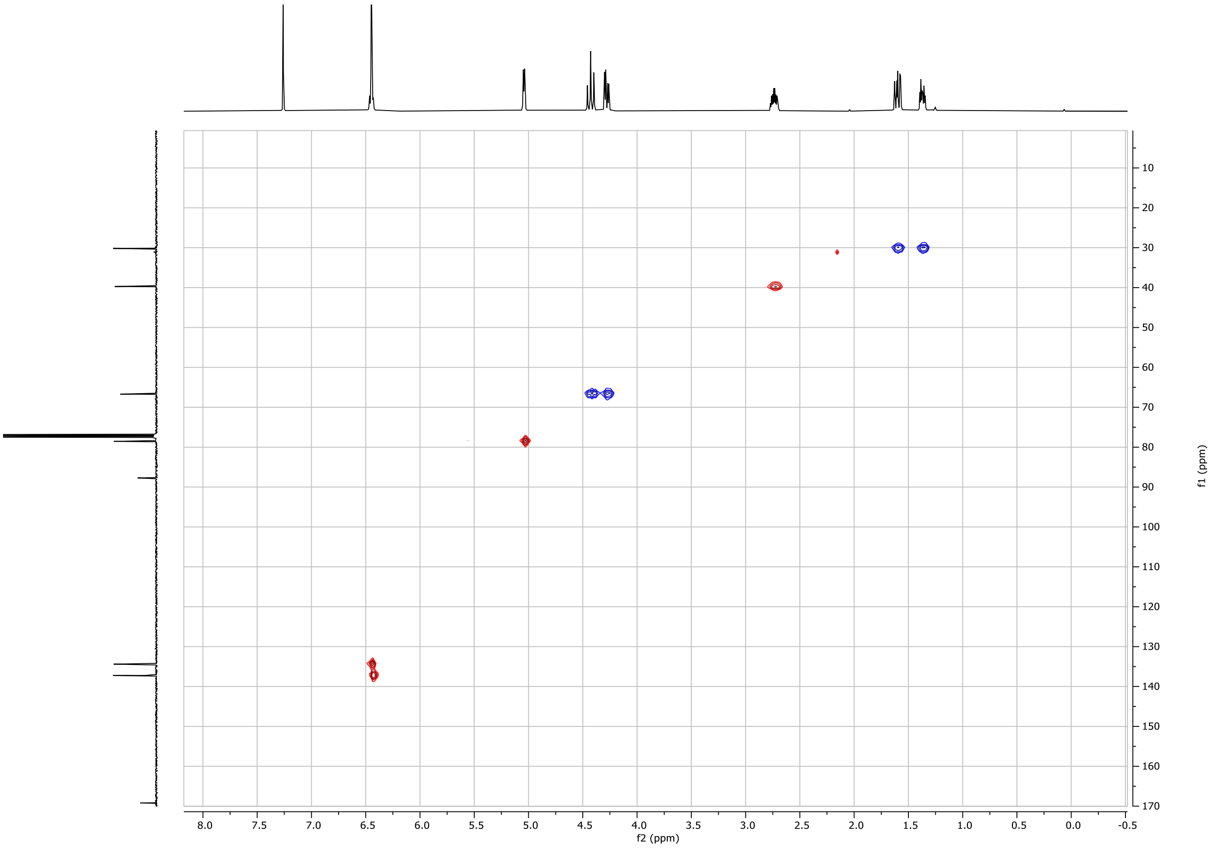

Figure S46: HSQC NMR ($CDCl_3$) spectrum of cyclic dimer (**M1**)$_2$.

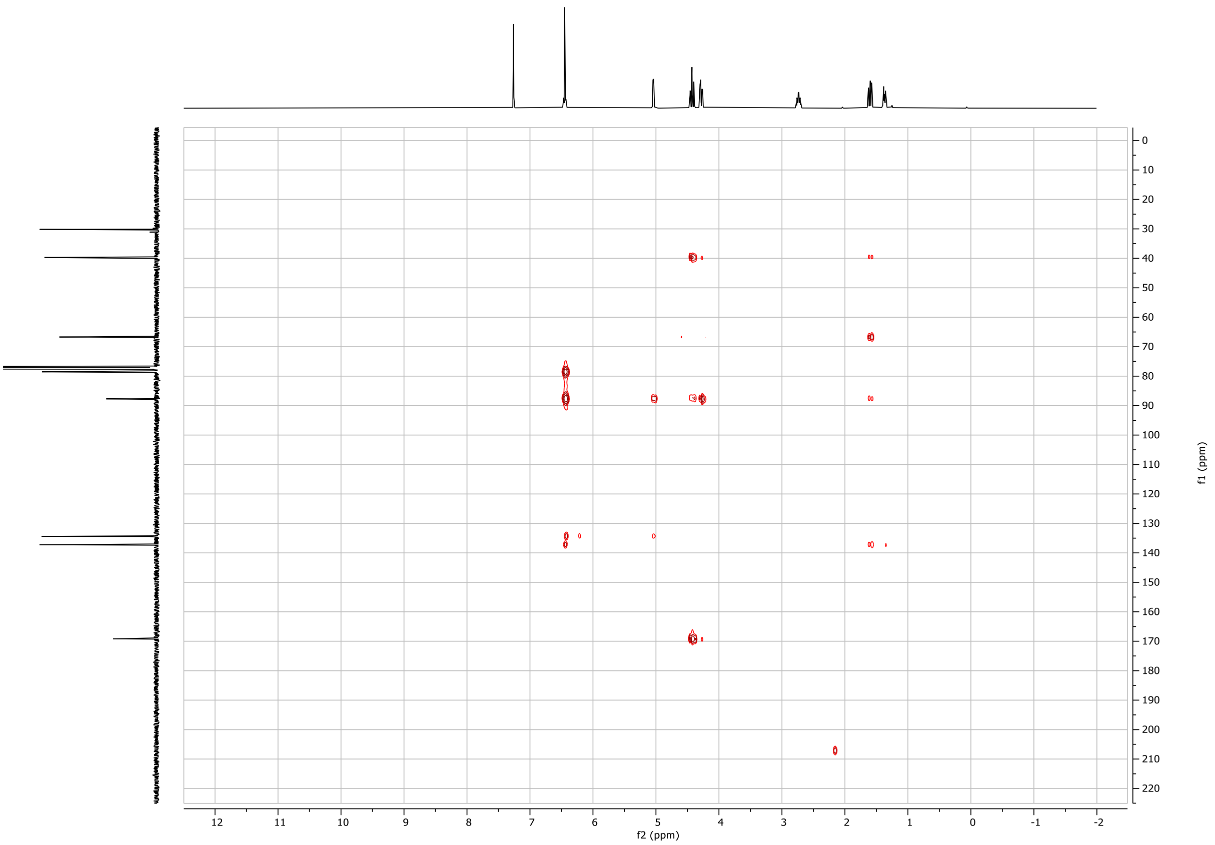

Figure S47: HMBC NMR ($CDCl_3$) spectrum of cyclic dimer (**M1**)$_2$.

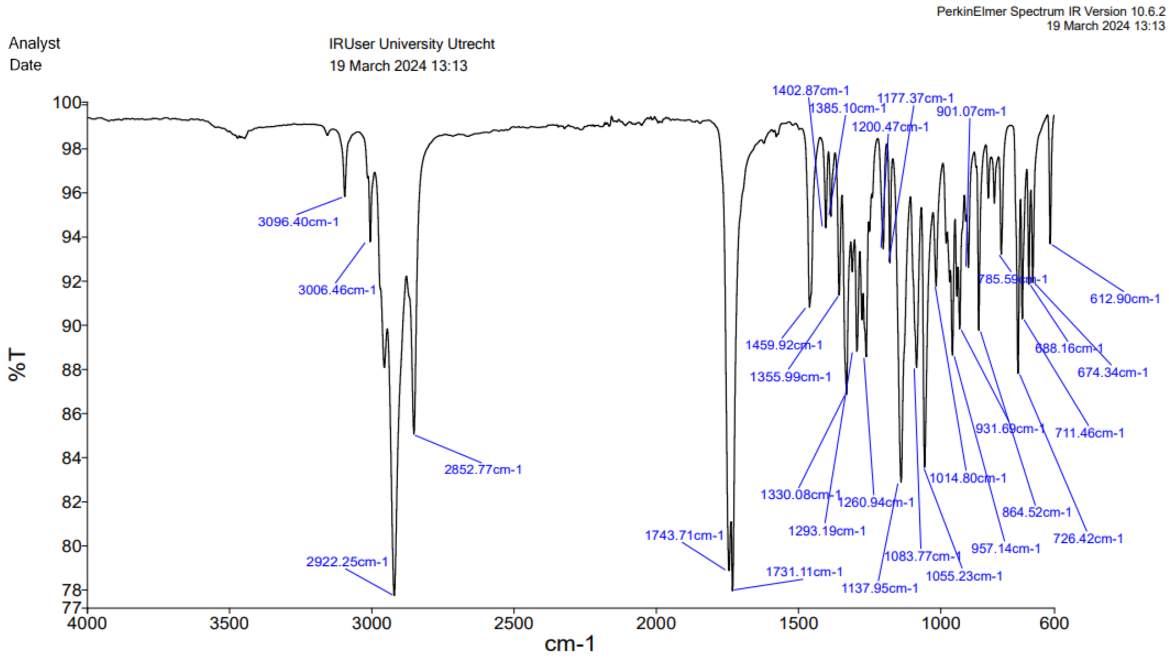

Figure S48: ATR-FTIR spectrum of cyclic dimer (**M1**)$_2$.

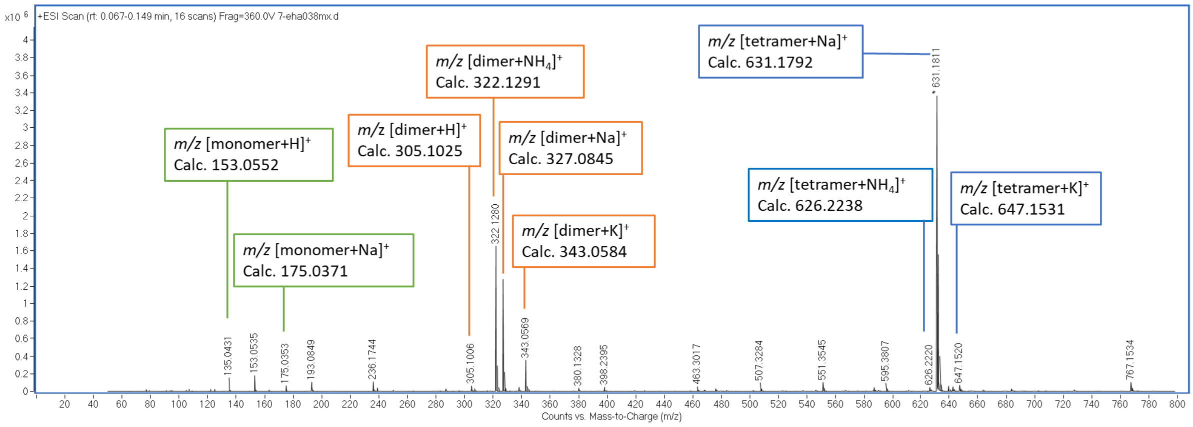

Figure S49: ESI-MS spectrum and assignment of cyclic dimer (**M1**)$_2$.

NMR spectra and GPC traces of thermal stability studies of P(M1)

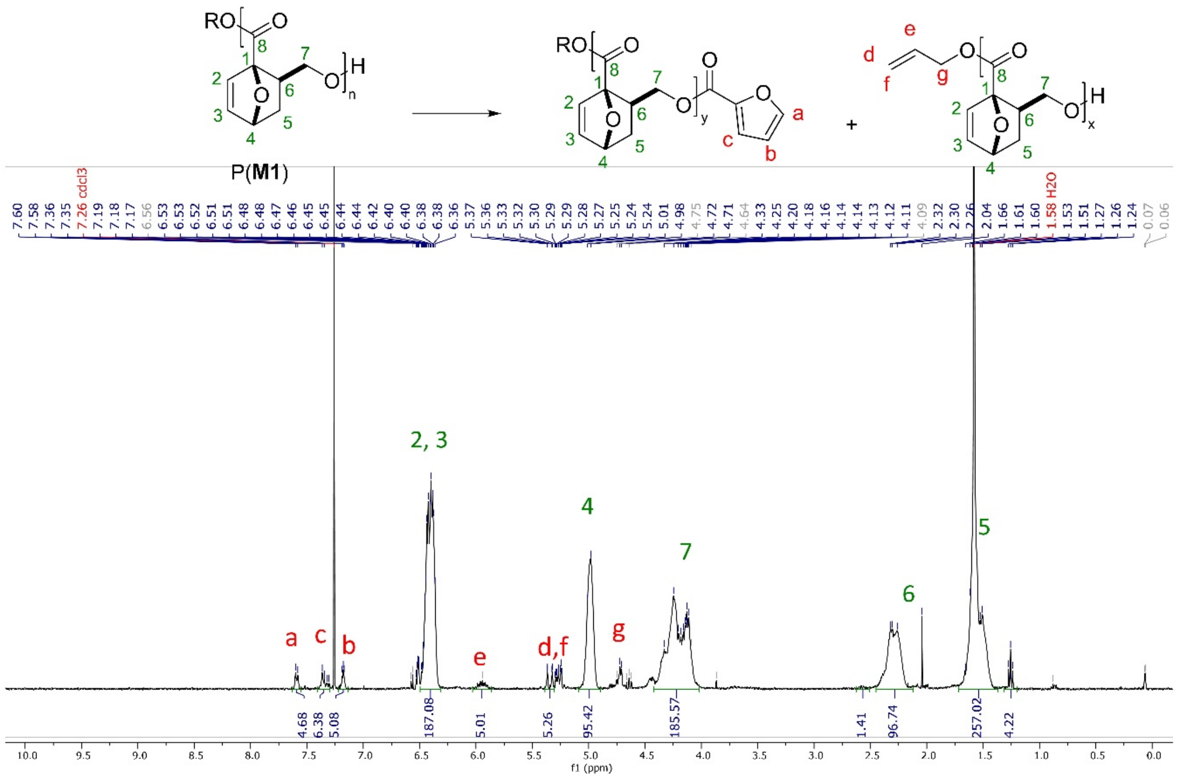

Figure S50: Thermal stability in solid state: ^{1}H NMR analysis indicated chain scission through the retro Diels-Alder reaction in P(**M1**) sample after heating to 130 °C in MDSC. The ratio of 4:a of 95:5 indicates 5% rDA.

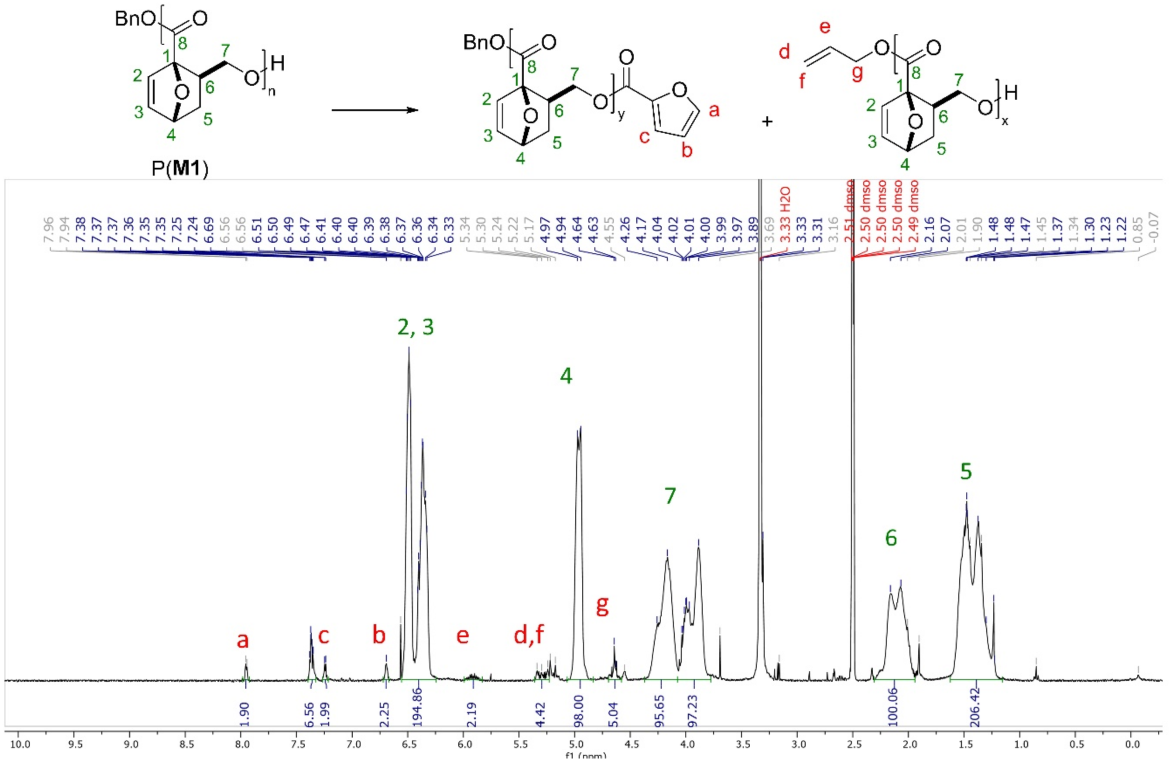

Figure S51: Thermal stability in solution state: ^{1}H NMR analysis indicated chain scission through the retro Diels-Alder reaction in P(**M1**) sample after heating for 1 h in DMSO-D_6. The ratio of 4:a of 98:2 indicates 2% rDA had taken place.

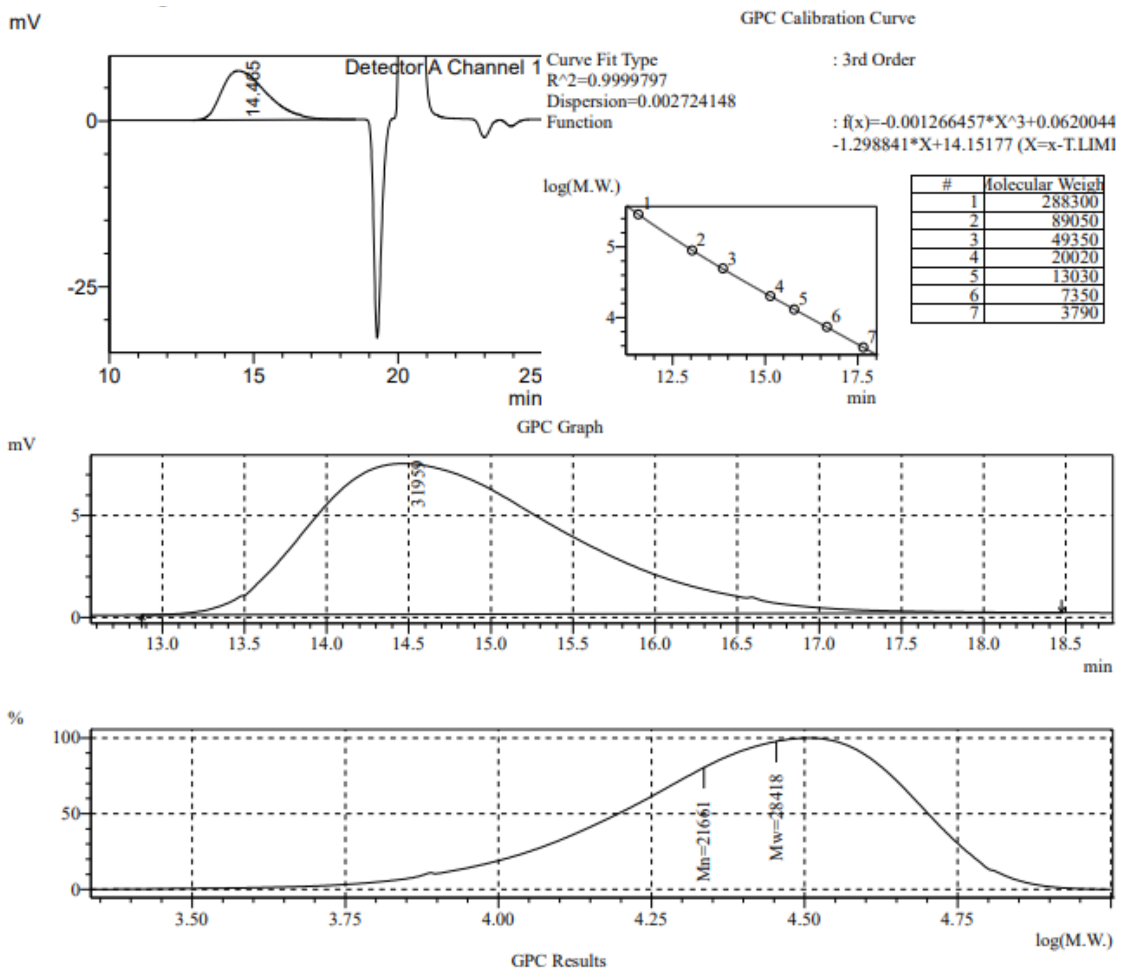

#	lolecular Weigh
1	288300
2	89050
3	49350
4	20020
5	13030
6	7350
7	3790

GPC Results

Peak#:1 (Detector A Channel 1)

[Peak Information]

Title	Time(min)	lolecular Weigl	Height
Start	12.875	101039	117
Top	14.465	31959	7400
End	18.475	2153	223

Area : 808265

[Average Molecular Weight]

Number Average Molecular Weight(Mn)	21661
Weight Average Molecular Weight(Mw)	28418
Mw/Mn	1.31196

Figure S52: GPC trace of P(**M1**) in DMSO-d_6 before heating. P(**M1**) was obtained with **M1**/Y-1/BnOH = 100/1/2 and used calibration; $M_{n,GPC}$ = 21.7 g mol^{-1}, Đ = 1.31.

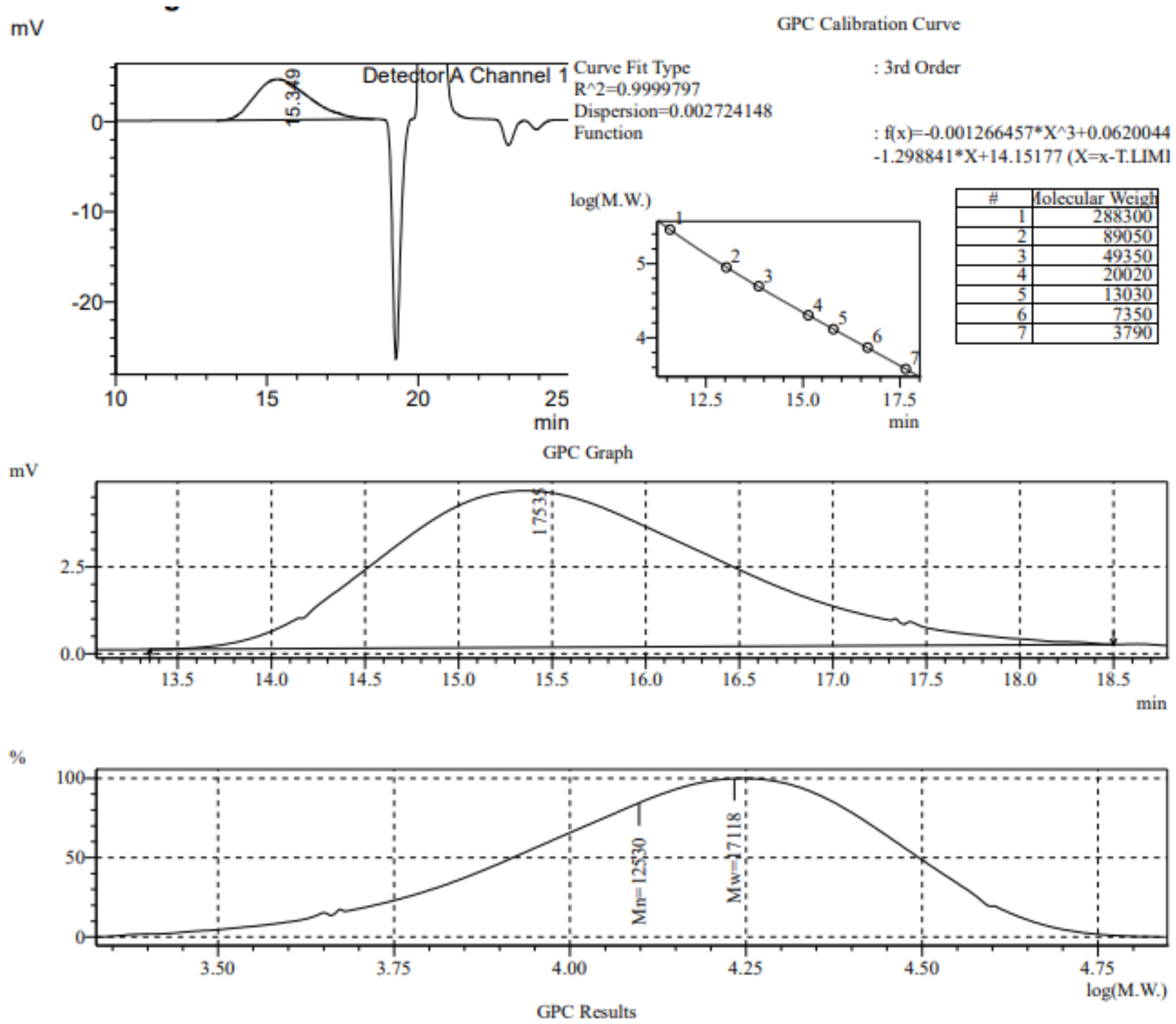

#	Iolecular Weigh
1	288300
2	89050
3	49350
4	20020
5	13030
6	7350
7	3790

Peak#:1 (Detector A Channel 1)

[Peak Information]

Title	Time(min)	Iolecular Weigl	Height
Start	13.350	70728	124
Top	15.349	17535	4508
End	18.500	2116	263

Area : 564482

[Average Molecular Weight]

Number Average Molecular Weight(Mn)	12530
Weight Average Molecular Weight(Mw)	17118
Mw/Mn	1.36622

Figure S53: GPC trace of P(**M1**) in DMSO-d_6 after heating at 100 °C for 1 h and used calibration; $M_{n,GPC}$ 12.5 kg mol^{-1}; Đ = 1.37

NMR spectra and GPC traces of chemical recycling studies of P(M1)

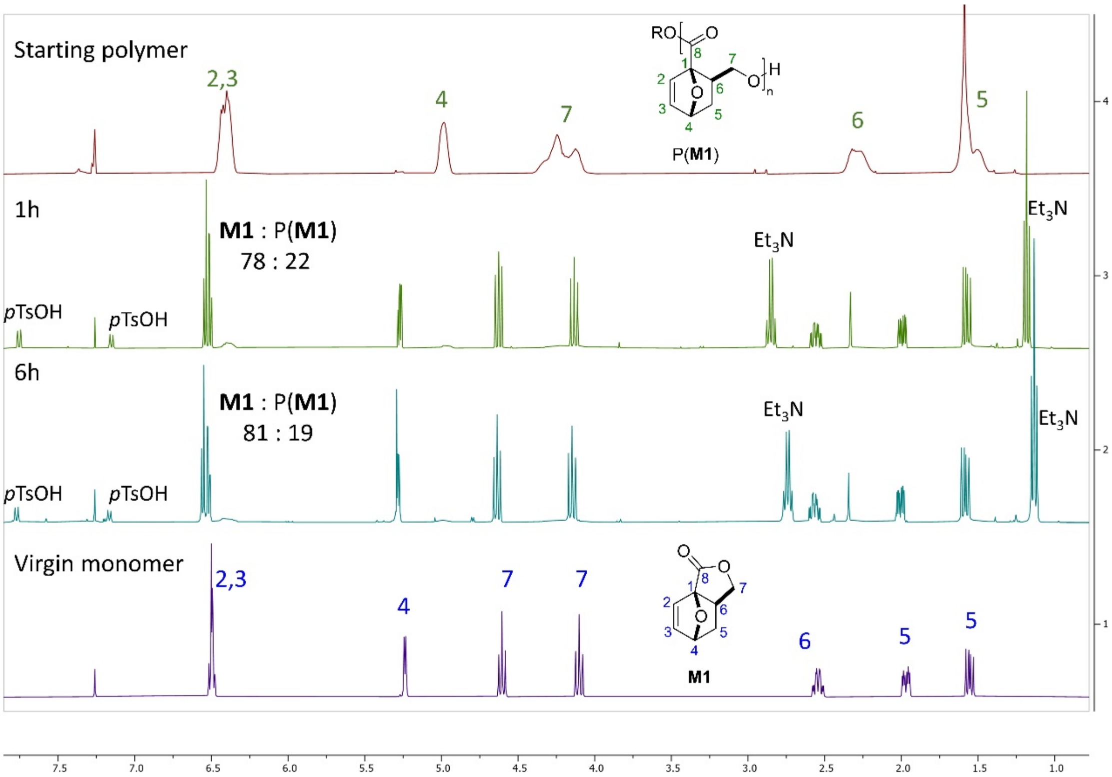

Figure S54: Stacked ^{1}H NMR spectra for CRM of P(**M1**). From top to bottom: Starting P(**M1**) formed by Y-**1** and BnOH (conditions of Table 1 entry 11); CRM after 1 h (78% conversion); CRM after 6 h (81% conversion); Reference spectrum of virgin **M1**.

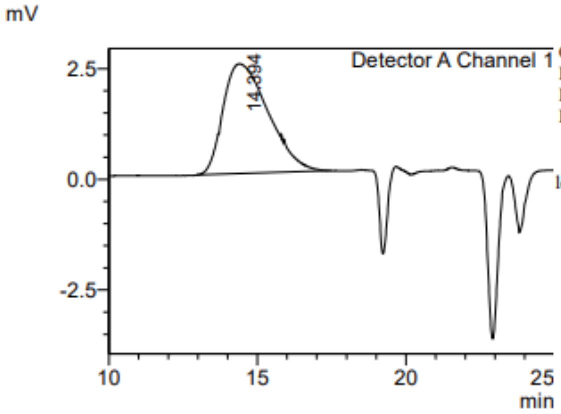

GPC Calibration Curve

Curve Fit Type : 3rd Order
R^2=0.9999830
Dispersion=0.002488804
Function : f(x)=-0.001236626*X^3+0.0602274 -1.267230*X+13.96510 (X=x-T.LIMI

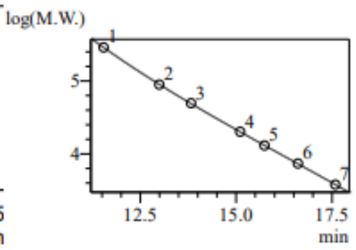

#	Iolecular Weigh
1	288300
2	89050
3	49350
4	20020
5	13030
6	7350
7	3790

GPC Graph

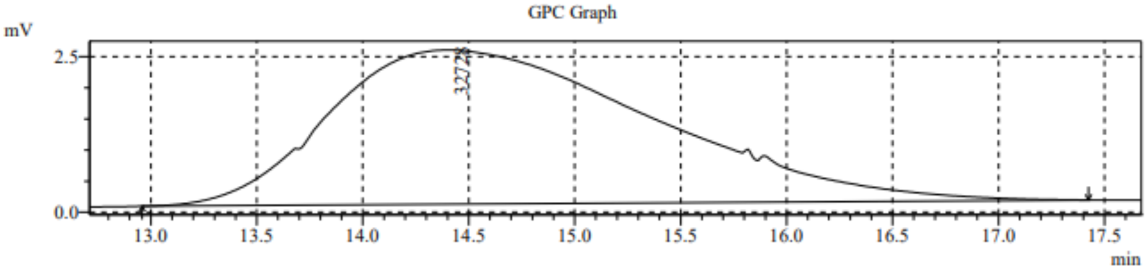

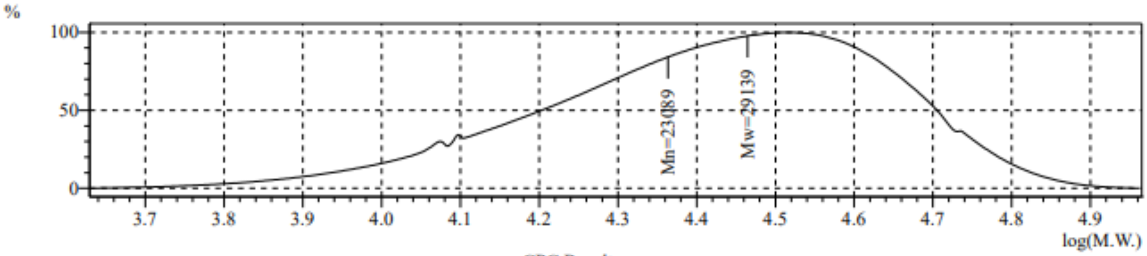

GPC Results

Peak#:1 (Detector A Channel 1)

[Peak Information]

Title	Time(min)	Iolecular Weigl	Height
Start	12.958	92550	98
Top	14.394	32728	2478
End	17.425	4244	195

Area : 262422

[Average Molecular Weight]

Number Average Molecular Weight(Mn)	23089
Weight Average Molecular Weight(Mw)	29139
Mw/Mn	1.26207

Figure S55: GPC trace of starting polymer for CRM of P(**M1**) (M_n = 23.1 kg mol^{-1}, Đ = 1.26). Formed by Y-**1** and BnOH (according to conditions of Table 1, entry 11).

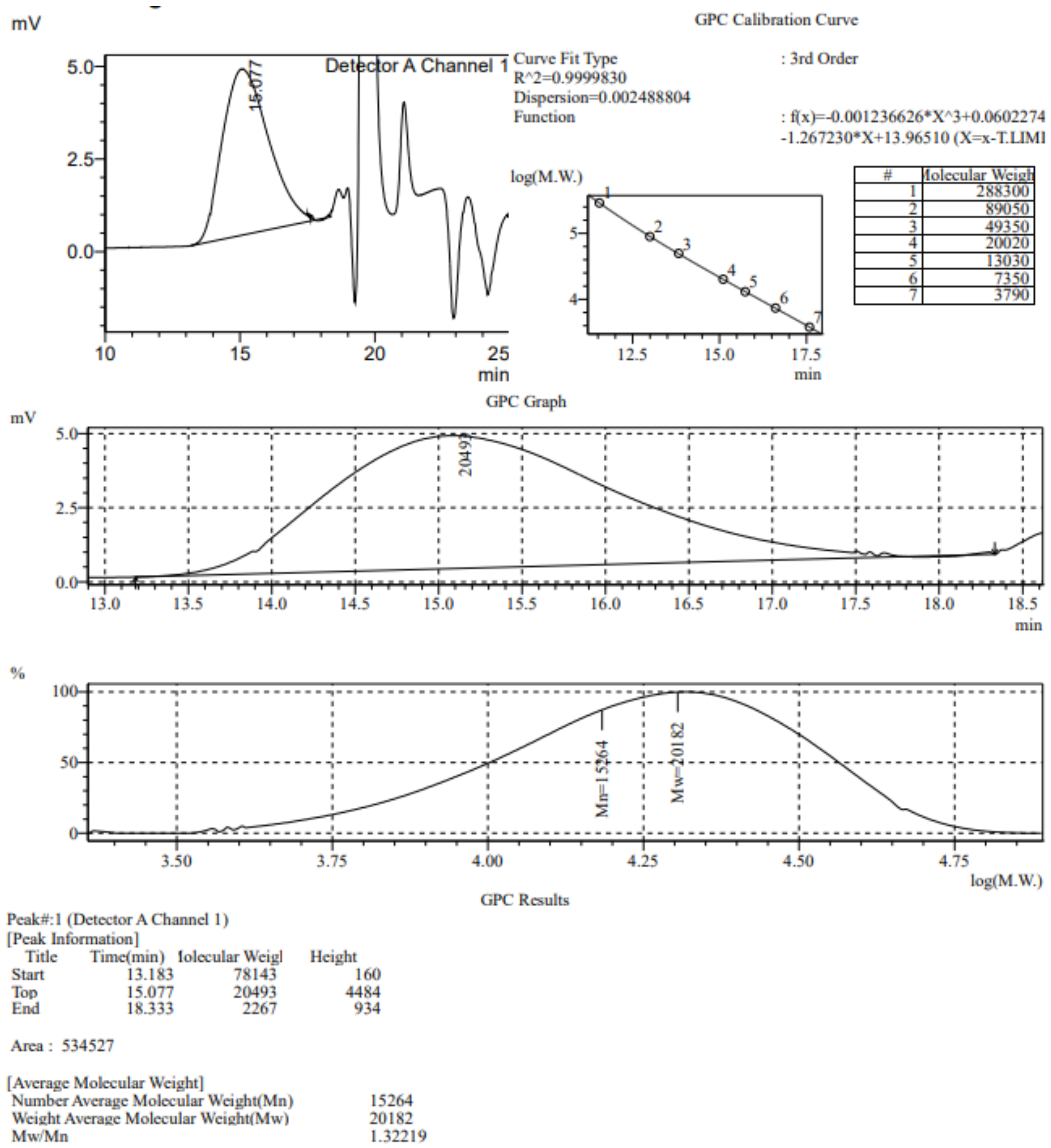

Figure S56: GPC trace of analysis during CRM of P(**M1**) (reaction time 1 h). M_n residual polymer = 15.3 kg/mol, Đ = 1.32

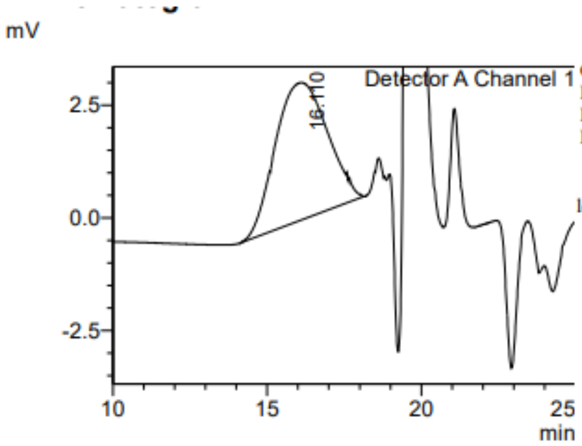

GPC Calibration Curve

Curve Fit Type : 3rd Order
R^2=0.9999830
Dispersion=0.002488804
Function : f(x)=-0.001236626*X^3+0.0602274
-1.267230*X+13.96510 (X=x-T.LIMI

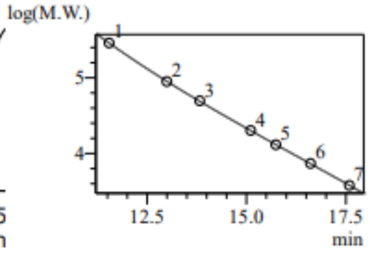

#	Iolecular Weigh
1	288300
2	89050
3	49350
4	20020
5	13030
6	7350
7	3790

GPC Graph

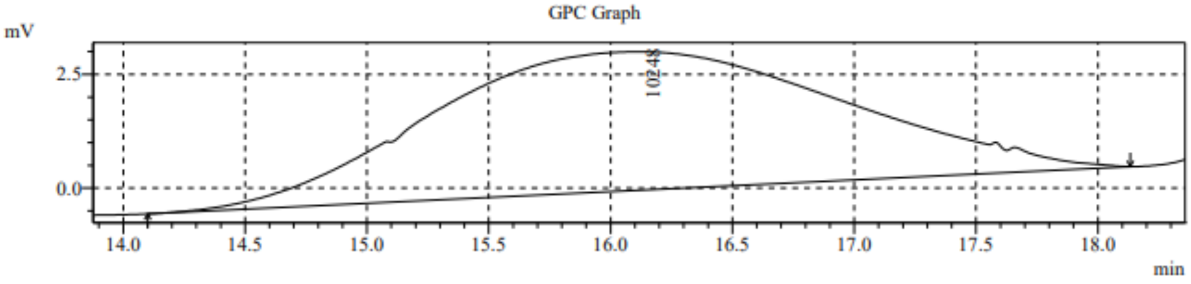

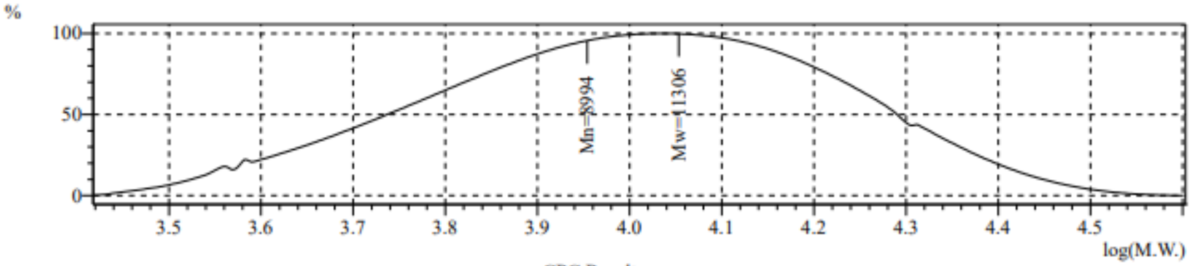

GPC Results

Peak#:1 (Detector A Channel 1)
[Peak Information]

Title	Time(min)	Iolecular Weigl	Height
Start	14.100	40220	-567
Top	16.110	10248	3047
End	18.133	2608	471

Area : 358932

[Average Molecular Weight]

Number Average Molecular Weight(Mn)	8994
Weight Average Molecular Weight(Mw)	11306
Mw/Mn	1.25711

Figure S57: GPC trace of analysis during CRM of P(**M1**) (reaction time 6 h). M_n residual polymer = 9.0 kg mol^{-1}, Đ = 1.26

NMR spectra of chemical recycling studies of P(M1-H_2)

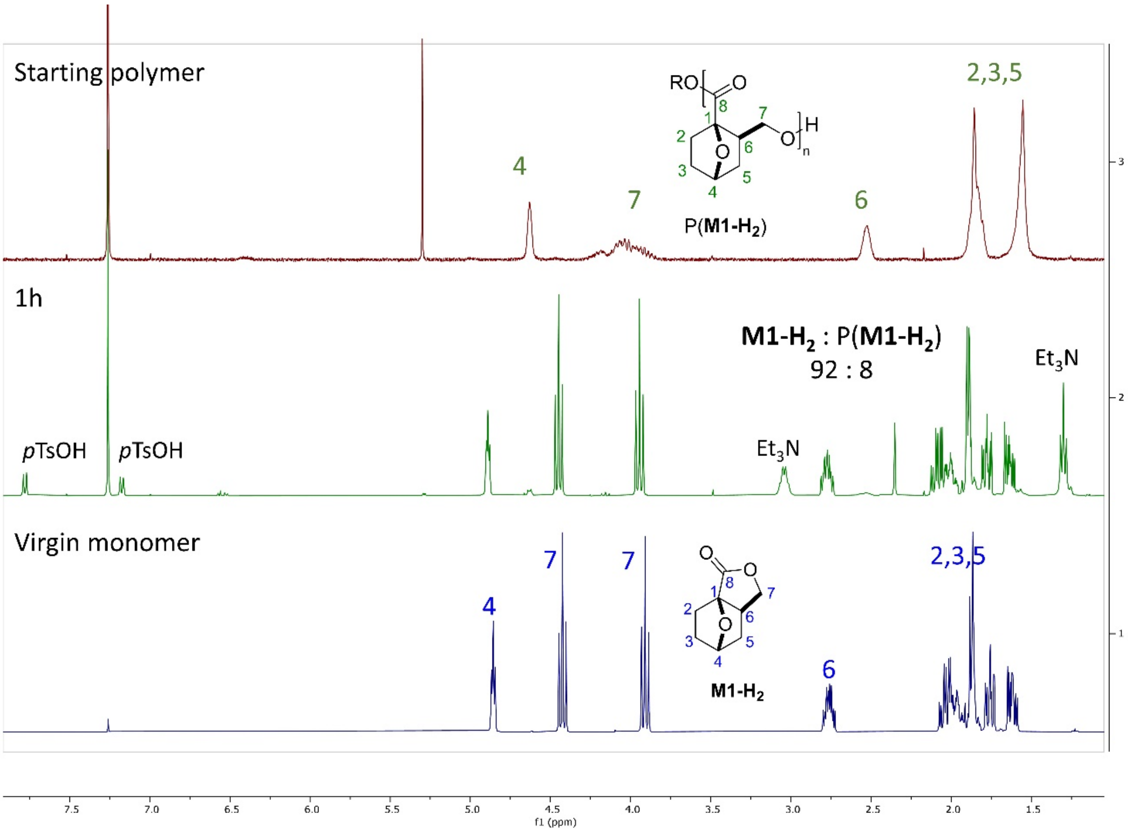

Figure S58: Stacked ^{1}H NMR spectra for CRM of P(**M1-H$_2$**). From top to bottom: Starting P(**M1-H$_2$**) formed by hydrogenation of P(**M1**); CRM after 1 h (92% conversion); Reference spectrum of virgin **M1-H$_2$**.

References

1. Cai, C. X., Toupet, L., Lehmann, C. W. & Carpentier, J. F. Synthesis, structure and reactivity of new yttrium bis(dimethylsilyl) amido and bis(trimethylsilyl)methyl complexes of a tetradentate bis(phenoxide) ligand. *J. Organomet. Chem.* **683**, 131–136 (2003).

2. Fulmer, G. R. *et al.* NMR chemical shifts of trace impurities: Common laboratory solvents, organics, and gases in deuterated solvents relevant to the organometallic chemist. *Organometallics* **29**, 2176–2179 (2010).

3. Cioc, R. C., Harsevoort, E., Lutz, M. & Bruijnincx, P. C. A. Efficient synthesis of fully renewable, furfural-derived building blocks via formal Diels-Alder cycloaddition of atypical addends. *Green Chem.* **25**, 9689–9694 (2023).

4. Lancefield, C. S. *et al.* Dynamic Trapping as a Selective Route to Renewable Phthalide from Biomass-Derived Furfuryl Alcohol. *Angew. Chemie - Int. Ed.* **59**, 23480–23484 (2020).

5. Duda, A. & Kowalski, A. *Handbook of Ring-Opening Polymerization: Thermodynamics and Kinetics of Ring - Opening Polymerization*. (2009).

6. Szigeti, M., Dobi, Z. & Soós, T. The Goldilocks Principle in Phase Labeling. Minimalist and Orthogonal Phase Tagging for Chromatography-Free Mitsunobu Reaction. *J. Org. Chem.* **83**, 2869–2874 (2018).

7. Schreurs, A. M. M., Xian, X. & Kroon-Batenburg, L. M. J. EVAL15: A diffraction data integration method based on ab initio predicted profiles. *J. Appl. Crystallogr.* **43**, 70–82 (2010).

8. Krause, L., Herbst-Irmer, R., Sheldrick, G. M. & Stalke, D. Comparison of silver and molybdenum

microfocus X-ray sources for single-crystal structure determination. *J. Appl. Crystallogr.* **48**, 3–10 (2015).

9. Sheldrick, G. M. SHELXT - Integrated space-group and crystal-structure determination. *Acta Crystallogr.* **A71**, 3–8 (2015).

10. Sheldrick, G. M. Crystal structure refinement with SHELXL. *Acta Crystallogr.* **C71**, 3–8 (2015).

11. Spek, A. L. Structure validation in chemical crystallography. *Acta Crystallogr.* **D65**, 148–155 (2009).

12. Frisch, M. J. *et al.* G16_C01. Gaussian 16, Revision C.01, Gaussian, Inc., Wallin at (2016).

13. Grimme, S., Antony, J., Ehrlich, S. & Krieg, H. A consistent and accurate ab initio parametrization of density functional dispersion correction (DFT-D) for the 94 elements H-Pu. *J. Chem. Phys.* **132**, (2010).

14. Hong, M. & Chen, E. Y. X. Completely recyclable biopolymers with linear and cyclic topologies via ring-opening polymerization of γ-butyrolactone. *Nat. Chem.* **8**, 42–49 (2016).

15. Zhu, J. B., Watson, E. M., Tang, J. & Chen, E. Y. X. A synthetic polymer system with repeatable chemical recyclability. *Science* **360**, 398–403 (2018).

16. Haba, O. & Itabashi, H. Ring-opening polymerization of a five-membered lactone trans-fused to a cyclohexane ring. *Polym. J.* **46**, 89–93 (2014).

17. Shi, C. *et al.* Hybrid monomer design for unifying conflicting polymerizability, recyclability, and performance properties. *Chem* **7**, 670–685 (2021).

18. Shi, C., Clarke, R. W., McGraw, M. L. & Chen, E. Y. X. Closing the 'one Monomer-Two Polymers-One Monomer' Loop via Orthogonal (De)polymerization of a Lactone/Olefin Hybrid. *J. Am. Chem. Soc.* **144**, 2264–2275 (2022).
